# Artificial intelligence-driven prediction of COVID-19-related hospitalization and death: a systematic review

**DOI:** 10.3389/fpubh.2023.1183725

**Published:** 2023-06-20

**Authors:** Saeed Shakibfar, Fredrik Nyberg, Huiqi Li, Jing Zhao, Hedvig Marie Egeland Nordeng, Geir Kjetil Ferkingstad Sandve, Milena Pavlovic, Mohammadhossein Hajiebrahimi, Morten Andersen, Maurizio Sessa

**Affiliations:** ^1^Pharmacovigilance Research Center, Department of Drug Design and Pharmacology, University of Copenhagen, Copenhagen, Denmark; ^2^School of Public Health and Community Medicine, Institute of Medicine, Sahlgrenska Academy, University of Gothenburg, Gothenburg, Sweden; ^3^Pharmacoepidemiology and Drug Safety Research Group, Department of Pharmacy, Faculty of Mathematics and Natural Sciences, University of Oslo, Oslo, Norway; ^4^UiORealArt Convergence Environment, University of Oslo, Oslo, Norway; ^5^Department of Informatics, Faculty of Mathematics and Natural Sciences, University of Oslo, Oslo, Norway; ^6^Department of Pharmacy, Pharmacoepidemiology and Social Pharmacy, Uppsala University, Uppsala, Sweden

**Keywords:** AI, COVID-19, pharmacoepidemiology, bias, PROBAST, predictive modeling

## Abstract

**Aim:**

To perform a systematic review on the use of Artificial Intelligence (AI) techniques for predicting COVID-19 hospitalization and mortality using primary and secondary data sources.

**Study eligibility criteria:**

Cohort, clinical trials, meta-analyses, and observational studies investigating COVID-19 hospitalization or mortality using artificial intelligence techniques were eligible. Articles without a full text available in the English language were excluded.

**Data sources:**

Articles recorded in Ovid MEDLINE from 01/01/2019 to 22/08/2022 were screened.

**Data extraction:**

We extracted information on data sources, AI models, and epidemiological aspects of retrieved studies.

**Bias assessment:**

A bias assessment of AI models was done using PROBAST.

**Participants:**

Patients tested positive for COVID-19.

**Results:**

We included 39 studies related to AI-based prediction of hospitalization and death related to COVID-19. The articles were published in the period 2019-2022, and mostly used Random Forest as the model with the best performance. AI models were trained using cohorts of individuals sampled from populations of European and non-European countries, mostly with cohort sample size <5,000. Data collection generally included information on demographics, clinical records, laboratory results, and pharmacological treatments (i.e., high-dimensional datasets). In most studies, the models were internally validated with cross-validation, but the majority of studies lacked external validation and calibration. Covariates were not prioritized using ensemble approaches in most of the studies, however, models still showed moderately good performances with Area under the Receiver operating characteristic Curve (AUC) values >0.7. According to the assessment with PROBAST, all models had a high risk of bias and/or concern regarding applicability.

**Conclusions:**

A broad range of AI techniques have been used to predict COVID-19 hospitalization and mortality. The studies reported good prediction performance of AI models, however, high risk of bias and/or concern regarding applicability were detected.

## 1. Introduction

Coronavirus Disease 2019 (COVID-19) was declared a global pandemic on 11th March 2020 by the World Health Organization (WHO) (1). In 2020 COVID-19 spread all over the world and has already infected more than 623 million individuals and caused more than 6 million deaths worldwide (2) and, in the U.S., more than 5 million hospitalizations by the 1st of September, 2022 (3).

Huge efforts has been made by the scientific community to promote the integration of artificial intelligence (AI) into predictive modeling of COVID-19-related outcomes (4). Artificial intelligence is defined as “*the theory and development of computer systems able to perform tasks normally requiring human intelligence*” (5). Within AI, especially machine learning which is defined as “*the theory and development of automatic discovery of regularities in data through the use of computer algorithms and with the use of these regularities to take actions such as classifying the data into different categories*”, has the potential of achieving high prediction accuracy and scalability of models based on data availability (6). This is crucial in fast-pacing scenarios such as the COVID-19 pandemic (3).

Considering that none of the available reviews on the use of AI in the predictive modeling of COVID-19-related outcomes have performed a bias/applicability assessment of AI models used to predict such outcomes (7–14) (Table 1), we conducted a systematic screening aiming at filling this knowledge gap. According to the definitions of the tool used for assessing the risk of bias in this systematic review (i.e., Bias analysis using the Prediction model Risk Of Bias ASsessment Tool, *PROBAST*), bias was defined to occur when shortcomings in the study design, conduct, or analysis lead to systematically distorted estimates of model predictive performance. Concerns regarding applicability of AI were considered high when the population, predictors, or outcomes of the study differ from those specified in the review question.

**Table 1:** Previous systematic reviews on the application of AI for COVID-19 research.

**Author, publication year**	**Number of articles**	**Objective**	**Comparison of information provided in previous vs. those provide in this review**			
Saleem et al., 2022 (7)	57	To highlight the latest developments in analyzing the COVID-19 data using machine learning and deep learning algorithms.	**Articles with the following outcomes** ✓Hospitalization ✓Mortality	**Data-related aspects** ✓Type of data ✓Country ✓Single/multi-country data sources x Type of covariates x Primary vs. secondary data	**AI models-related aspects** ✓Model ✓Model's performance ✓External validation x Internal validation x Type of filtering/prioritization x Supervised/not supervised x Calibration x Bias according to the prediction model risk of bias assessment tool	**Epidemiological aspects** x Nationwide/sampled population x Risk factors for mortality x Risk factors for hospitalization x Disease risk score for severe COVID-19 disease (death or hospitalization)
Napolitano et al., 2022 (8)	17 269	To identify the application of machine learning methods on COVID-19.	**Articles with the following outcomes** ✓Hospitalization ✓Mortality	**Data-related aspects** x Type of data x Country x Single/multi-country data sources ✓Type of covariates x Primary vs. secondary data	**AI models-related aspects** ✓Model ✓Model's performance x External validation x Internal validation x Type of filtering/prioritization x Supervised/not supervised x Calibration x Bias according	**Epidemiological aspects** x Nationwide/sampled population ✓Risk factors for mortality ✓Risk factors for hospitalization x Disease risk score for severe COVID-19 disease (death or hospitalization)
Lyu et al., 2022 (9)	19	To analyze the papers that used artificial intelligence (AI) models to forecast COVID-19 outcomes	**Articles with the following outcomes** ✓Hospitalization ✓Mortality	**Data-related aspects** x Type of data ✓Country x Single/multi-country data sources x Type of covariates x Primary vs. secondary data	**AI models-related aspects** ✓Model ✓Model's performance x External validation x Internal validation x Type of filtering/prioritization x Supervised/not supervised x Calibration x Bias according to the prediction model risk of bias assessment tool	**Epidemiological aspects** x Nationwide/sampled population x Risk factors for mortality x Risk factors for hospitalization x Disease risk score for severe COVID-19 disease (death or hospitalization)
Bottino et al., 2021 (10)	24	To look into the studies that implemented machine learning, including deep learning, methods in COVID mortality prediction	**Articles with the following outcomes** x Hospitalization ✓Mortality	**Data-related aspects** ✓Type of data ✓Country ✓Single/multi-country data sources ✓Type of covariates Primary vs. secondary data	**AI models-related aspects** ✓Model ✓Model's performance ✓External validation ✓Internal validation ✓Type of filtering/prioritization ✓Supervised/not supervised x Calibration x Bias according to the prediction model risk of bias assessment tool	**Epidemiological aspects** x Nationwide/sampled population ✓Risk factors for mortality x Risk factors for hospitalization x Disease risk score for severe COVID-19 disease (death or hospitalization)
Guo et al., 2021 (11)	794	To summarize how artificial intelligence (AI) is being applied in COVID-19 research and determine whether these AI applications integrated heterogeneous data from different sources for modeling.	**Articles with the following outcomes** ✓Hospitalization ✓Mortality	**Data-related aspects** ✓Type of data ✓Country ✓Single/multi-country data sources ✓Type of covariates x Primary vs. secondary data	**AI models-related aspects** ✓Model x Model's performance x External validation x Internal validation x Type of filtering/prioritization x Supervised/not supervised x Calibration x Bias according to the prediction model risk of bias assessment tool	**Epidemiological aspects** x Nationwide/sampled population x Risk factors for mortality x Risk factors for hospitalization x Disease risk score for severe COVID-19 disease (death or hospitalization)
Shi et al., 2021 (12)	27	To assess the potential predictors of mortality in patients with COVID-19.	**Articles with the following outcomes** x Hospitalization ✓Mortality	**Data-related aspects** ✓Type of data ✓Country x Single/multi-country data sources ✓Type of covariates x Primary vs. secondary data	**AI models-related aspects** ✓Model ✓Model's performance x External validation x Internal validation x Type of filtering/prioritization x Supervised/not supervised x Calibration xBias according to the prediction model risk of bias assessment tool	**Epidemiological aspects** x Nationwide/sampled population ✓Risk factors for mortality x Risk factors for hospitalization x Disease risk score for severe COVID-19 disease (death or hospitalization)
Syed et al., 2021 (13)	8	To identify the deep learning techniques that have been applied to predict hospital mortality in COVID-19 patients.	**Articles with the following outcomes** x Hospitalization ✓Mortality	**Data-related aspects** ✓Type of data x Country x Single/multi-country data sources ✓Type of covariates x Primary vs. secondary data	**AI models-related aspects** ✓Model ✓Model's performance x External validation x Internal validation x Type of filtering/prioritization x Supervised/not supervised x Calibration x Bias according to the prediction model risk of bias assessment tool	**Epidemiological aspects** x Nationwide/sampled population x Risk factors for mortality x Risk factors for hospitalization x Disease risk score for severe COVID-19 disease (death or hospitalization)
Alballa et al., 2021 (14)	52	To focus on the potential of ML for two main applications: diagnosis of COVID-19 and prediction of mortality risk and severity, using readily available clinical and laboratory data.	**Articles with the following outcomes** x Hospitalization ✓Mortality	**Data-related aspects** ✓Type of data x Country x Single/multi-country data sources ✓Type of covariates x Primary vs. secondary data	**AI models-related aspects** ✓Model ✓Model's performance ✓External validation ✓Internal validation ✓Type of filtering/prioritization ✓Supervised/not supervised x Calibration x Bias according to the prediction model risk of bias assessment tool	**Epidemiological aspects** x Nationwide/sampled population ✓Risk factors for mortality x Risk factors for hospitalization ✓Disease risk score for severe COVID-19 disease (death or hospitalization)

We performed a systematic literature search in Ovid MEDLINE to identify studies using AI models to predict COVID-19-related hospitalization/mortality. AI-, data-, and epidemiological-related aspects were extracted and assessed as these aspects are critical for the scientific robustness of the published articles.

## 2. Methods

### 2.1. Search methods

Ovid MEDLINE (from 01/01/2019 to 2022/08/22) was searched, along with the reference lists in the reviews identified with our research query (Supplementary Table 1). Search terms included in the query have been previously used in the context of systematic reviews of AI/ML models and were described by Sessa et al. elsewhere (15, 16). This review was performed according to the Preferred Reporting Items for Systematic Reviews and Meta-Analyses (PRISMA) guidelines (17). The PRISMA checklist is provided in Supplementary Table 2.

### 2.2. Eligibility criteria

We evaluated observational studies, meta-analyses, and clinical trials that developed, validated, or updated machine learning prognostic prediction models for COVID-19-related hospitalization and mortality. However, we excluded studies providing an overview and epidemiological modeling tasks such as predicting COVID-19 peaks. Additionally, we excluded articles focusing on the use of AI for image, signal, or time-series processing for prediction of COVID-19-related outcomes and those articles focusing on the use of AI for assessing the safety/effectiveness of vaccines for COVID-19 or drug discovery for COVID-19 treatments. Finally, we excluded studies focusing on AI algorithms development that did not test the algorithm using clinical data. Only studies for which the full text was available in the English language were considered eligible. Abstracts sent to international or national conferences, letters to the editor, and case reports/series were considered ineligible along with articles evaluating natural language processing techniques. The reference list of narrative and systematic reviews included with our MEDLINE query were further screened for undetected records.

### 2.3. Selection of studies

In the first screening procedure, titles and abstracts of retrieved records were screened by two independent researchers (SS&MS). All articles that were considered eligible at the first screening procedure underwent a full-text evaluation. If disagreements regarding eligibility of the articles arose during the two steps evaluation process, it was resolved by consensus.

### 2.4. Aims

The primary aim of the systematic review was the assessment of biases in retrieved articles and in depth description of the AI models used for covid-19 risk prediction. This included a description of their performance, their application for external/internal validation, the type of filtering/prioritization approach that was used, and the approaches for models' learning (i.e., supervised/not supervised). Additionally, we investigated the use of calibration, and models' bias assessment tools, the type of data (i.e., country where the data were generated, single/multi-country data sources, type of covariates generated from the data, and primary vs. secondary data), and epidemiological aspects of AI models' application (i.e., nationwide/sampled population, risk factors/disease risk score development for COVID-19-related mortality/hospitalization). The secondary aim was the visualization (plots/tables) of the information described for the primary aim and an outline of why the bias occurred, and what the main sources of bias were.

### 2.5. Data extraction and synthesis

A data extraction form was developed for this systematic review (Supplementary Table 3).

#### 2.5.1. Data-related aspects

We extracted the following data-related information from each eligible study:

1) *Type of data;*

Type of data was defined as: “a particular kind of data item, as defined by the values it can take, the programming language used, or the operations that can be performed on it” (18). We extracted and categorized data into four data types: pharmacological treatments [e.g., drug prescriptions], clinical (e.g., signs, symptoms, physician notes, and patients' diagnoses), laboratory (biochemical or immunological laboratory test results), and demographic data (e.g., age and gender).

2) *Single or multi-countries data sources;*

A multi-country data source was defined as a data collection process from more than one country.

3) *Country of data collection;*

Country of data collection was defined as a nation with its own government, occupying a particular territory where data were collected .

4) *Type of covariates;*

A covariate was defined as an independent variable that can influence the outcome of a given statistical trial (19). In this review these consisted of predictors of hospitalization/death due to COVID-19 and potential confounders of these predictors. Covariates were extracted as presented in the statistical analysis section of retrieved articles.

5) *Primary or secondary data;*

We defined primary data as information collected directly by the researchers using interviews –personal or by telephone– or self-administered questionnaires (20). Secondary data were defined as data that were previously collected for other purposes than for the study at hand (20).

#### 2.5.2. AI model-related aspects

We extracted the following information directly related to the AI modeling for each study:

6) *Model;*

We defined a statistical model as a mathematical model that embodies a set of statistical assumptions concerning the generation of sample data (and similar data from a larger population) (21).

7) *Model performance;*

Model performance was assessed using methods and metrics described by Steyerberg et al. (22). In this review, we used traditional measures for assessing overall model performance [e.g., area under the receiver operating characteristic curve (AUC) and goodness-of-fit statistics for calibration as reported in the original studies (22)].

8) *Internal/External validation;*

Internal validity was defined as the extent to which the observed results represent the truth in the population actually studied and, thus, are not due to methodological errors. External validity refers to the extent to which the results of a study are generalizable to patients in daily practice outside the study population, especially for the population that the sample is thought to represent (23).

9) *Type of filtering/prioritization;*

Filtering/prioritization exert the process of over-emphasizing or censoring certain information based on their perceived importance (24).

10) *Supervised/unsupervised machine learning;*

Supervised learning refers to techniques in which a model is trained on a range of inputs (or features) which are associated with a known outcome (25).

11) *Calibration;*

Calibration was defined as a procedure in statistical classification to determine class membership probabilities which assess the uncertainty of assigning a given new observation into established classes (26).

12) *Bias analysis using the Prediction model Risk Of Bias ASsessment Tool (PROBAST).*,

PROBAST was used as a systematic bias assessment tool for assessing the risk of bias and applicability of prediction models in the retrieved studies according to the procedures described by Wolff et al. (27).

#### 2.5.3. Epidemiological aspects

We extracted the following epidemiological-related information for each study:

13) *Nationwide/sampled population;*

We defined nationwide data as data that were available for the entire population in a specific geographical region.

14) *Risk factors for hospitalization/mortality;*

Risk factors for hospitalization/mortality were defined as a list of predictors to identify positive tested COVID-19 patients at high risk of hospitalization or mortality.

15) *Disease risk score for severe COVID-19 disease (death or hospitalization);*

Disease risk score was defined as a summary measure derived from the observed values of the risk factors that were able to predict severe outcomes of COVID-19.

### 2.6. PROBAST

Two researchers (SS&MS) independently used PROBAST to assess the risk of bias and applicability of prognostic prediction models in the included studies. If multiple prognostic prediction models were reported in a study, only the model with the best predictive performance was considered. The PROBAST statement was divided into four domains: participants, predictors, outcome, and analysis. These domains contain a total of 20 signal questions to help structure judgment of risk of bias for prediction models, such as the range of the included patients, whether the same predictors and results were defined for all participants, whether the clinical decision rules were determined prospectively, and whether a relevant measure of accuracy was reported (27). Additionally, PROBAST requires an assessment of the applicability of models when the population, predictors, or outcomes of the study differ from those specified in the review question.

### 2.7. Data analysis

For the secondary aims, descriptive analysis and visualization was performed using R 4.2.1 (28).

## 3. Results

**Figure 1:**
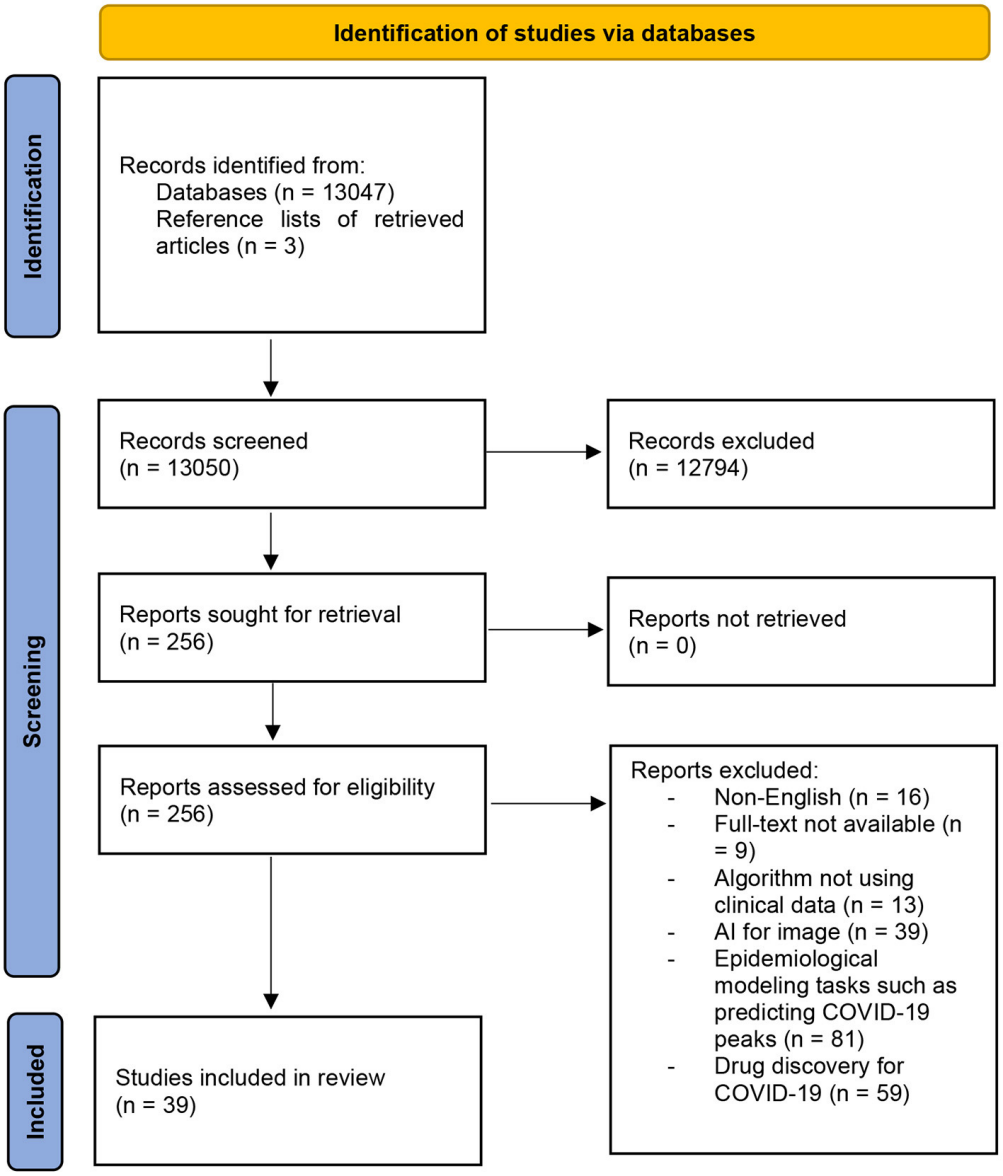
Flowchart of the study selection process.

We retrieved 13,050 studies of which 12,794 studies were excluded according to the exclusion criteria detailed. After reading the full texts, an additional 217 studies (13,011 total) were excluded. We identified 3 additional studies by checking reference lists of the retrieved articles and literature reviews that were excluded. In total, we included 39 studies for bias assessment and data analysis (Figure 1).

### 3.1. Descriptive analysis

We included 39 studies related to AI-based prediction of hospitalization and death related to COVID-19. In all, 27 studies used AI to predict COVID-19 mortality, 9 studies used AI to predict COVID-19-related hospitalization, and 3 studies had both outcomes.

#### 3.1.1. Data-related aspects

**Figure 2:**
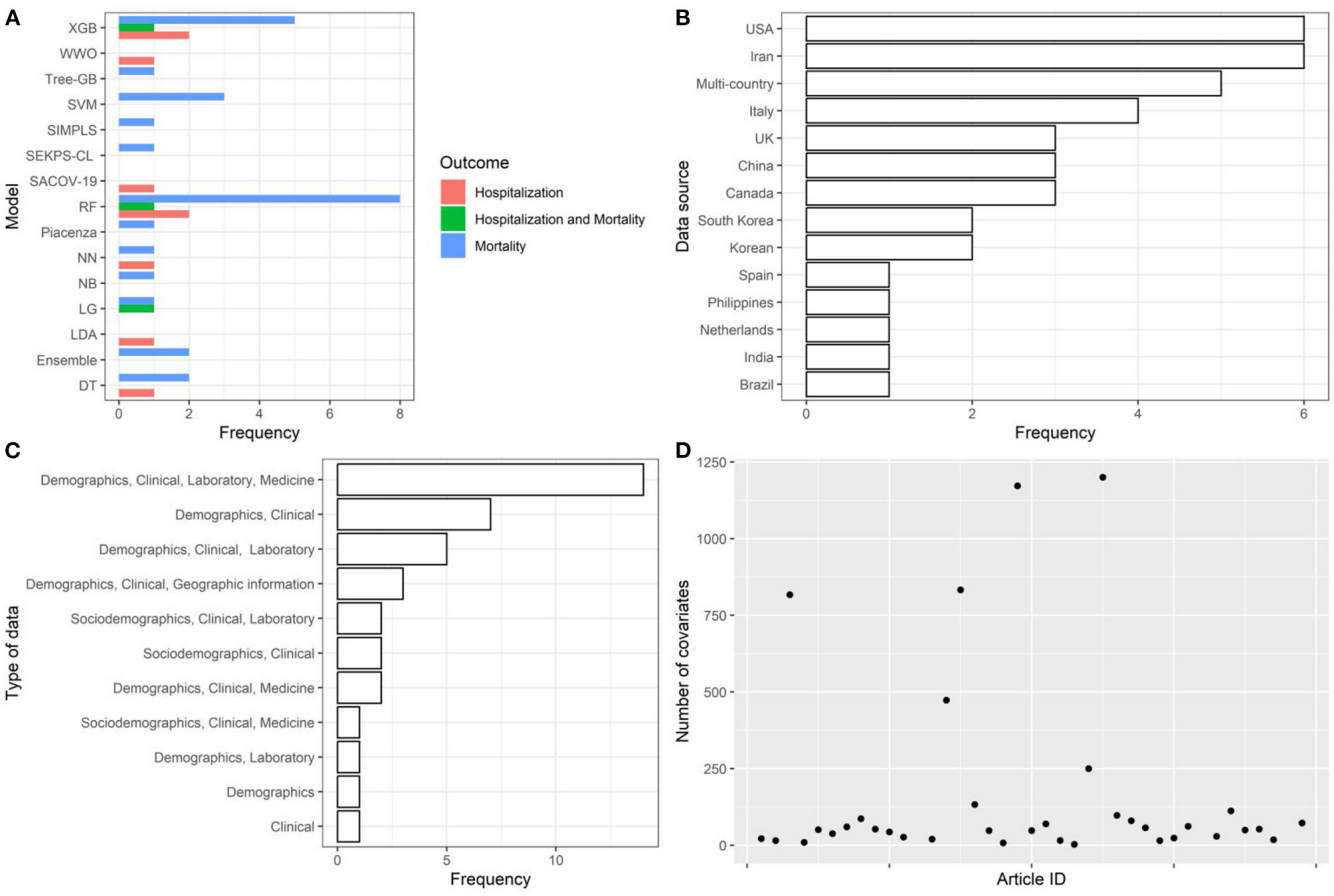
Descriptive analysis—part 1. **(A)**: models, **(B)**: data source, **(C)**: type of data, **(D)**: number of covariates. Extreme Gradient Boosting (XGB), Water Wave Optimization (WWO), Support Vector Machine (SVM), Inspired Modification of Partial Least Square (SIMPLS), SEKPS-CL, Random Forest (RF), Neural Network (NN), Naive Bayes (NB), Logistic Regression (LR), Linear Discriminant Analysis (LDA), Ensemble, Decision Tree (DT), Disease Risk Score.

AI models were trained using cohorts of individuals sampled from populations of European and non-European countries, mostly with cohort sample size <5,000. Data collection generally included information on demographics, clinical records, laboratory results, and pharmacological treatments (i.e., high-dimensional datasets—only 3 studies with more than 1,200 covariates of which one omitted from Figure 2).

#### 3.1.2. AI model-related aspects and epidemiological aspects

**Figure 3:**
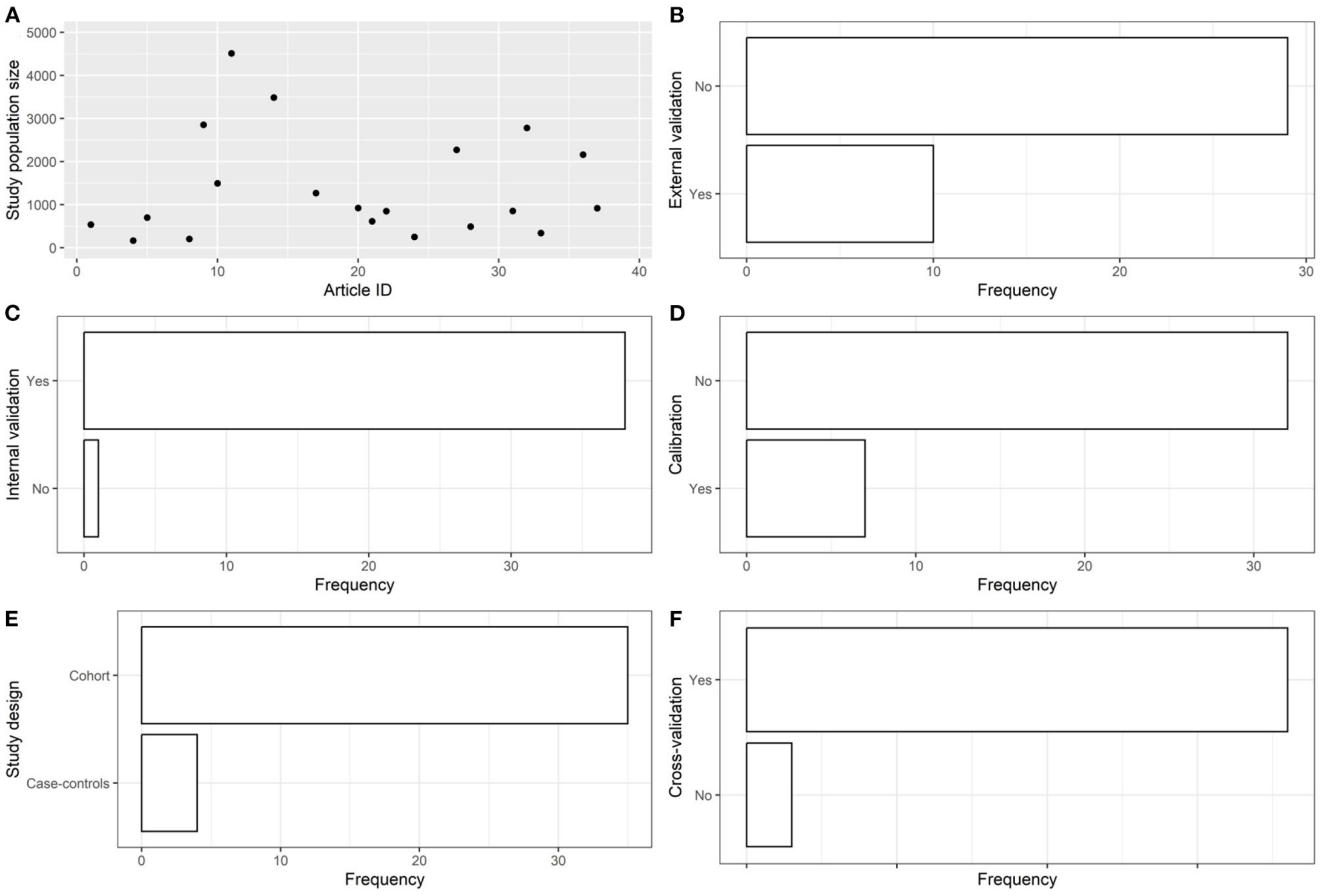
Descriptive analysis - part 2. **(A)**: study population size, **(B)**: using external validation, **(C)**: using internal validation, **(D)**: using calibration, **(E)**: study design, **(F)**: using cross-validation.

**Figure 4:**
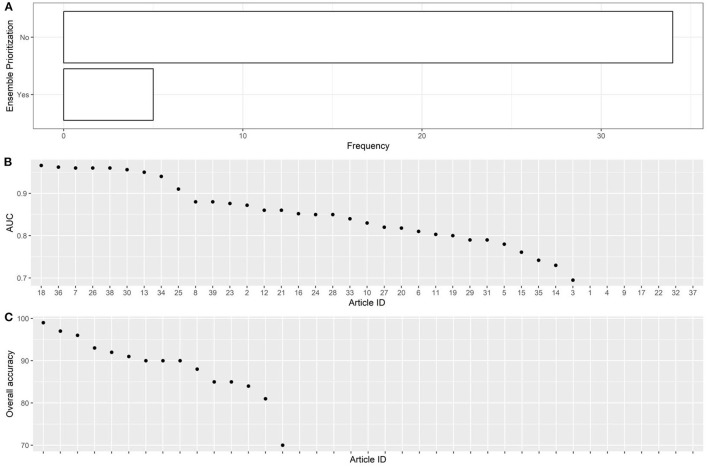
Descriptive analysis - part 3. **(A)**: using ensemble prioritization, **(B)**: AUC, **(C)**: overall accuracy.

**Figure 5:**
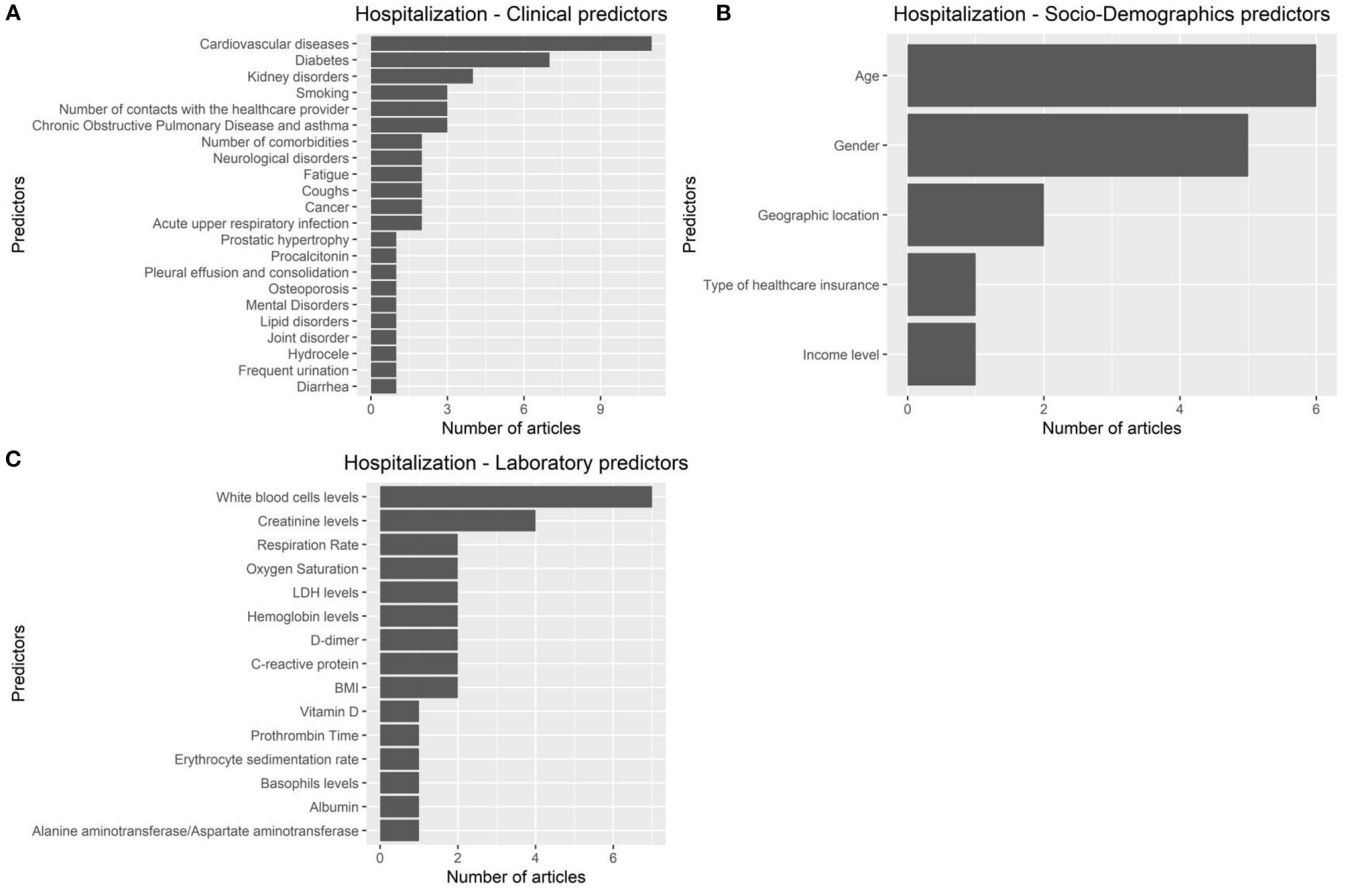
Predictors of COVID 19-related hospitalization. **(A)**: clinical predictors. **(B)**: socio-demohraphics predictrs. **(C)**: laboratory predictors.

**Figure 6:**
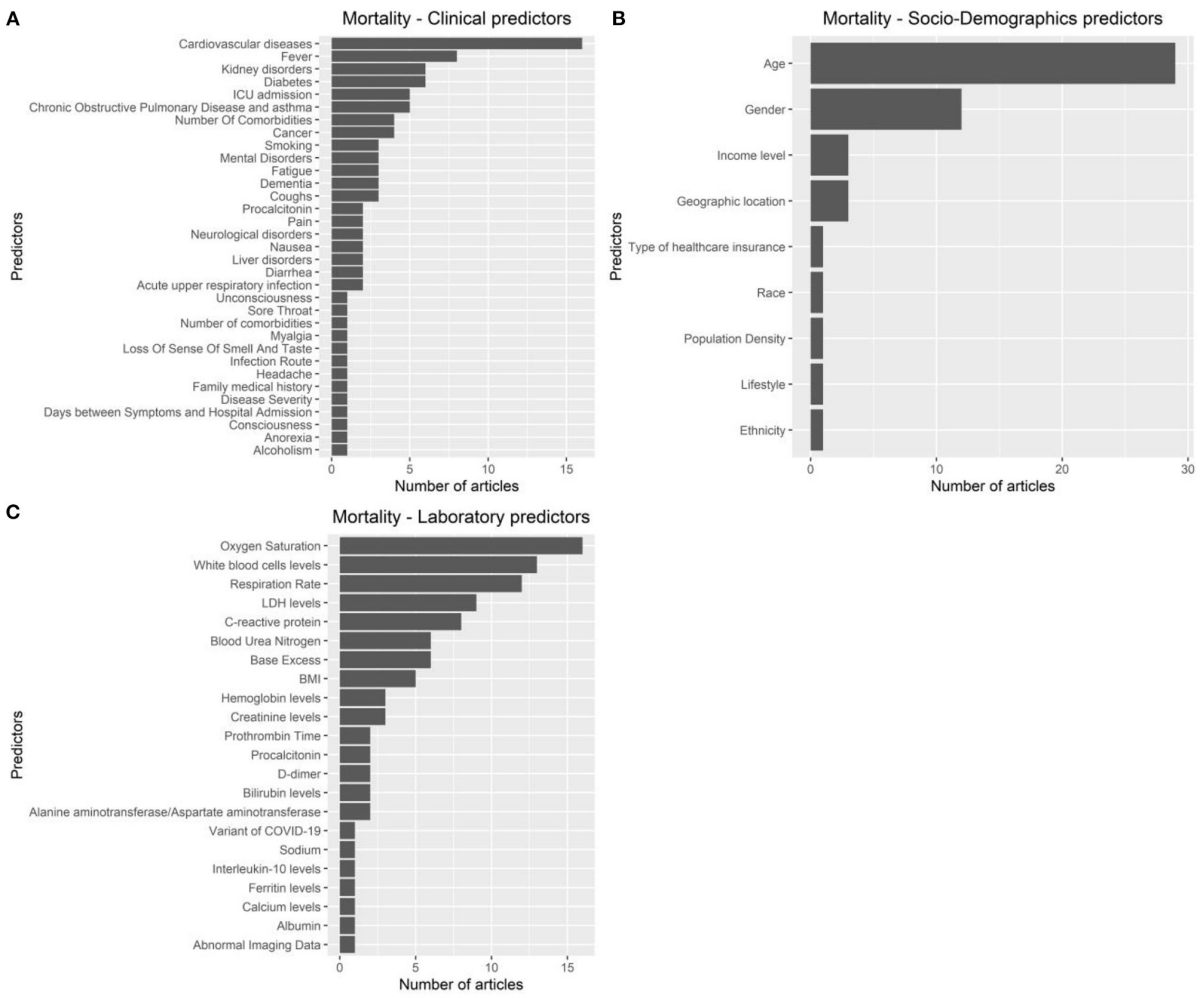
Predictors of COVID-19-related mortality. **(A)**: clinical predictors. **(B)**: socio-demohraphics predictrs. **(C)**: laboratory predictors.

The articles were published in the period 2019–2022, and mostly used Random Forest (Figures 2A, B). In most studies, the models were internally validated with cross-validation, but the majority of studies lacked external validation and calibration (Figures 2C, D, 3). Covariates were not prioritized using ensemble approaches in most of the studies, however, models still showed moderately good performances with Area under the Receiver operating characteristic Curve (AUC) values >0.7 (Figure 4). An overview of the predictors for COVID-19-related hospitalization and death is provided in Figures 5, 6.

### 3.2. Risk of bias and applicability

For all articles included in the systematic review, we performed a bias and applicability analysis using PROBAST (Appendices 1–38). Additionally, we have provided a narrative description and a summary of the key considerations we included in the bias analysis leading to the bias scoring (Appendix 39). Table 2 provides the results of PROBAST to evaluate the risk of bias and the applicability and quality of the AI models described in the retrieved articles. According to the assessment with PROBAST, all models had either high risk of bias or high concerns related to applicability.

**Table 2:** Tabular presentation for PROBAST results^*^.

		**ROB**				**Applicability**			**Overall**	
**Study**	**Outcome**	**Participants**	**Predictors**	**Outcomes**	**Analysis**	**Participants**	**Predictors**	**Outcomes**	**Bias**	**Applicability**
Polilli E 2022 (29)	Hospitalization and mortality	-	+	-	-	+	+	-	-	-
Wanyan T 2022 (30)	Mortality	-	+	-	-	+	-	-	-	-
Lazzarini N 2022 (31)	Hospitalization	+	+	-	-	+	-	-	-	-
Wang A 2022 (32)	Hospitalization	+	+	-	-	+	-	-	-	-
Vezzoli M 2022 (33)	Mortality	+	+	-	+	+	-	-	-	-
Ali S 2022 (34)	Mortality	-	-	-	+	+	-	-	-	-
Zarei J 2022 (35)	Mortality	+	+	+	+	+	+	+	?	+
Baik SM 2022 (36)	Mortality	-	-	-	-	+	-	-	-	-
Shanbehzadeh M 2022 (37)	Hospitalization	+	+	-	-	+	-	-	-	-
Song W 2022 (38)	Hospitalization	+	+	-	-	+	+	-	-	-
Willette AA 2022 (39)	Hospitalization	+	+	-	-	+	+	-	-	-
Wan TK 2022 (40)	Mortality	?	+	-	-	+	?	-	-	-
Park MS 2022 (41)	Mortality	-	+	?	-	-	?	?	?	+
Jakob CEM 2022 (42)	Hospitalization	?	+	?	-	+	+	?	-	-
Hernández-Pereira E 2022 (43)	Hospitalization	?	-	-	-	?	-	-	-	-
Gutierrez JM 2021 (44)	Hospitalization	+	+	+	-	+	?	+	?	-
Guan X 2021 (45)	Mortality	-	+	-	-	?	-	-	-	-
Feng C 2021 (46)	Mortality	+	-	?	-	+	-	?	-	-
Kasturi SN 2021 (47)	Hospitalization	+	+	+	-	?	?	+	+	-
Murri R 2021 (48)	Mortality	?	-	-	-	+	-	-	-	-
Tabatabaie M 2021 (49)	Mortality	?	-	-	-	+	-	-	-	-
Moulaei K 2021 (50)	Mortality	?	-	-	-	+	-	-	-	-
Migriño JR Jr 2021 (51)	Mortality	-	-	-	-	-	-	-	-	-
Banoei MM 2021 (52)	Mortality	?	+	+	-	+	+	+	-	-
Dabbah MA 2021 (53)	Mortality	+	?	?	-	+	?	-	-	-
De Souza FSH 2021 (54)	Mortality	+	-	?	-	+	-	-	-	-
Ottenhoff MC 2021 (55)	Mortality	+	+	?	-	+	-	-	?	-
Mahdavi M 2021 (56)	Mortality	+	-	+	-	+	-	-	-	-
Jamshidi E 2021 (57)	Mortality	-	+	?	-	-	-	-	-	-
Snider B 2021 (58)	Mortality	-	+	?	-	-	-	-	-	-
Halasz G 2021 (59)	Mortality	+	+	?	-	+	-	-	-	-
Karthikeyan A 2021 (60)	Mortality	+	-	?	-	+	-	-	-	-
Tezza F 2021 (61)	Mortality	?	-	?	-	+	-	-	-	-
Pourhomayoun M 2021 (62)	Mortality	?	+	?	+	+	?	?	?	?
Jimenez-Solem E 2021 (63)	Hospitalization and mortality	?	?	?	?	?	-	-	?	-
Gao Y 2020 (64)	Mortality	?	?	?	-	?	-	-	-	-
Wang T 2020 (65)	Mortality	+	?	?	-	-	-	-	-	-
An C 2020 (66)	Mortality	+	?	?	+	?	?	-	?	-
Vaid A 2020 (67)	Hospitalization and mortality	+	?	?	?	-	-	-	?	-

PROBAST, Prediction model Risk Of Bias ASsessment Tool; ROB, risk of bias.

^*^+ indicates low ROB/low concern regarding applicability; – indicates high ROB/high concern regarding applicability; and ? indicates unclear ROB/unclear concern regarding applicability.

## 4. Discussion

COVID-19 is the first pandemic that occurred in a digitized world and has sparked an unprecedented global research effort (8). With computers being ubiquitously used in modern societies, they are also expected to constitute a novel tool to fight global health emergencies. Therefore, it is not unexpected to observe an extensive application of AI to try solving key prediction tasks for important clinical outcomes such as COVID-19-related hospitalization and mortality.

The COVID-19 pandemic has stimulated considerable research efforts worldwide, but research output on this topic varies among countries as observed in our systematic review in the context of the use of AI for prediction tasks for important clinical outcomes such as COVID-19-related hospitalization and mortality. This heterogeneity can be attributed to several factors, including financial resources, research infrastructure, government support, outbreak severity, and collaboration and partnerships. Economic strength enables countries to invest more in research, and established universities and research institutions are better equipped to conduct COVID-19-related research. Government funding and policies that foster research are also essential for research development. Furthermore, countries that were hit hard by the pandemic may have prioritized research to combat the virus, and collaborations with other countries and international organizations may have increased research output (68–71).

In all, 27 studies used AI to predict COVID-19 mortality, 9 studies used AI to predict COVID-19-related hospitalization, and 3 studies had both outcomes. The articles focusing on hospitalization typically used patient demographics, medical history, vital signs, and laboratory results as input variables for their machine learning models. The primary goal of these studies was to identify high-risk patients early on, so that appropriate medical interventions can be initiated promptly. Similarly, the articles focusing on mortality used similar input variables, but with additional focus on disease severity and progression. The main objective of these studies was to detect patients with a high likelihood of mortality, allowing them to receive close monitoring and more intensive care. The articles that predicted both hospitalization and mortality outcomes using machine learning models utilize the same input variables as those concentrating only on hospitalization or mortality. However, they also take into account the interplay between these outcomes. Overall, the key differences between these article groups lie in their primary outcome of interest and the input variables used in their machine learning models.

In the articles retrieved in our systematic review, several machine learning models have been used to predict COVID-19-related hospitalization and mortality, with Random Forest being the model most frequently used. It cannot be excluded that this result is due to publication bias. However, the potential analytical advantage of using Random Forest in terms of prediction accuracy should be acknowledged as Random Forest undoubtedly represents an important and widely used tool for prediction in medical research (72).

While it is possible that research teams in certain countries may have a preference for using certain models or methods, such as Random Forest models, in their research, it is unlikely to be the primary driver for our findings. Research on COVID-19 encompasses various disciplines that require different models and methods. Therefore, the choice of models and methods used in research is usually based on their relevance to the research question at hand, rather than personal preference or bias. However, in the retrieved article, we have not seen any argumentation for why applied studies choose to use particular models, and the authors often seem to use only a few (seemingly arbitrarily selected) ones, and even so, the authors often use some of these methods only with default parameters (which are again arbitrary), instead of motivating hyper-parameter choices by the application or selecting their values by a systematic hyper-parameter search.

AI models were trained using cohorts of a median sample size of 4,000 individuals (the smallest sample was 165 individuals) collecting information on demographics, clinical records, laboratory results, and pharmacological Studies had a median of 53 covariates, with only 3 studies having more than 1,200 covariates. This is not surprising considering that most studies were hospital-based and collected information on individuals admitted to wards from hospital databases.

In most studies, the models were internally validated with cross-validation, but the majority of studies lacked external validation and calibration. External validation is a crucial activity when using AI in prediction modelling. In absence of external validation, it is often not possible to determine a prediction model's reproducibility and generalizability to new and different sets of data generated in different settings and/or different time points of a specific setting (e.g., different COVID-19 waves) (73).

Calibration is important, albeit often overlooked, aspect of training AI models. It's important to recognize the fact that calibration directly modifies the outputs of machine learning models after they have been trained and can have an impact on the accuracy of the model. When assessing a model's validity, calibration is as important as other performance metrics and should be evaluated and reported. Model calibration refers to the agreement between subgroups of predicted probabilities and their observed frequencies. To assess model calibration, a calibration plot can be generated by ordering the predicted probabilities, dividing them into subgroups, and then plotting the average predicted probability vs. the average outcome for each subgroup (22).

In most of the studies, however, models still showed moderately good performances with AUC values >0.7. It cannot be excluded that this result is due to publication bias.

According to the assessment with PROBAST, models had high risk of bias and/or poor applicability. Commonly identified biases included condition on a future event, misclassification of exposure/outcome, and or selection bias. The main concern we had in retrieved studies regarding the applicability of the models is related to their description of the study aims and their target population. Articles for which we identified concerns related to applicability did not confine the generalizability of their models to settings in which the models were developed but rather had fairly general claims that their model could generalize in other settings. When describing the area of applicability of an AI model it is crucial to set boundaries of which settings and necessary conditions will guarantee the applicability of the model. When this information is missing in the manuscript, applicability can be considered global and, therefore, the study prone to selection bias. This is especially true for hospital-based studies, as emphasized by Kopec and Grimes (74, 75).

## 5. Conclusion

A broad range of AI techniques have been used to predict COVID-19 hospitalization and mortality. The studies reported good prediction performance for the AI Models. However, according to the assessment with PROBAST, all models had a high risk of bias and/or concern regarding applicability.

## Data availability statement

The original contributions presented in the study are included in the article/Supplementary material, further inquiries can be directed to the corresponding author.

## Author contributions

MS and SS conceived of the presented idea. SS developed the theory and performed the computations. All authors discussed the results and contributed to the final manuscript.
