# Supplementary material for: Artificial intelligence-driven prediction of COVID-19-related hospitalization and death: a systematic review
Source: Front Public Health. 2023 Jun 20;11:1183725. doi: 10.3389/fpubh.2023.1183725 (PMC10319067; doi:10.3389/fpubh.2023.1183725)
Supplement: Supplementary file 2 [file Data_Sheet_2.DOCX]

**APPENDICIES**

**Appendix 0**. Lazzarini 2022 - Bias analysis using PROBAST.

**Step 1: Specify your systematic review question**

| **Criteria** | **Specify your systematic review question** |
| --- | --- |
| *Intended use of model:* | To predict severe cases of COVID-19 patients defined as the presence of Acute Respiratory Distress Syndrome (ARDS) and highlight the different risk factors that play a significant role in disease progression. |
| ***Participants*** *including selection criteria and setting:* | Patients positive to COVID-19. |
| ***Predictors*** *(used in prediction modelling), including types of predictors (e.g. history, clinical examination, biochemical markers, imaging tests), time of measurement, specific measurement issues (e.g., any requirements/ prohibitions for specialized equipment):* | Demographics, Medical, Clinical |
| *Outcome to be predicted:* | Severity of COVID-19 infection. (Severity has been defined as inpatient hospitalization by the work of Kenneth et al) |

**Step 2: Classify the type of prediction model evaluation**

| **Classify the evaluation based on its aim** | | | |
| --- | --- | --- | --- |
| **Type of prediction study** | **PROBAST boxes to complete** | **Tick as appropriate** | **Definition for type of prediction model study** |
| Development only | Development | ✔ | Prediction model development without external validation. These studies may include internal validation methods, such as bootstrapping and cross-validation techniques. |
| Development and validation | Development and validation | ✖ | Prediction model development combined with external validation in other participants in the same article. |
| Validation only | Validation | ✖ | External validation of existing (previously developed) model in other participants. |

|  | |
| --- | --- |
| **Publication reference** | Lazzarini N, Filippoupolitis A, Manzione P, Eleftherohorinou H. A machine learning model on Real World Data for predicting progression to Acute Respiratory Distress Syndrome (ARDS) among COVID-19 patients. PLoS One. 2022 Jul 28;17(7):e0271227. doi: 10.1371/journal.pone.0271227. PMID: 35901089; PMCID: PMC9333235. | |
| **Models of interest** | Logistic Regression, Random Forest and Gradient Boosting Decision Tree |  |
| **Outcome of interest** | Hospitalization | |

**Step 3: Assess risk of bias and applicability**

| **DOMAIN 1:  Participants** | | | |
| --- | --- | --- | --- |
| **A. Risk of Bias** | | | |
| *Describe the sources of data and criteria for participant selection:*  DEV:  A cohort composed of 289,351 patients diagnosed with COVID-19 in April 2020 was created using US administrative claims data from Oct 2015 to Jul 2020. For each patient, information about 817 diagnoses, were collected from the medical history ahead of COVID-19 infection. The primary outcome of the study was the presence of ARDS in the 4 months following COVID-19 infection.  Patients were included in the initial cohort if they were diagnosed for the first time with COVID-19 (ICD-10 code U07.1) in the month of April 2020. That is, they had no claims with ICD code U07.1 before April 2020. Furthermore, to be included in the initial cohort, the patients should have never been diagnosed with ARDS (ICD-10 code J80) between October 2015 and January 2020. A patient was then labelled as positive (case) if diagnosed with ARDS by the end of July 2020, otherwise the patient was labelled as negative (control). Patients with missing age or gender information were discarded from the analysis. In this study they decided to focus only on patients diagnosed in April 2020 because up to March 2020, the code U07.1 was used for any type of coronavirus infection, not specifically for COVID-19 | | | |
|  | | Dev | Val |
| 1. Were appropriate data sources used, e.g. cohort, RCT or nested case-control study data? | | Y |  |
| 1. Were all inclusions and exclusions of participants appropriate? | | PY |  |
| **Risk of bias introduced by selection of participants** | **RISK:**  *(low/ high/ unclear)* | **low** |  |
| *Rationale of bias rating: No concerns* | | | |
|  | | | |
| **B. Applicability** | | | |
| *Describe included participants, setting and dates:*  It has been mentioned in Domain 1 – Section A. | | | |
| **Concern that the included participants and setting do not match the review question** | **CONCERN:**  *(low/ high/ unclear)* | **low** |  |
| *Rationale of applicability rating:* | | | |
| In our review, our source population is the general population. Therefore, we did not restrict the source population to those diagnosed with ARDS. Consequently, their data with diagnosed with ARDS are part of the general population. | | | |

| **DOMAIN 2:  Predictors** | | | |
| --- | --- | --- | --- |
| **A. Risk of Bias** | | | |
| *List and describe predictors included in the final model, e.g. definition and timing of assessment:*  age, gender, Diabetes mellitus, Hypertensive Diseases and Obesity, acute upper respiratory infection, other joint disorder, vitamin D deficiency, Malaise and fatigue, and nicotine addiction | | | |
|  | | Dev | Val |
| 1. Were predictors defined and assessed in a similar way for all participants? | | Y |  |
| 1. Were predictor assessments made without knowledge of outcome data? | | Y |  |
| 1. Are all predictors available at the time the model is intended to be used? | | Y |  |
| **Risk of bias introduced by predictors or their assessment** | **RISK:**  *(low/ high/ unclear)* | **low** |  |
| *Rationale of bias rating: No concerns* | | | |
| **B. Applicability** | | | |
| Concern that the definition, assessment or timing of predictors in the model do not match the review question | **CONCERN:**  *(low/ high/ unclear)* | **high** |  |
| *Rationale of applicability rating:*  There is a great concern regarding variable generation. The value of each feature was set to 1 if the patient had a diagnosis for that disease within the lookback window, otherwise it was set to 0. The method ignores the frequency of a diagnosis for that disease. Moreover age and gender were also used as input features which are not in the same scale as 0 and 1 values. | | | |

| **DOMAIN 3: Outcome** | | | |
| --- | --- | --- | --- |
| **A. Risk of Bias** | | | |
| *Describe the outcome, how it was defined and determined, and the time interval between predictor assessment and outcome determination:*  Severity of COVID-19 patients through follow-up of all patients in the study population was investigated. | | | |
|  | | Dev | Val |
| 1. Was the outcome determined appropriately? | | PY |  |
| 1. Was a pre-specified or standard outcome definition used? | | PY |  |
| 1. Were predictors excluded from the outcome definition? | | Y |  |
| 1. Was the outcome defined and determined in a similar way for all participants? | | Y |  |
| 1. Was the outcome determined without knowledge of predictor information? | | PY |  |
| 1. Was the time interval between predictor assessment and outcome determination appropriate? | | NI |  |
| **Risk of bias introduced by the outcome or its determination** | **RISK:**  *(low/ high/ unclear)* | **high** |  |
| *Rationale of bias rating:*  They defined COVID-19 severity as the presence of ARDS within four months of the initial infection. There is no mentioned reference for the definition. | | | |
| **B. Applicability** | | | |
| *At what time point was the outcome determined:*  There is no reference for the definition of COVID-19 severity.  *If a composite outcome was used, describe the relative frequency/distribution of each contributing outcome:* | | | |
| **Concern that the outcome, its definition, timing or determination do not match the review question** | **CONCERN:**  *(low/ high/ unclear)* | **high** |  |
| *Rationale of applicability rating:*  There is concerns regarding the definition of COVID-19 severity. | | | |

| **DOMAIN 4: Analysis** | | | |
| --- | --- | --- | --- |
| **Risk of Bias** | | | |
| *Describe numbers of participants, number of candidate predictors, outcome events and events per candidate predictor:*  DEV: 289,351 participants, 817 boolean comorbidities features plus age and gender as input features  10,793 had developed severe COVID-19 | | | |
| *Describe how the model was developed (for example in regards to modelling technique (e.g. survival or logistic modelling), predictor selection, and risk group definition):* | | | |
| *Describe whether and how the model was validated, either internally (e.g. bootstrapping, cross validation, random split sample) or externally (e.g. temporal validation, geographical validation, different setting, different type of participants):*  Cross validation has not been used. | | | |
| *Describe the performance measures of the model, e.g. (re)calibration, discrimination, (re)classification, net benefit, and whether they were adjusted for optimism:*  A ROC analysis was performed using a separate 2 × 2 “confusion matrix” for every possible threshold applied to the total score. The best threshold was defined by the highest value of the Youden Index. Point estimates and confidence intervals were computed using the bootstrap for sensitivity, specificity, Positive Predictive Value (PPV), Negative Predictive Value (NPV) and the De Long method for the Area Under the Curve (AUC)  Reliability analysis of the predictive formula was summarized using ROC curves showing optimal thresholds and sensitivity, specificity, AUC with point estimates and 95% confidence intervals. | | | |
| *Describe any participants who were excluded from the analysis:*  Not available. | | | |
| *Describe missing data on predictors and outcomes as well as methods used for missing data:*  Not available. | | | |
|  | | Dev | Val |
| 1. Were there a reasonable number of participants with the outcome? | | NI |  |
| 1. Were continuous and categorical predictors handled appropriately? | | PY |  |
| 1. Were all enrolled participants included in the analysis? | | PY |  |
| 1. Were participants with missing data handled appropriately? | | NI |  |
| 1. Was selection of predictors based on univariable analysis avoided? | | Y |  |
| 1. Were complexities in the data (e.g. censoring, competing risks, sampling of controls) accounted for appropriately? | | Y |  |
| 1. Were relevant model performance measures evaluated appropriately? | | Y |  |
| 1. Were model overfitting and optimism in model performance accounted for? | | N |  |
| 1. Do predictors and their assigned weights in the final model correspond to the results from multivariable analysis? | | NI |  |
| **Risk of bias introduced by the analysis** | **RISK:**  *(low/ high/ unclear)* | **high** |  |
| *Rationale of bias rating:*  The value of each feature was set to 1 and 0. The method ignores the frequency of a code for each patient during the assessment window. Moreover age and gender were also used as input features which are not in the same scale as 0 and 1 values.  *They did not perform cross validation.*  They did not describe how they handled missing data. | | | |

**Step 4: Overall assessment**

| | **Reaching an overall judgement about risk of bias of the prediction model evaluation** | | | --- | --- | | **Low risk of bias** | If all domains were rated low risk of bias.  If a prediction model was developed without any external validation, and it was rated as low risk of bias for all domains, consider downgrading to **high risk of bias**. Such a model can only be considered as low risk of bias, if the development was based on a very large data set and included some form of internal validation. | | **High risk of bias** | If at least one domain is judged to be at **high risk of bias**. | | **Unclear risk of bias** | If an unclear risk of bias was noted in at least one domain and it was low risk for all other domains. |  | **Reaching an overall judgement about applicability of the prediction model evaluation** | | | --- | --- | | **Low concerns regarding applicability** | If low concerns regarding applicability for all domains, the prediction model evaluation is judged to have **low concerns regarding applicability**. | | **High concerns regarding applicability** | If high concerns regarding applicability for at least one domain, the prediction model evaluation is judged to have **high concerns regarding applicability**. | | **Unclear concerns regarding applicability** | If unclear concerns (but no “high concern”) regarding applicability for at least one domain, the prediction model evaluation is judged to have **unclear concerns regarding applicability** overall. | |
| --- | --- | --- | --- | --- | --- | --- | --- | --- | --- | --- | --- | --- | --- | --- | --- | --- |

| **Overall judgement about risk of bias and applicability of the prediction model evaluation** | | |
| --- | --- | --- |
| **Overall judgement of risk of bias** | **RISK:**  *(low/ high/ unclear)* | **high** |
| *Summary of sources of potential bias:*  The value of each feature was set to 1 and 0. The method ignores the frequency of a code for each patient during the assessment window. Moreover age and gender were also used as input features which are not in the same scale as 0 and 1 values.  *They did not perform cross validation.*  They did not describe how they handled missing data. | | |
| **Overall judgement of applicability** | **CONCERN:**  *(low/ high/ unclear)* | **high** |
| *Summary of applicability concerns:*  There is no reference for the definition of COVID-19 severity.  There is a great concern regarding variable generation. The value of each feature was set to 1 if the patient had a diagnosis for that disease within the lookback window, otherwise it was set to 0. The method ignores the frequency of a diagnosis for that disease. Moreover age and gender were also used as input features which are not in the same scale as 0 and 1 values. | | |

**Appendix 1**. Shanbehzadeh 2022 - Bias analysis using PROBAST.

**Step 1: Specify your systematic review question**

| **Criteria** | **Specify your systematic review question** |
| --- | --- |
| *Intended use of model:* | It aims to predict the likelihood of COVID-19 readmission risk by employing machine learning. |
| ***Participants*** *including selection criteria and setting:* | Discharged COVID-19 patients |
| ***Predictors*** *(used in prediction modelling), including types of predictors (e.g. history, clinical examination, biochemical markers, imaging tests), time of measurement, specific measurement issues (e.g., any requirements/ prohibitions for specialized equipment):* | Demographic, diagnostic and therapeutic, paraclinical, and history and information. |
| *Outcome to be predicted:* | Readmission of COVID-19 infection. |

**Step 2: Classify the type of prediction model evaluation**

| **Classify the evaluation based on its aim** | | | |
| --- | --- | --- | --- |
| **Type of prediction study** | **PROBAST boxes to complete** | **Tick as appropriate** | **Definition for type of prediction model study** |
| Development only | Development | ✔ | Prediction model development without external validation. These studies may include internal validation methods, such as bootstrapping and cross-validation techniques. |
| Development and validation | Development and validation | ✖ | Prediction model development combined with external validation in other participants in the same article. |
| Validation only | Validation | ✖ | External validation of existing (previously developed) model in other participants. |

|  | |
| --- | --- |
| **Publication reference** | Shanbehzadeh M, Yazdani A, Shafiee M, Kazemi-Arpanahi H. Predictive modeling for COVID-19 readmission risk using machine learning algorithms. BMC Med Inform Decis Mak. 2022 May 20;22(1):139. doi: 10.1186/s12911-022-01880-z. PMID: 35596167; PMCID: PMC9122247. | |
| **Models of interest** | Decision tree, support vector machine, and k-nearest neighbors, and a hybrid algorithm, namely water wave optimization (WWO) as a precise metaheuristic evolutionary algorithm |  |
| **Outcome of interest** | Hospitalization | |

**Step 3: Assess risk of bias and applicability**

| **DOMAIN 1:  Participants** | | | |
| --- | --- | --- | --- |
| **A. Risk of Bias** | | | |
| *Describe the sources of data and criteria for participant selection:*  DEV:  Te current research was a retrospective study on the data of 2854 patients discharged from a 400-bed academic hospital in Abadan, Iran, from January 9, 2020 to October 20, 2021. The patient data were extracted from the COVID-19 hospital-based registry database. | | | |
|  | | Dev | Val |
| 1. Were appropriate data sources used, e.g. cohort, RCT or nested case-control study data? | | Y |  |
| 1. Were all inclusions and exclusions of participants appropriate? | | PY |  |
| **Risk of bias introduced by selection of participants** | **RISK:**  *(low/ high/ unclear)* | **low** |  |
| *Rationale of bias rating: No Concerns* | | | |
|  | | | |
| **B. Applicability** | | | |
| *Describe included participants, setting and dates:*  It has been mentioned in Domain 1 – Section A. | | | |
| **Concern that the included participants and setting do not match the review question** | **CONCERN:**  *(low/ high/ unclear)* | **low** |  |
| *Rationale of applicability rating:* | | | |
| In our review, our source population is the general population. Therefore, this data is part of the general population. | | | |

| **DOMAIN 2:  Predictors** | | | |
| --- | --- | --- | --- |
| **A. Risk of Bias** | | | |
| *List and describe predictors included in the final model, e.g. definition and timing of assessment:*  Coughs, cardiovascular disease, diabetes, hypertension, prior oxygen therapy, CRP, creatinine, ESR, D-dimer, ALT/ASP, absolute lymphocyte/ neutrophil count, pleural efusion and consolidation. | | | |
|  | | Dev | Val |
| 1. Were predictors defined and assessed in a similar way for all participants? | | PY |  |
| 1. Were predictor assessments made without knowledge of outcome data? | | PY |  |
| 1. Are all predictors available at the time the model is intended to be used? | | Y |  |
| **Risk of bias introduced by predictors or their assessment** | **RISK:**  *(low/ high/ unclear)* | **low** |  |
| *Rationale of bias rating:*  *No Concerns* | | | |
| **B. Applicability** | | | |
| Concern that the definition, assessment or timing of predictors in the model do not match the review question | **CONCERN:**  *(low/ high/ unclear)* | **High** |  |
| *Rationale of applicability rating:*  *The timing of predictors in the model were not defined.* | | | |

| **DOMAIN 3: Outcome** | | | |
| --- | --- | --- | --- |
| **A. Risk of Bias** | | | |
| *Describe the outcome, how it was defined and determined, and the time interval between predictor assessment and outcome determination:*  It calculated whether the patient was readmitted on the last visit within 30 days after being discharged from the hospital on the penultimate visit | | | |
|  | | Dev | Val |
| 1. Was the outcome determined appropriately? | | PN |  |
| 1. Was a pre-specified or standard outcome definition used? | | PN |  |
| 1. Were predictors excluded from the outcome definition? | | Y |  |
| 1. Was the outcome defined and determined in a similar way for all participants? | | PY |  |
| 1. Was the outcome determined without knowledge of predictor information? | | PY |  |
| 1. Was the time interval between predictor assessment and outcome determination appropriate? | | NI |  |
| **Risk of bias introduced by the outcome or its determination** | **RISK:**  *(low/ high/ unclear)* | **high** |  |
| *Rationale of bias rating:*  It is not clear how the interval time between readmission and discharging (30 days) was defined. There is no mentioned reference for the definition. | | | |
| **B. Applicability** | | | |
| *At what time point was the outcome determined:*  It calculated whether the patient was readmitted on the last visit within 30 days after being discharged from the hospital on the penultimate visit  *If a composite outcome was used, describe the relative frequency/distribution of each contributing outcome:* | | | |
| **Concern that the outcome, its definition, timing or determination do not match the review question** | **CONCERN:**  *(low/ high/ unclear)* | **high** |  |
| *Rationale of applicability rating:*  Choosing the 30 days may not be appropriate and can cause different results in applying the model in other settings. | | | |
| **DOMAIN 4: Analysis** | | | |
| **Risk of Bias** | | | |
| *Describe numbers of participants, number of candidate predictors, outcome events and events per candidate predictor:*  DEV: non-readmissions:1136, readmissions: 89 cases | | | |
| *Describe how the model was developed (for example in regards to modelling technique (e.g. survival or logistic modelling), predictor selection, and risk group definition):*  Decision tree, support vector machine, and k-nearest neighbors, and a hybrid algorithm, namely water wave optimization (WWO) as a precise metaheuristic evolutionary algorithm | | | |
| *Describe whether and how the model was validated, either internally (e.g. bootstrapping, cross validation, random split sample) or externally (e.g. temporal validation, geographical validation, different setting, different type of participants):*  Cross validation was used. | | | |
| *Describe the performance measures of the model, e.g. (re)calibration, discrimination, (re)classification, net benefit, and whether they were adjusted for optimism:*  The ROC analysis was performed. | | | |
| *Describe any participants who were excluded from the analysis:*  Patients aged less than 18  years, those who were admitted for non-COVID-19 conditions, died during hospitalization, were discharged against medical advice, or had incomplete case records with>70% missing data were excluded from the study | | | |
| *Describe missing data on predictors and outcomes as well as methods used for missing data:*  Records with more than 70% of missing data were excluded from the analysis. For the remaining missing values, presuming that the missing data were distributed randomly. | | | |
|  | | Dev | Val |
| 1. Were there a reasonable number of participants with the outcome? | | N |  |
| 1. Were continuous and categorical predictors handled appropriately? | | NI |  |
| 1. Were all enrolled participants included in the analysis? | | PY |  |
| 1. Were participants with missing data handled appropriately? | | PY |  |
| 1. Was selection of predictors based on univariable analysis avoided? | | NI |  |
| 1. Were complexities in the data (e.g. censoring, competing risks, sampling of controls) accounted for appropriately? | | PY |  |
| 1. Were relevant model performance measures evaluated appropriately? | | Y |  |
| 1. Were model overfitting and optimism in model performance accounted for? | | PY |  |
| 1. Do predictors and their assigned weights in the final model correspond to the results from multivariable analysis? | | PY |  |
| **Risk of bias introduced by the analysis** | **RISK:**  *(low/ high/ unclear)* | **high** |  |
| *Rationale of bias rating:*  There was not enough data to evaluate appropriately the model used for the prediction.  This study did not describe any causal relationship between the predictor and outcome variables.  The analyses were based on a single-center dataset, therefore the results of this study may not be generalizable. | | | |

**Step 4: Overall assessment**

| | **Reaching an overall judgement about risk of bias of the prediction model evaluation** | | | --- | --- | | **Low risk of bias** | If all domains were rated low risk of bias.  If a prediction model was developed without any external validation, and it was rated as low risk of bias for all domains, consider downgrading to **high risk of bias**. Such a model can only be considered as low risk of bias, if the development was based on a very large data set and included some form of internal validation. | | **High risk of bias** | If at least one domain is judged to be at **high risk of bias**. | | **Unclear risk of bias** | If an unclear risk of bias was noted in at least one domain and it was low risk for all other domains. |  | **Reaching an overall judgement about applicability of the prediction model evaluation** | | | --- | --- | | **Low concerns regarding applicability** | If low concerns regarding applicability for all domains, the prediction model evaluation is judged to have **low concerns regarding applicability**. | | **High concerns regarding applicability** | If high concerns regarding applicability for at least one domain, the prediction model evaluation is judged to have **high concerns regarding applicability**. | | **Unclear concerns regarding applicability** | If unclear concerns (but no “high concern”) regarding applicability for at least one domain, the prediction model evaluation is judged to have **unclear concerns regarding applicability** overall. | |
| --- | --- | --- | --- | --- | --- | --- | --- | --- | --- | --- | --- | --- | --- | --- | --- | --- |

| **Overall judgement about risk of bias and applicability of the prediction model evaluation** | | |
| --- | --- | --- |
| **Overall judgement of risk of bias** | **RISK:**  *(low/ high/ unclear)* | **high** |
| *Summary of sources of potential bias:*  There was not enough data to evaluate appropriately the model used for the prediction.  This study did not describe any causal relationship between the predictor and outcome variables. | | |
| **Overall judgement of applicability** | **CONCERN:**  *(low/ high/ unclear)* | **high** |
| *Summary of applicability concerns:*  It calculated whether the patient was readmitted on the last visit within 30 days after being discharged from the hospital on the penultimate visit. Choosing the 30 days may not be appropriate and can cause different results in applying the model in other settings.  The analyses were based on a single-center dataset, therefore the results of this study may not be generalizable. | | |

**Appendix 2**. Song 2022 - Bias analysis using PROBAST.

**Step 1: Specify your systematic review question**

| **Criteria** | **Specify your systematic review question** |
| --- | --- |
| *Intended use of model:* | To predict the hospitalization of older adults who have tested positive for COVID-19. |
| ***Participants*** *including selection criteria and setting:* | Positive tested COVID-19 older adults |
| ***Predictors*** *(used in prediction modelling), including types of predictors (e.g. history, clinical examination, biochemical markers, imaging tests), time of measurement, specific measurement issues (e.g., any requirements/ prohibitions for specialized equipment):* | Demographics, diagnoses, procedures, medications, laboratory tests |
| *Outcome to be predicted:* | Hospitaliation |

**Step 2: Classify the type of prediction model evaluation**

| **Classify the evaluation based on its aim** | | | |
| --- | --- | --- | --- |
| **Type of prediction study** | **PROBAST boxes to complete** | **Tick as appropriate** | **Definition for type of prediction model study** |
| Development only | Development | ✔ | Prediction model development without external validation. These studies may include internal validation methods, such as bootstrapping and cross-validation techniques. |
| Development and validation | Development and validation | ✖ | Prediction model development combined with external validation in other participants in the same article. |
| Validation only | Validation | ✖ | External validation of existing (previously developed) model in other participants. |

|  | |
| --- | --- |
| **Publication reference** | Song W, Zhang L, Liu L, Sainlaire M, Karvar M, Kang MJ, Pullman A, Lipsitz S, Massaro A, Patil N, Jasuja R, Dykes PC. Predicting hospitalization of COVID-19 positive patients using clinician-guided machine learning methods. J Am Med Inform Assoc. 2022 Sep 12;29(10):1661-1667. doi: 10.1093/jamia/ocac083. PMID: 35595237; PMCID: PMC9129151. | |
| **Models of interest** | Regularized logistic regression, support vector machine, random forest, and neural network |  |
| **Outcome of interest** | Hospitalization | |

**Step 3: Assess risk of bias and applicability**

| **DOMAIN 1:  Participants** | | | |
| --- | --- | --- | --- |
| **A. Risk of Bias** | | | |
| *Describe the sources of data and criteria for participant selection:*  DEV:  It used clinical databases within the MGB Healthcare system, which has a centralized clinical data warehouse for all types of clinical information from multiple Harvard-affiliated hospitals. Dataset included patient demographics, diagnoses, procedures, medications, laboratory tests, inpatient and outpatient encounter information, and provider data. Clinical data from 11 MGB hospitals were also included. | | | |
|  | | Dev | Val |
| 1. Were appropriate data sources used, e.g. cohort, RCT or nested case-control study data? | | Y |  |
| 1. Were all inclusions and exclusions of participants appropriate? | | PY |  |
| **Risk of bias introduced by selection of participants** | **RISK:**  *(low/ high/ unclear)* | **low** |  |
| *Rationale of bias rating: No Concerns* | | | |
|  | | | |
| **B. Applicability** | | | |
| *Describe included participants, setting and dates:*  It has been mentioned in Domain 1 – Section A. | | | |
| **Concern that the included participants and setting do not match the review question** | **CONCERN:**  *(low/ high/ unclear)* | **low** |  |
| *Rationale of applicability rating:* | | | |
| In our review, our source population is the general population. Therefore, this data is part of the general population. | | | |

| **DOMAIN 2:  Predictors** | | | |
| --- | --- | --- | --- |
| **A. Risk of Bias** | | | |
| *List and describe predictors included in the final model, e.g. definition and timing of assessment:*  Age, Gender, BMI, Smoking Status, SPO2, Temperature, Diabetes, Alzheimer Disease, Cancer, Cardiomyopathy, Cerebrovascular Disease, Chronic Kidney Disease, Chronic Respiratory Disease, Coronary Artery Disease, Cystic Fibrosis, Dementia, Dyslipidemia, Heart Failure, HIV/AIDS, Hypertensive Disease, Immunodeficiency, Liver Disease, Metastatic Solid Tumor, Sickle Cell Disease, Solid Organ Transplant, Albumin, White Blood Count, Blood Urea Nitrogen, Lymphocyte Count | | | |
|  | | Dev | Val |
| 1. Were predictors defined and assessed in a similar way for all participants? | | PY |  |
| 1. Were predictor assessments made without knowledge of outcome data? | | PY |  |
| 1. Are all predictors available at the time the model is intended to be used? | | Y |  |
| **Risk of bias introduced by predictors or their assessment** | **RISK:**  *(low/ high/ unclear)* | **low** |  |
| *Rationale of bias rating:* | | | |
| **B. Applicability** | | | |
| Concern that the definition, assessment or timing of predictors in the model do not match the review question | **CONCERN:**  *(low/ high/ unclear)* | **Low** |  |
| *Rationale of applicability rating: No Concerns* | | | |

| **DOMAIN 3: Outcome** | | | |
| --- | --- | --- | --- |
| **A. Risk of Bias** | | | |
| *Describe the outcome, how it was defined and determined, and the time interval between predictor assessment and outcome determination:*  They extracted both model outcome (hospitalization) and time-sensitive input features (lab values) from the 4-week time window surrounding the COVID test date. | | | |
|  | | Dev | Val |
| 1. Was the outcome determined appropriately? | | PY |  |
| 1. Was a pre-specified or standard outcome definition used? | | PY |  |
| 1. Were predictors excluded from the outcome definition? | | Y |  |
| 1. Was the outcome defined and determined in a similar way for all participants? | | PY |  |
| 1. Was the outcome determined without knowledge of predictor information? | | PY |  |
| 1. Was the time interval between predictor assessment and outcome determination appropriate? | | NI |  |
| **Risk of bias introduced by the outcome or its determination** | **RISK:**  *(low/ high/ unclear)* | **high** |  |
| *Rationale of bias rating:*  They extracted both model outcome (hospitalization) and time-sensitive input features (lab values) from the 4-week time window surrounding the COVID test date, leading to the possibility the outcome might precede the inputs. | | | |
| **B. Applicability** | | | |
| *At what time point was the outcome determined:*  *If a composite outcome was used, describe the relative frequency/distribution of each contributing outcome:* | | | |
| **Concern that the outcome, its definition, timing or determination do not match the review question** | **CONCERN:**  *(low/ high/ unclear)* | **high** |  |
| *Rationale of applicability rating:*  4-week could bias the relationship between input and outcome in The model and limit its prediction ability. | | | |
| **DOMAIN 4: Analysis** | | | |
| **Risk of Bias** | | | |
| *Describe numbers of participants, number of candidate predictors, outcome events and events per candidate predictor:*  DEV: patients with age 65 and above from the outpatient setting :1495, Hospitalized: 495cases | | | |
| *Describe how the model was developed (for example in regards to modelling technique (e.g. survival or logistic modelling), predictor selection, and risk group definition):*  Regularized logistic regression, support vector machine, random forest, and neural network | | | |
| *Describe whether and how the model was validated, either internally (e.g. bootstrapping, cross validation, random split sample) or externally (e.g. temporal validation, geographical validation, different setting, different type of participants):*  Cross validation was used. | | | |
| *Describe the performance measures of the model, e.g. (re)calibration, discrimination, (re)classification, net benefit, and whether they were adjusted for optimism:*  The ROC analysis was performed. | | | |
| *Describe any participants who were excluded from the analysis:*  Inpatients, patients aged below 65, patients with high missing values and low data quality were removed | | | |
| *Describe missing data on predictors and outcomes as well as methods used for missing data:*  No information was found how to handle missing values | | | |
|  | | Dev | Val |
| 1. Were there a reasonable number of participants with the outcome? | | Y |  |
| 1. Were continuous and categorical predictors handled appropriately? | | NI |  |
| 1. Were all enrolled participants included in the analysis? | | PY |  |
| 1. Were participants with missing data handled appropriately? | | NI |  |
| 1. Was selection of predictors based on univariable analysis avoided? | | NI |  |
| 1. Were complexities in the data (e.g. censoring, competing risks, sampling of controls) accounted for appropriately? | | PY |  |
| 1. Were relevant model performance measures evaluated appropriately? | | Y |  |
| 1. Were model overfitting and optimism in model performance accounted for? | | PY |  |
| 1. Do predictors and their assigned weights in the final model correspond to the results from multivariable analysis? | | PY |  |
| **Risk of bias introduced by the analysis** | **RISK:**  *(low/ high/ unclear)* | **high** |  |
| *Rationale of bias rating:*  Many features in the initial feature list have high missing rates for outpatients, for example, Medication records (eg, hospitalized patients had more complete records). Therefore, they did not include medications and other data types with high missingness in the model. They also removed significant number of patients with high missing values to develop the final study cohort, which allows them to have high-quality data sets for model training, but also could create a certain level of bias for the study population.  This observation that more advanced machine learning models did not perform better compared to regularized logistic regression could be due to the relatively small sample size and presence of a few strongly predictive features (eg, albumin, SpO2, and temperature), under which the power of machine learning models in handling large and complex data could not be fully leveraged.  The study cohort mainly comes from a prevaccine stage (March 2020 and May 2021), so they did not include vaccine status in their model. Due to this characteristic of the study population, this model will be more closely applicable to a nonvaccinated population and not well generalized to other settings. | | | |

**Step 4: Overall assessment**

| | **Reaching an overall judgement about risk of bias of the prediction model evaluation** | | | --- | --- | | **Low risk of bias** | If all domains were rated low risk of bias.  If a prediction model was developed without any external validation, and it was rated as low risk of bias for all domains, consider downgrading to **high risk of bias**. Such a model can only be considered as low risk of bias, if the development was based on a very large data set and included some form of internal validation. | | **High risk of bias** | If at least one domain is judged to be at **high risk of bias**. | | **Unclear risk of bias** | If an unclear risk of bias was noted in at least one domain and it was low risk for all other domains. |  | **Reaching an overall judgement about applicability of the prediction model evaluation** | | | --- | --- | | **Low concerns regarding applicability** | If low concerns regarding applicability for all domains, the prediction model evaluation is judged to have **low concerns regarding applicability**. | | **High concerns regarding applicability** | If high concerns regarding applicability for at least one domain, the prediction model evaluation is judged to have **high concerns regarding applicability**. | | **Unclear concerns regarding applicability** | If unclear concerns (but no “high concern”) regarding applicability for at least one domain, the prediction model evaluation is judged to have **unclear concerns regarding applicability** overall. | |
| --- | --- | --- | --- | --- | --- | --- | --- | --- | --- | --- | --- | --- | --- | --- | --- | --- |

| **Overall judgement about risk of bias and applicability of the prediction model evaluation** | | |
| --- | --- | --- |
| **Overall judgement of risk of bias** | **RISK:**  *(low/ high/ unclear)* | **high** |
| *Summary of sources of potential bias:*  There was not enough data to evaluate appropriately the model used for the prediction.  This study did not describe any causal relationship between the predictor and outcome variables. | | |
| **Overall judgement of applicability** | **CONCERN:**  *(low/ high/ unclear)* | **high** |
| *Summary of applicability concerns:*  The time interval of outcomes calculated whether the patient was readmitted on the last visit within 30 days after being discharged from the hospital on the penultimate visit. Choosing the 30 days may not be appropriate and can cause different results in applying the model in other settings.  The analyses were based on a single-center dataset, therefore the results of this study may not be generalizable. | | |

**Appendix 3**. Willette 2022 - Bias analysis using PROBAST.

**Step 1: Specify your systematic review question**

| **Criteria** | **Specify your systematic review question** |
| --- | --- |
| *Intended use of model:* | To predict COVID‑19 infection and severity risk |
| ***Participants*** *including selection criteria and setting:* | older adults  and positive tested COVID-19 older adults |
| ***Predictors*** *(used in prediction modelling), including types of predictors (e.g. history, clinical examination, biochemical markers, imaging tests), time of measurement, specific measurement issues (e.g., any requirements/ prohibitions for specialized equipment):* | demographics, health behaviors and long-term disability or illness status, anthropometric and bioimpedance measures of fat, muscle, or water content, pulse and blood pressure, a serum panel of thirty biochemistry markers commonly collected in a clinic or hospital setting, and a complete blood count with a manual differential |
| *Outcome to be predicted:* | COVID‑19 infection and hospitaliation |

**Step 2: Classify the type of prediction model evaluation**

| **Classify the evaluation based on its aim** | | | |
| --- | --- | --- | --- |
| **Type of prediction study** | **PROBAST boxes to complete** | **Tick as appropriate** | **Definition for type of prediction model study** |
| Development only | Development | ✔ | Prediction model development without external validation. These studies may include internal validation methods, such as bootstrapping and cross-validation techniques. |
| Development and validation | Development and validation | ✖ | Prediction model development combined with external validation in other participants in the same article. |
| Validation only | Validation | ✖ | External validation of existing (previously developed) model in other participants. |

|  | |
| --- | --- |
| **Publication reference** | Willette AA, Willette SA, Wang Q, Pappas C, Klinedinst BS, Le S, Larsen B, Pollpeter A, Li T, Mochel JP, Allenspach K, Brenner N, Waterboer T. Using machine learning to predict COVID-19 infection and severity risk among 4510 aged adults: a UK Biobank cohort study. Sci Rep. 2022 May 11;12(1):7736. doi: 10.1038/s41598-022-07307-z. PMID: 35545624; PMCID: PMC9092926. | |
| **Models of interest** | Linear discriminant analysis |  |
| **Outcome of interest** | Hospitalization | |

**Step 3: Assess risk of bias and applicability**

| **DOMAIN 1:  Participants** | | | |
| --- | --- | --- | --- |
| **A. Risk of Bias** | | | |
| *Describe the sources of data and criteria for participant selection:*  DEV:  This retrospective study involved the UK Biobank cohort. Baseline data was collected in 2006–2010 at 22 centers across the United Kingdom. The study was based on COVID PCR test data available from March 16th to May 19th 2020. Specifcally, they used the May 26th, 2020 tranche of COVID-19 polymerase chain reaction (PCR) data from Public Health England. | | | |
|  | | Dev | Val |
| 1. Were appropriate data sources used, e.g. cohort, RCT or nested case-control study data? | | Y |  |
| 1. Were all inclusions and exclusions of participants appropriate? | | PY |  |
| **Risk of bias introduced by selection of participants** | **RISK:**  *(low/ high/ unclear)* | **low** |  |
| *Rationale of bias rating: No Concerns* | | | |
|  | | | |
| **B. Applicability** | | | |
| *Describe included participants, setting and dates:*  It has been mentioned in Domain 1 – Section A. | | | |
| **Concern that the included participants and setting do not match the review question** | **CONCERN:**  *(low/ high/ unclear)* | **low** |  |
| *Rationale of applicability rating:* | | | |
| In our review, our source population is the general population. Therefore, this data is part of the general population. | | | |

| **DOMAIN 2:  Predictors** | | | |
| --- | --- | --- | --- |
| **A. Risk of Bias** | | | |
| *List and describe predictors included in the final model, e.g. definition and timing of assessment:*  age, immune markers, lipids, and serology titers to common pathogens like human cytomegalovirus | | | |
|  | | Dev | Val |
| 1. Were predictors defined and assessed in a similar way for all participants? | | PY |  |
| 1. Were predictor assessments made without knowledge of outcome data? | | PY |  |
| 1. Are all predictors available at the time the model is intended to be used? | | Y |  |
| **Risk of bias introduced by predictors or their assessment** | **RISK:**  *(low/ high/ unclear)* | **low** |  |
| *Rationale of bias rating: No Concerns* | | | |
| **B. Applicability** | | | |
| Concern that the definition, assessment or timing of predictors in the model do not match the review question | **CONCERN:**  *(low/ high/ unclear)* | **Low** |  |
| *Rationale of applicability rating: No Concerns* | | | |

| **DOMAIN 3: Outcome** | | | |
| --- | --- | --- | --- |
| **A. Risk of Bias** | | | |
| *Describe the outcome, how it was defined and determined, and the time interval between predictor assessment and outcome determination:* | | | |
|  | | Dev | Val |
| 1. Was the outcome determined appropriately? | | PY |  |
| 1. Was a pre-specified or standard outcome definition used? | | PY |  |
| 1. Were predictors excluded from the outcome definition? | | Y |  |
| 1. Was the outcome defined and determined in a similar way for all participants? | | PY |  |
| 1. Was the outcome determined without knowledge of predictor information? | | PY |  |
| 1. Was the time interval between predictor assessment and outcome determination appropriate? | | NI |  |
| **Risk of bias introduced by the outcome or its determination** | **RISK:**  *(low/ high/ unclear)* | **high** |  |
| *Rationale of bias rating:*  It is not clear how the time interval between predictor assessment and outcome was determined. | | | |
| **B. Applicability** | | | |
| *At what time point was the outcome determined:*  *If a composite outcome was used, describe the relative frequency/distribution of each contributing outcome:* | | | |
| **Concern that the outcome, its definition, timing or determination do not match the review question** | **CONCERN:**  *(low/ high/ unclear)* | **high** |  |
| *Rationale of applicability rating:*  It is not clear how the time interval between predictor assessment and outcome was determined. | | | |
| **DOMAIN 4: Analysis** | | | |
| **Risk of Bias** | | | |
| *Describe numbers of participants, number of candidate predictors, outcome events and events per candidate predictor:*  DEV: 2210 positive cases. 996 mild and 1214 presumptively severe disease outcomes | | | |
| *Describe how the model was developed (for example in regards to modelling technique (e.g. survival or logistic modelling), predictor selection, and risk group definition):*  LDA | | | |
| *Describe whether and how the model was validated, either internally (e.g. bootstrapping, cross validation, random split sample) or externally (e.g. temporal validation, geographical validation, different setting, different type of participants):*  Cross validation was used. | | | |
| *Describe the performance measures of the model, e.g. (re)calibration, discrimination, (re)classification, net benefit, and whether they were adjusted for optimism:*  The ROC analysis was performed. | | | |
| *Describe any participants who were excluded from the analysis:*  Not mentioned clearly | | | |
| *Describe missing data on predictors and outcomes as well as methods used for missing data:*  No information was found how to handle missing values | | | |
|  | | Dev | Val |
| 1. Were there a reasonable number of participants with the outcome? | | Y |  |
| 1. Were continuous and categorical predictors handled appropriately? | | NI |  |
| 1. Were all enrolled participants included in the analysis? | | PY |  |
| 1. Were participants with missing data handled appropriately? | | NI |  |
| 1. Was selection of predictors based on univariable analysis avoided? | | NI |  |
| 1. Were complexities in the data (e.g. censoring, competing risks, sampling of controls) accounted for appropriately? | | PY |  |
| 1. Were relevant model performance measures evaluated appropriately? | | Y |  |
| 1. Were model overfitting and optimism in model performance accounted for? | | PY |  |
| 1. Do predictors and their assigned weights in the final model correspond to the results from multivariable analysis? | | PY |  |
| **Risk of bias introduced by the analysis** | **RISK:**  *(low/ high/ unclear)* | **high** |  |
| *Rationale of bias rating:*  Other ML models were not implemented to compare with the current model used in this study. They only looked at the so-called main effects of all predictors instead of complex interactions. | | | |

**Step 4: Overall assessment**

| | **Reaching an overall judgement about risk of bias of the prediction model evaluation** | | | --- | --- | | **Low risk of bias** | If all domains were rated low risk of bias.  If a prediction model was developed without any external validation, and it was rated as low risk of bias for all domains, consider downgrading to **high risk of bias**. Such a model can only be considered as low risk of bias, if the development was based on a very large data set and included some form of internal validation. | | **High risk of bias** | If at least one domain is judged to be at **high risk of bias**. | | **Unclear risk of bias** | If an unclear risk of bias was noted in at least one domain and it was low risk for all other domains. |  | **Reaching an overall judgement about applicability of the prediction model evaluation** | | | --- | --- | | **Low concerns regarding applicability** | If low concerns regarding applicability for all domains, the prediction model evaluation is judged to have **low concerns regarding applicability**. | | **High concerns regarding applicability** | If high concerns regarding applicability for at least one domain, the prediction model evaluation is judged to have **high concerns regarding applicability**. | | **Unclear concerns regarding applicability** | If unclear concerns (but no “high concern”) regarding applicability for at least one domain, the prediction model evaluation is judged to have **unclear concerns regarding applicability** overall. | |
| --- | --- | --- | --- | --- | --- | --- | --- | --- | --- | --- | --- | --- | --- | --- | --- | --- |

| **Overall judgement about risk of bias and applicability of the prediction model evaluation** | | |
| --- | --- | --- |
| **Overall judgement of risk of bias** | **RISK:**  *(low/ high/ unclear)* | **high** |
| *Summary of sources of potential bias:*  It is not clear how the time interval between predictor assessment and outcome was determined.  No information was found how to handle missing values.  Other ML models were not implemented to compare with the current model used in this study. They only looked at the so-called main effects of all predictors instead of complex interactions | | |
| **Overall judgement of applicability** | **CONCERN:**  *(low/ high/ unclear)* | **high** |
| *Summary of applicability concerns:*  The number of UK Biobank participants with COVID-19 and serology data is low, particularly for positive test cases. This could consequently lead to model overfitting or misestimation. Therefore the developed model could not be generalized to other setting. | | |

**Appendix 4**. Jakob 2022 - Bias analysis using PROBAST.

**Step 1: Specify your systematic review question**

| **Criteria** | **Specify your systematic review question** |
| --- | --- |
| *Intended use of model:* | To identify patients at risk of progressing to advanced COVID-19 |
| ***Participants*** *including selection criteria and setting:* | Patients positive to COVID-19. |
| ***Predictors*** *(used in prediction modelling), including types of predictors (e.g. history, clinical examination, biochemical markers, imaging tests), time of measurement, specific measurement issues (e.g., any requirements/ prohibitions for specialized equipment):* | Demographics, Clinical |
| *Outcome to be predicted:* | Hospitalization following COVID-19 diagnosis. |

**Step 2: Classify the type of prediction model evaluation**

| **Classify the evaluation based on its aim** | | | |
| --- | --- | --- | --- |
| **Type of prediction study** | **PROBAST boxes to complete** | **Tick as appropriate** | **Definition for type of prediction model study** |
| Development only | Development | ✖ | Prediction model development without external validation. These studies may include internal validation methods, such as bootstrapping and cross-validation techniques. |
| Development and validation | Development and validation | ✔ | Prediction model development combined with external validation in other participants in the same article. |
| Validation only | Validation | ✖ | External validation of existing (previously developed) model in other participants. |

|  | |
| --- | --- |
| **Publication reference** | Jakob CEM, Mahajan UM, Oswald M, Stecher M, Schons M, Mayerle J, Rieg S, Pletz M, Merle U, Wille K, Borgmann S, Spinner CD, Dolff S, Scherer C, Pilgram L, Rüthrich M, Hanses F, Hower M, Strauß R, Massberg S, Er AG, Jung N, Vehreschild JJ, Stubbe H, Tometten L, König R; LEOSS Study group. Prediction of COVID-19 deterioration in high-risk patients at diagnosis: an early warning score for advanced COVID-19 developed by machine learning. Infection. 2022 Apr;50(2):359-370. doi: 10.1007/s15010-021-01656-z. Epub 2021 Jul 19. PMID: 34279815; PMCID: PMC8287547. | |
| **Models of interest** | Random forests, gradient boosting machines (gbm), extreme gradient boosting (XGBoost) and StackedEnsemble | |
| **Outcome of interest** | Hospitalization | |

**Step 3: Assess risk of bias and applicability**

| **DOMAIN 1:  Participants** | | | |
| --- | --- | --- | --- |
| **A. Risk of Bias** | | | |
| *Describe the sources of data and criteria for participant selection:*  DEV:  Clinical data from SARS-CoV-2 positive patients from the multicenter Lean European Open Survey on SARS-CoV-2 Infected Patients (LEOSS) were used from 2020-03-16 to 2020-07-14. LEOSS is the multicenter international COVID-19 registry comprising over 7000 patients collected in more than 100 study sites (http:// www.leoss.net).  Inclusion criteria for LEOSS were a laboratory confrmed SARS-CoV-2 infection from any respiratory material and clinical information available on follow-up until the end of the treatment (recovery or death). In this study, patients were included who were asymptomatic or exhibited mild symptoms (symptoms of the upper respiratory tract, fever, nausea, emesis or diarrhea) at baseline. They included 1223 out of 2819 patients enrolled in LEOSS for model discovery.  The baseline data comprised patient characteristics, symptoms, co-morbidities, known microbiological colonization, preexisting medication, and laboratory and vital parameters.  VAL:  the validation data set extracted from the same dataset but in different time period: patients enrolled from 2020-07-15 to 2021-02-16. They included 2264 out of 3541 patients enrolled in LEOSS for validation | | | |
|  | | Dev | Val |
| 1. Were appropriate data sources used, e.g. cohort, RCT or nested case-control study data? | | Y | PY |
| 1. Were all inclusions and exclusions of participants appropriate? | | PN | PN |
| **Risk of bias introduced by selection of participants** | **RISK:**  *(low/ high/ unclear)* | **unclear** | **unclear** |
| *Rationale of bias rating:* | | | |
| There is a concern related to the exclusion criteria. The authors excluded patients with advanced COVID-19 stages at baseline. Furthermore, for the development of the algorithm and SACOV-19, they excluded patients with no documented information on laboratory or vital data (n = 279). | | | |
| **B. Applicability** | | | |
| *Describe included participants, setting and dates:*  It has been mentioned in Domain 1 – Section A. | | | |
| **Concern that the included participants and setting do not match the review question** | **CONCERN:**  *(low/ high/ unclear)* | **low** | **low** |
| *Rationale of applicability rating:* | | | |
| In our review, our source population is the general population. Therefore, LEOSS dataset is part of the general population. | | | |

| **DOMAIN 2:  Predictors** | | | |
| --- | --- | --- | --- |
| **A. Risk of Bias** | | | |
| *List and describe predictors included in the final model, e.g. definition and timing of assessment:*  The LEOSS dataset contains 473 baseline patient parameters measured at the first patient contact. After training the predictor model on a training dataset comprising 1233 patients, 20 of the 473 parameters were selected for the predictor model. From the predictor model, they delineated a composite predictive score (SACOV-19, Score for the prediction of an Advanced stage of COVID-19) with eleven variables. In the validation cohort (n=2264 patients), we observed good prediction performance with an area under the curve (AUC) of 0.73±0.01. Besides temperature, age, body mass index and smoking habit, variables indicating pulmonary involvement (respiration rate, oxygen saturation, dyspnea), infammation (CRP, LDH, lymphocyte counts), and acute kidney injury at diagnosis were identified. | | | |
|  | | Dev | Val |
| 1. Were predictors defined and assessed in a similar way for all participants? | | Y | Y |
| 1. Were predictor assessments made without knowledge of outcome data? | | Y | Y |
| 1. Are all predictors available at the time the model is intended to be used? | | Y | Y |
| **Risk of bias introduced by predictors or their assessment** | **RISK:**  *(low/ high/ unclear)* | **low** | **low** |
| *Rationale of bias rating:* | | | |
| **B. Applicability** | | | |
| Concern that the definition, assessment or timing of predictors in the model do not match the review question | **CONCERN:**  *(low/ high/ unclear)* | **low** | **low** |
| *Rationale of applicability rating:*  Predictors were assessed prior to the occurrence of the outcome. Therefore, all predictors included in this article can be considered valid. | | | |

| **DOMAIN 3: Outcome** | | | |
| --- | --- | --- | --- |
| **A. Risk of Bias** | | | |
| *Describe the outcome, how it was defined and determined, and the time interval between predictor assessment and outcome determination:*  Progression to a complicated or severe stage of COVID-19 during medical consultation/observational period was set as the endpoint (denoted as advanced COVID-19 stage). It was defined by the occurrence of at least one of the following symptoms during the observational period (complicated or critical COVID-19 stage according to LEOSS criteria): need for new oxygen supplementation due to clinical deterioration, oxygen saturation (SO2) at room air < 90%, partial pressure of oxygen (PaO2) at room air < 70 mmHg, clinically meaningful increase of oxygen supplementation compared to prior oxygen home therapy, increase of aspartate aminotransferase (AST) or alanine aminotransferase (ALT) > 5 × ULN (upper limit of normal), new cardiac arrhythmia, new pericardial effusion > 1 cm or new heart failure with pulmonary edema, congestive hepatopathy or peripheral edema, catecholamine therapy, life-threatening cardiac arrhythmia, liver failure with an INR > 3.5 (Quick < 50%), a qSOFA score of ≥ 2 or acute renal failure with need of dialysis. | | | |
|  | | Dev | Val |
| 1. Was the outcome determined appropriately? | | PY | PY |
| 1. Was a pre-specified or standard outcome definition used? | | PN | PN |
| 1. Were predictors excluded from the outcome definition? | | Y | Y |
| 1. Was the outcome defined and determined in a similar way for all participants? | | Y | Y |
| 1. Was the outcome determined without knowledge of predictor information? | | PY | PY |
| 1. Was the time interval between predictor assessment and outcome determination appropriate? | | PY | PY |
| **Risk of bias introduced by the outcome or its determination** | **RISK:**  *(low/ high/ unclear)* | **unclear** | **unclear** |
| *Rationale of bias rating:*  We cannot exclude other aetiology for Progression to a complicated or severe stage of COVID-19 after the patient left the hospital. | | | |
| **B. Applicability** | | | |
| *At what time point was the outcome determined:*    *If a composite outcome was used, describe the relative frequency/distribution of each contributing outcome:* | | | |
| **Concern that the outcome, its definition, timing or determination do not match the review question** | **CONCERN:**  *(low/ high/ unclear)* | **unclear** | **unclear** |
| *Rationale of applicability rating:*  The study suffers from low patient numbers to test the models for outpatients. Only 28, after removal of patients with at least one NA in the score variables. | | | |

| **DOMAIN 4: Analysis** | | | |
| --- | --- | --- | --- |
| **Risk of Bias** | | | |
| *Describe numbers of participants, number of candidate predictors, outcome events and events per candidate predictor:*  DEV: 1223 participants, 473 candidate predictors and then 20 variables were selected  VAL: 2264 participants with 801 Patients which advanced to the advanced COVID-19 stage. and 20 candidate predictors | | | |
| *Describe how the model was developed (for example in regards to modelling technique (e.g. survival or logistic modelling), predictor selection, and risk group definition):*  Random forests, gradient boosting machines (gbm), extreme gradient boosting (XGBoost) and StackedEnsemble. | | | |
| *Describe whether and how the model was validated, either internally (e.g. bootstrapping, cross validation, random split sample) or externally (e.g. temporal validation, Geographical validation, different setting, different type of participants):*  cross validation was performed. | | | |
| *Describe the performance measures of the model, e.g. (re)calibration, discrimination, (re)classification, net benefit, and whether they were adjusted for optimism:*  A ROC analysis was performed using a separate 2 × 2 “confusion matrix” for every possible threshold applied to the total score. The best threshold was defined by the highest value of the Youden Index. Point estimates and confidence intervals were computed using the bootstrap for sensitivity, specificity, Positive Predictive Value (PPV), Negative Predictive Value (NPV) and the De Long method for the Area Under the Curve (AUC)  Reliability analysis of the predictive formula was summarized using ROC curves showing optimal thresholds and sensitivity, specificity, AUC with point estimates and 95% confidence intervals. | | | |
| *Describe any participants who were excluded from the analysis:*  They excluded patients with advanced COVID-19 stages at baseline. Furthermore, for the development of the algorithm and SACOV-19, they excluded patients with no documented information on laboratory or vital data (n = 279). | | | |
| *Describe missing data on predictors and outcomes as well as methods used for missing data:*  Not available. | | | |
|  | | Dev | Val |
| 1. Were there a reasonable number of participants with the outcome? | | NI | NI |
| 1. Were continuous and categorical predictors handled appropriately? | | PY | PY |
| 1. Were all enrolled participants included in the analysis? | | PY | PY |
| 1. Were participants with missing data handled appropriately? | | NI | NI |
| 1. Was selection of predictors based on univariable analysis avoided? | | Y | Y |
| 1. Were complexities in the data (e.g. censoring, competing risks, sampling of controls) accounted for appropriately? | | Y | Y |
| 1. Were relevant model performance measures evaluated appropriately? | | Y | Y |
| 1. Were model overfitting and optimism in model performance accounted for? | | Y | Y |
| 1. Do predictors and their assigned weights in the final model correspond to the results from multivariable analysis? | | NI | NI |
| **Risk of bias introduced by the analysis** | **RISK:**  *(low/ high/ unclear)* | **high** | **high** |
| *Rationale of bias rating:*  Most of the patients received care in an inpatient setting. When testing their score on outpatients they observed a similar performance result, however, they had only n=28 outpatients for this analysis and could hence not get a significant result. Furthermore, the majority of patients exhibited a mild disease and did not advance to the complicated phase. Therefore, patients with co-morbidities could have been overrepresented in their cohort, as these patients were mainly admitted without severe symptoms. To show the general applicability of their score, a further, clinical trial is necessary. | | | |

**Step 4: Overall assessment**

| | **Reaching an overall judgement about risk of bias of the prediction model evaluation** | | | --- | --- | | **Low risk of bias** | If all domains were rated low risk of bias.  If a prediction model was developed without any external validation, and it was rated as low risk of bias for all domains, consider downgrading to **high risk of bias**. Such a model can only be considered as low risk of bias, if the development was based on a very large data set and included some form of internal validation. | | **High risk of bias** | If at least one domain is judged to be at **high risk of bias**. | | **Unclear risk of bias** | If an unclear risk of bias was noted in at least one domain and it was low risk for all other domains. |  | **Reaching an overall judgement about applicability of the prediction model evaluation** | | | --- | --- | | **Low concerns regarding applicability** | If low concerns regarding applicability for all domains, the prediction model evaluation is judged to have **low concerns regarding applicability**. | | **High concerns regarding applicability** | If high concerns regarding applicability for at least one domain, the prediction model evaluation is judged to have **high concerns regarding applicability**. | | **Unclear concerns regarding applicability** | If unclear concerns (but no “high concern”) regarding applicability for at least one domain, the prediction model evaluation is judged to have **unclear concerns regarding applicability** overall. | |
| --- | --- | --- | --- | --- | --- | --- | --- | --- | --- | --- | --- | --- | --- | --- | --- | --- |

| **Overall judgement about risk of bias and applicability of the prediction model evaluation** | | |
| --- | --- | --- |
| **Overall judgement of risk of bias** | **RISK:**  *(low/ high/ unclear)* | **high** |
| *Summary of sources of potential bias:*  We cannot exclude other aetiology for Progression to a complicated or severe stage of COVID-19 after the patient left the hospital.  Most of the patients received care in an inpatient setting. When testing their score on outpatients they observed a similar performance result, however, they had only n=28 outpatients for this analysis and could hence not get a significant result. Furthermore, the majority of patients exhibited a mild disease and did not advance to the complicated phase. Therefore, patients with co-morbidities could have been overrepresented in their cohort, as these patients were mainly admitted without severe symptoms. To show the general applicability of their score, a further, clinical trial is necessary. | | |
| **Overall judgement of applicability** | **CONCERN:**  *(low/ high/ unclear)* | **high** |
| *Summary of applicability concerns:*  The study suffers from low patient numbers to test the models. | | |

**Appendix 5**. Hernández-Pereira 2022 - Bias analysis using PROBAST.

**Step 1: Specify your systematic review question**

| **Criteria** | **Specify your systematic review question** |
| --- | --- |
| *Intended use of model:* | To predict different levels of hospitalization (regular hospital admission or intensive care unit admission) for CoVid-19 patients |
| ***Participants*** *including selection criteria and setting:* | positive tested COVID-19 patients |
| ***Predictors*** *(used in prediction modelling), including types of predictors (e.g. history, clinical examination, biochemical markers, imaging tests), time of measurement, specific measurement issues (e.g., any requirements/ prohibitions for specialized equipment):* | demographics, Clinical |
| *Outcome to be predicted:* | Hospitalization or intensive care admission |

**Step 2: Classify the type of prediction model evaluation**

| **Classify the evaluation based on its aim** | | | |
| --- | --- | --- | --- |
| **Type of prediction study** | **PROBAST boxes to complete** | **Tick as appropriate** | **Definition for type of prediction model study** |
| Development only | Development | ✔ | Prediction model development without external validation. These studies may include internal validation methods, such as bootstrapping and cross-validation techniques. |
| Development and validation | Development and validation | ✖ | Prediction model development combined with external validation in other participants in the same article. |
| Validation only | Validation | ✖ | External validation of existing (previously developed) model in other participants. |

|  | |
| --- | --- |
| **Publication reference** | Hernández-Pereira E, Fontenla-Romero O, Bolón-Canedo V, Cancela-Barizo B, Guijarro-Berdiñas B, Alonso-Betanzos A. Machine learning techniques to predict different levels of hospital care of CoVid-19. Appl Intell (Dordr). 2022;52(6):6413-6431. doi: 10.1007/s10489-021-02743-2. Epub 2021 Sep 10. PMID: 34764619; PMCID: PMC8429889. | |
| **Models of interest** | LR, MLR, SVM Linear, SVM RBF,  knn, AdaBoost, Bagging, RF, DeepNetwork |  |
| **Outcome of interest** | Hospitalization or intensive care admission | |

**Step 3: Assess risk of bias and applicability**

| **DOMAIN 1:  Participants** | | | |
| --- | --- | --- | --- |
| **A. Risk of Bias** | | | |
| *Describe the sources of data and criteria for participant selection:*  DEV:  For this research, a data set of 10,454 patients from 14 hospitals in Galicia (Spain) was used. Each patient is characterized by 833 variables including demographic and clinical datasets from March to May, 2020. | | | |
|  | | Dev | Val |
| 1. Were appropriate data sources used, e.g. cohort, RCT or nested case-control study data? | | Y |  |
| 1. Were all inclusions and exclusions of participants appropriate? | | NI |  |
| **Risk of bias introduced by selection of participants** | **RISK:**  *(low/ high/ unclear)* | **unclear** |  |
| *Rationale of bias rating: The dataset were collected only for 3 months period.* | | | |
|  | | | |
| **B. Applicability** | | | |
| *Describe included participants, setting and dates:*  It has been mentioned in Domain 1 – Section A. | | | |
| **Concern that the included participants and setting do not match the review question** | **CONCERN:**  *(low/ high/ unclear)* | **unclear** |  |
| *Rationale of applicability rating:* | | | |
| In our review, our source population is the general population. Therefore, this data is part of the general population. However the data were collected only within 3 months from March to May 2020. | | | |

| **DOMAIN 2:  Predictors** | | | |
| --- | --- | --- | --- |
| **A. Risk of Bias** | | | |
| *List and describe predictors included in the final model, e.g. definition and timing of assessment:*  For hospitalization: Age, Gender, Non-insulin dependent diabetes mellitus, Benign prostatic hypertrophy, Other diagnostic procedures, Hydrocele, Viral pneumonia, Therapeutic advice/therapeutic listening, Urgent / frequent urination, Abnormal white blood cells, Other disorders of lipid metabolism, Heart failure | | | |
|  | | Dev | Val |
| 1. Were predictors defined and assessed in a similar way for all participants? | | PY |  |
| 1. Were predictor assessments made without knowledge of outcome data? | | PY |  |
| 1. Are all predictors available at the time the model is intended to be used? | | Y |  |
| **Risk of bias introduced by predictors or their assessment** | **RISK:**  *(low/ high/ unclear)* | **high** |  |
| *Rationale of bias rating:* Variables included Age, Gender and the 831 binary variables of the patient’s medical history. However age is a continuous variable which is not scaled compared to other binary variables and leads to bias for the machine learning results. | | | |
| **B. Applicability** | | | |
| Concern that the definition, assessment or timing of predictors in the model do not match the review question | **CONCERN:**  *(low/ high/ unclear)* | **high** |  |
| *Rationale of applicability rating:*  *There is a short period (3 months) for assessing and timing of predictors in the models.* | | | |

| **DOMAIN 3: Outcome** | | | |
| --- | --- | --- | --- |
| **A. Risk of Bias** | | | |
| *Describe the outcome, how it was defined and determined, and the time interval between predictor assessment and outcome determination:* | | | |
|  | | Dev | Val |
| 1. Was the outcome determined appropriately? | | PY |  |
| 1. Was a pre-specified or standard outcome definition used? | | PY |  |
| 1. Were predictors excluded from the outcome definition? | | Y |  |
| 1. Was the outcome defined and determined in a similar way for all participants? | | PY |  |
| 1. Was the outcome determined without knowledge of predictor information? | | PY |  |
| 1. Was the time interval between predictor assessment and outcome determination appropriate? | | NI |  |
| **Risk of bias introduced by the outcome or its determination** | **RISK:**  *(low/ high/ unclear)* | **high** |  |
| *Rationale of bias rating:*  It is not clear how the time interval between predictor assessment and outcome was determined. | | | |
| **B. Applicability** | | | |
| *At what time point was the outcome determined:*  *If a composite outcome was used, describe the relative frequency/distribution of each contributing outcome:* | | | |
| **Concern that the outcome, its definition, timing or determination do not match the review question** | **CONCERN:**  *(low/ high/ unclear)* | **high** |  |
| *Rationale of applicability rating:*  It is not clear how the time interval between predictor assessment and outcome was determined. | | | |
| **DOMAIN 4: Analysis** | | | |
| **Risk of Bias** | | | |
| *Describe numbers of participants, number of candidate predictors, outcome events and events per candidate predictor:*  DEV:  3,024 patients were hospitalized( 28.9%) and 7,430 controls. 300 patients were admitted to the intensive care unit (2.9%) and 10,154 controls | | | |
| *Describe how the model was developed (for example in regards to modelling technique (e.g. survival or logistic modelling), predictor selection, and risk group definition):*  LR, MLR, SVM Linear, SVM RBF,  knn, AdaBoost, Bagging, RF, DeepNetwork | | | |
| *Describe whether and how the model was validated, either internally (e.g. bootstrapping, cross validation, random split sample) or externally (e.g. temporal validation, geographical validation, different setting, different type of participants):*  Cross validation was used. | | | |
| *Describe the performance measures of the model, e.g. (re)calibration, discrimination, (re)classification, net benefit, and whether they were adjusted for optimism:*  The ROC analysis was performed. | | | |
| *Describe any participants who were excluded from the analysis:*  Not mentioned clearly | | | |
| *Describe missing data on predictors and outcomes as well as methods used for missing data:*  No information was found how to handle missing values | | | |
|  | | Dev | Val |
| 1. Were there a reasonable number of participants with the outcome? | | PN |  |
| 1. Were continuous and categorical predictors handled appropriately? | | N |  |
| 1. Were all enrolled participants included in the analysis? | | PY |  |
| 1. Were participants with missing data handled appropriately? | | NI |  |
| 1. Was selection of predictors based on univariable analysis avoided? | | NI |  |
| 1. Were complexities in the data (e.g. censoring, competing risks, sampling of controls) accounted for appropriately? | | PY |  |
| 1. Were relevant model performance measures evaluated appropriately? | | Y |  |
| 1. Were model overfitting and optimism in model performance accounted for? | | PY |  |
| 1. Do predictors and their assigned weights in the final model correspond to the results from multivariable analysis? | | PY |  |
| **Risk of bias introduced by the analysis** | **RISK:**  *(low/ high/ unclear)* | **high** |  |
| *Rationale of bias rating:*  The dataset was highly unbalanced and oversampling might cause bias in the results. Age is a continuous variable which is not scaled compared to other binary variables and leads to bias for the machine learning results. | | | |

**Step 4: Overall assessment**

| | **Reaching an overall judgement about risk of bias of the prediction model evaluation** | | | --- | --- | | **Low risk of bias** | If all domains were rated low risk of bias.  If a prediction model was developed without any external validation, and it was rated as low risk of bias for all domains, consider downgrading to **high risk of bias**. Such a model can only be considered as low risk of bias, if the development was based on a very large data set and included some form of internal validation. | | **High risk of bias** | If at least one domain is judged to be at **high risk of bias**. | | **Unclear risk of bias** | If an unclear risk of bias was noted in at least one domain and it was low risk for all other domains. |  | **Reaching an overall judgement about applicability of the prediction model evaluation** | | | --- | --- | | **Low concerns regarding applicability** | If low concerns regarding applicability for all domains, the prediction model evaluation is judged to have **low concerns regarding applicability**. | | **High concerns regarding applicability** | If high concerns regarding applicability for at least one domain, the prediction model evaluation is judged to have **high concerns regarding applicability**. | | **Unclear concerns regarding applicability** | If unclear concerns (but no “high concern”) regarding applicability for at least one domain, the prediction model evaluation is judged to have **unclear concerns regarding applicability** overall. | |
| --- | --- | --- | --- | --- | --- | --- | --- | --- | --- | --- | --- | --- | --- | --- | --- | --- |

| **Overall judgement about risk of bias and applicability of the prediction model evaluation** | | |
| --- | --- | --- |
| **Overall judgement of risk of bias** | **RISK:**  *(low/ high/ unclear)* | **high** |
| *Summary of sources of potential bias:*  It is not clear how the time interval between predictor assessment and outcome was determined.  No information was found how to handle missing values. | | |
| **Overall judgement of applicability** | **CONCERN:**  *(low/ high/ unclear)* | **high** |
| *Summary of applicability concerns:*  The dataset was highly unbalanced and oversampling might cause bias in the results. Age is a continuous variable which is not scaled compared to other binary variables and leads to bias for the machine learning results.  *There is a short period (3 months) for assessing and timing of predictors in the models.* | | |

**Appendix 6**. Gutierrez 2022 - Bias analysis using PROBAST.

**Step 1: Specify your systematic review question**

| **Criteria** | **Specify your systematic review question** |
| --- | --- |
| *Intended use of model:* | to develop and validate a multivariable model to predict risk of hospitalization for patients infected with SARS-CoV-2. |
| ***Participants*** *including selection criteria and setting:* | positive tested COVID-19 patients |
| ***Predictors*** *(used in prediction modelling), including types of predictors (e.g. history, clinical examination, biochemical markers, imaging tests), time of measurement, specific measurement issues (e.g., any requirements/ prohibitions for specialized equipment):* | demographics, Clinical |
| *Outcome to be predicted:* | Hospitalization |

**Step 2: Classify the type of prediction model evaluation**

| **Classify the evaluation based on its aim** | | | |
| --- | --- | --- | --- |
| **Type of prediction study** | **PROBAST boxes to complete** | **Tick as appropriate** | **Definition for type of prediction model study** |
| Development only | Development | ✔ | Prediction model development without external validation. These studies may include internal validation methods, such as bootstrapping and cross-validation techniques. |
| Development and validation | Development and validation | ✖ | Prediction model development combined with external validation in other participants in the same article. |
| Validation only | Validation | ✖ | External validation of existing (previously developed) model in other participants. |

|  | |
| --- | --- |
| **Publication reference** | Gutierrez JM, Volkovs M, Poutanen T, Watson T, Rosella LC. Risk stratification for COVID-19 hospitalization: a multivariable model based on gradient-boosting decision trees. CMAJ Open. 2021 Dec 21;9(4):E1223-E1231. doi: 10.9778/cmajo.20210036. PMID: 34933880; PMCID: PMC8695533. | |
| **Models of interest** | Gradient-boosting decision trees, SHAP |  |
| **Outcome of interest** | Hospitalization | |

**Step 3: Assess risk of bias and applicability**

| **DOMAIN 1:  Participants** | | | |
| --- | --- | --- | --- |
| **A. Risk of Bias** | | | |
| *Describe the sources of data and criteria for participant selection:*  DEV: The data included in this study are based on all laboratory-confirmed cases of SARS-CoV-2 infection compiled and held at ICES, a not-for-profit research institute in Ontario. This cohort included adult patients (age ≥ 18 yr) identified through the Ontario Laboratories Information System (OLIS) between Feb. 2 and Oct. 5, 2020, and were followed up through Nov. 5, 2020. They linked to other health data containing demographic, health care use and area-level information using unique encoded identifiers.  Patients living in long-term care facilities were excluded, as they were all assumed to be at high risk of hospitalization for COVID-19. Risk of hospitalization within 30 days of diagnosis of SARS-CoV-2 infection was estimated. | | | |
|  | | Dev | Val |
| 1. Were appropriate data sources used, e.g. cohort, RCT or nested case-control study data? | | Y |  |
| 1. Were all inclusions and exclusions of participants appropriate? | | NI |  |
| **Risk of bias introduced by selection of participants** | **RISK:**  *(low/ high/ unclear)* | **low** |  |
| *Rationale of bias rating: No Concerns* | | | |
|  | | | |
| **B. Applicability** | | | |
| *Describe included participants, setting and dates:*  It has been mentioned in Domain 1 – Section A. | | | |
| **Concern that the included participants and setting do not match the review question** | **CONCERN:**  *(low/ high/ unclear)* | **low** |  |
| *Rationale of applicability rating:* | | | |
| In our review, our source population is the general population. Therefore, this data is part of the general population. | | | |

| **DOMAIN 2:  Predictors** | | | |
| --- | --- | --- | --- |
| **A. Risk of Bias** | | | |
| *List and describe predictors included in the final model, e.g. definition and timing of assessment:*  18 out of 133 variables were identified as important predictor variables of COVID-19 hospitalization after the feature selection process. These variables are age, days since the last creatinine blood test, geographical latitude, days since the last basophils blood test, sex, number of family doctor visits in the last year, number of comorbidities, number of different unique subclasses of drugs taken in the last 2 years, highest value of creatinine recorded in the last 2 years, number of diagnostic radiology studies in the last 2 years, average value of neutrophils in blood in the last 2 years, number of doctor visits in the last 2 years, average value of leukocytes in blood in the last 2 years, number of creatinine blood tests in the last 2 years, highest value of hemoglobin recorded in the last 2 years, history of chronic kidney disease, and days since the last mean corpuscular hemoglobin test in the last 2 years.  To ensure that only the most recent data before the infection were included in the model, they included medical records dated no later than 30 days before the index date and not earlier than 2 years before the index date. The 2-year window was selected because most (> 92%) patients in the study cohort had at least 2 years of recorded clinical history in our database. The 30-day buffer before the index date was applied to ensure that only historical medical records were used to make a prediction for each patient and not tests done as a result of the SARS-CoV-2 infection. | | | |
|  | | Dev | Val |
| 1. Were predictors defined and assessed in a similar way for all participants? | | PY |  |
| 1. Were predictor assessments made without knowledge of outcome data? | | PY |  |
| 1. Are all predictors available at the time the model is intended to be used? | | Y |  |
| **Risk of bias introduced by predictors or their assessment** | **RISK:**  *(low/ high/ unclear)* | **low** |  |
| *Rationale of bias rating:* | | | |
| **B. Applicability** | | | |
| Concern that the definition, assessment or timing of predictors in the model do not match the review question | **CONCERN:**  *(low/ high/ unclear)* | **unclear** |  |
| *Rationale of applicability rating:*  *They used a 2 years covariant assessment window to capture the predictors which is not clearly defined and referenced.* | | | |

| **DOMAIN 3: Outcome** | | | |
| --- | --- | --- | --- |
| **A. Risk of Bias** | | | |
| *Describe the outcome, how it was defined and determined, and the time interval between predictor assessment and outcome determination:*  36 323 patients with 2583 hospitalizations (7.1%). | | | |
|  | | Dev | Val |
| 1. Was the outcome determined appropriately? | | PY |  |
| 1. Was a pre-specified or standard outcome definition used? | | PY |  |
| 1. Were predictors excluded from the outcome definition? | | Y |  |
| 1. Was the outcome defined and determined in a similar way for all participants? | | PY |  |
| 1. Was the outcome determined without knowledge of predictor information? | | PY |  |
| 1. Was the time interval between predictor assessment and outcome determination appropriate? | | PY |  |
| **Risk of bias introduced by the outcome or its determination** | **RISK:**  *(low/ high/ unclear)* | **low** |  |
| *Rationale of bias rating:* | | | |
| **B. Applicability** | | | |
| *At what time point was the outcome determined:*  *If a composite outcome was used, describe the relative frequency/distribution of each contributing outcome:* | | | |
| **Concern that the outcome, its definition, timing or determination do not match the review question** | **CONCERN:**  *(low/ high/ unclear)* | **low** |  |
| *Rationale of applicability rating:* | | | |
| **DOMAIN 4: Analysis** | | | |
| **Risk of Bias** | | | |
| *Describe numbers of participants, number of candidate predictors, outcome events and events per candidate predictor:*  DEV:  The cohort included 36 323 patients with 2583 hospitalizations (7.1%). Hospitalized patients had a higher median age (64 yr v. 43 yr), were more likely to be male (56.3% v. 47.3%) and had a higher median number of comorbidities (3, interquartile range [IQR] 2–6 v. 1, IQR 0–3) than nonhospitalized patients. | | | |
| *Describe how the model was developed (for example in regards to modelling technique (e.g. survival or logistic modelling), predictor selection, and risk group definition):*  Gradient-boosting decision trees, SHAP | | | |
| *Describe whether and how the model was validated, either internally (e.g. bootstrapping, cross validation, random split sample) or externally (e.g. temporal validation, geographical validation, different setting, different type of participants):*  Cross validation was used. | | | |
| *Describe the performance measures of the model, e.g. (re)calibration, discrimination, (re)classification, net benefit, and whether they were adjusted for optimism:*  The ROC analysis was performed. | | | |
| *Describe any participants who were excluded from the analysis:* | | | |
| *Describe missing data on predictors and outcomes as well as methods used for missing data:*  *Variables with records for less than 50% of the patients in the cohort were excluded.* | | | |
|  | | Dev | Val |
| 1. Were there a reasonable number of participants with the outcome? | | PY |  |
| 1. Were continuous and categorical predictors handled appropriately? | | PY |  |
| 1. Were all enrolled participants included in the analysis? | | PY |  |
| 1. Were participants with missing data handled appropriately? | | NI |  |
| 1. Was selection of predictors based on univariable analysis avoided? | | NI |  |
| 1. Were complexities in the data (e.g. censoring, competing risks, sampling of controls) accounted for appropriately? | | PY |  |
| 1. Were relevant model performance measures evaluated appropriately? | | PN |  |
| 1. Were model overfitting and optimism in model performance accounted for? | | PY |  |
| 1. Do predictors and their assigned weights in the final model correspond to the results from multivariable analysis? | | PY |  |
| **Risk of bias introduced by the analysis** | **RISK:**  *(low/ high/ unclear)* | **high** |  |
| *Rationale of bias rating:*  *Variables with records for less than 50% of the patients in the cohort were excluded.*  Linear models such as a logistic regression model was not used to get useful information regarding the significant relevant predictors which is familiar to clinical audiences.  The variable “geographical latitude” which was identified as a strong predictor, was obtained based on input data only from the region of Ontario. It can be correlated with sociodemographic factors, such as access to health care and income. Therefore the model maynot be generalized to other settings. | | | |

**Step 4: Overall assessment**

| | **Reaching an overall judgement about risk of bias of the prediction model evaluation** | | | --- | --- | | **Low risk of bias** | If all domains were rated low risk of bias.  If a prediction model was developed without any external validation, and it was rated as low risk of bias for all domains, consider downgrading to **high risk of bias**. Such a model can only be considered as low risk of bias, if the development was based on a very large data set and included some form of internal validation. | | **High risk of bias** | If at least one domain is judged to be at **high risk of bias**. | | **Unclear risk of bias** | If an unclear risk of bias was noted in at least one domain and it was low risk for all other domains. |  | **Reaching an overall judgement about applicability of the prediction model evaluation** | | | --- | --- | | **Low concerns regarding applicability** | If low concerns regarding applicability for all domains, the prediction model evaluation is judged to have **low concerns regarding applicability**. | | **High concerns regarding applicability** | If high concerns regarding applicability for at least one domain, the prediction model evaluation is judged to have **high concerns regarding applicability**. | | **Unclear concerns regarding applicability** | If unclear concerns (but no “high concern”) regarding applicability for at least one domain, the prediction model evaluation is judged to have **unclear concerns regarding applicability** overall. | |
| --- | --- | --- | --- | --- | --- | --- | --- | --- | --- | --- | --- | --- | --- | --- | --- | --- |

| **Overall judgement about risk of bias and applicability of the prediction model evaluation** | | |
| --- | --- | --- |
| **Overall judgement of risk of bias** | **RISK:**  *(low/ high/ unclear)* | **unclear** |
| *Summary of sources of potential bias:*  *They used a 2 years covariant assessment window to capture the predictors which is not clearly defined and referenced.*  *Variables with records for less than 50% of the patients in the cohort were excluded.*  Linear models such as a logistic regression model was not used to get useful information regarding the significant relevant predictors which is familiar to clinical audiences. | | |
| **Overall judgement of applicability** | **CONCERN:**  *(low/ high/ unclear)* | **high** |
| *Summary of applicability concerns:*  The variable “geographical latitude” which was identified as a strong predictor, was obtained based on input data only from the region of Ontario. It can be correlated with sociodemographic factors, such as access to health care and income. Therefore the model maynot be generalized to other settings. | | |

**Appendix 7**. Kasturi 2021 - Bias analysis using PROBAST.

**Step 1: Specify your systematic review question**

| **Criteria** | **Specify your systematic review question** |
| --- | --- |
| *Intended use of model:* | To inform the feasibility of leveraging broad, statewide datasets for population health–driven decision-making by developing robust analytical models that predict COVID-19–related health care resource utilization across patients served by Indiana’s statewide Health Information Exchange. |
| ***Participants*** *including selection criteria and setting:* | Positive tested COVID-19 patients |
| ***Predictors*** *(used in prediction modelling), including types of predictors (e.g. history, clinical examination, biochemical markers, imaging tests), time of measurement, specific measurement issues (e.g., any requirements/ prohibitions for specialized equipment):* | Demographics; diagnoses; past encounter history; medications; and social determinants of health, defined as conditions in which people are born, grow, live, work and age |
| *Outcome to be predicted:* | Hospitalization |

**Step 2: Classify the type of prediction model evaluation**

| **Classify the evaluation based on its aim** | | | |
| --- | --- | --- | --- |
| **Type of prediction study** | **PROBAST boxes to complete** | **Tick as appropriate** | **Definition for type of prediction model study** |
| Development only | Development | ✔ | Prediction model development without external validation. These studies may include internal validation methods, such as bootstrapping and cross-validation techniques. |
| Development and validation | Development and validation | ✖ | Prediction model development combined with external validation in other participants in the same article. |
| Validation only | Validation | ✖ | External validation of existing (previously developed) model in other participants. |

|  | |
| --- | --- |
| **Publication reference** | Kasturi SN, Park J, Wild D, Khan B, Haggstrom DA, Grannis S. Predicting COVID-19-Related Health Care Resource Utilization Across a Statewide Patient Population: Model Development Study. J Med Internet Res. 2021 Nov 15;23(11):e31337. doi: 10.2196/31337. PMID: 34581671; PMCID: PMC8594735. | |
| **Models of interest** | XGBoost |  |
| **Outcome of interest** | Hospitalization | |

**Step 3: Assess risk of bias and applicability**

| **DOMAIN 1:  Participants** | | | |
| --- | --- | --- | --- |
| **A. Risk of Bias** | | | |
| *Describe the sources of data and criteria for participant selection:*  DEV:  The COVID-19 Research Data Commons (CoRDaCo) dataset obtained from the Indiana Network for Patient Care. It consists of data from over 15 million inhabitants of Indiana spread across 23 health systems and 93 hospitals, as well as other state laboratory reporting state vitals data. Datasets included: demographics, diagnoses, past encounter history, medications, and social determinants of health between January 1, 2018, and November 30, 2020. | | | |
|  | | Dev | Val |
| 1. Were appropriate data sources used, e.g. cohort, RCT or nested case-control study data? | | Y |  |
| 1. Were all inclusions and exclusions of participants appropriate? | | NI |  |
| **Risk of bias introduced by selection of participants** | **RISK:**  *(low/ high/ unclear)* | **low** |  |
| *Rationale of bias rating: No Concerns* | | | |
|  | | | |
| **B. Applicability** | | | |
| *Describe included participants, setting and dates:*  It has been mentioned in Domain 1 – Section A. | | | |
| **Concern that the included participants and setting do not match the review question** | **CONCERN:**  *(low/ high/ unclear)* | **unclear** |  |
| *Rationale of applicability rating:* | | | |
| In our review, our source population is the general population. Therefore, this data is part of the general population. | | | |

| **DOMAIN 2:  Predictors** | | | |
| --- | --- | --- | --- |
| **A. Risk of Bias** | | | |
| *List and describe predictors included in the final model, e.g. definition and timing of assessment:*  age , chronic obstructive pulmonary disease status, smoking, diabetes, indication of neurological diseases via diagnosis (eg, dementia) or medications (eg, anti-Parkinson and related therapy agents), mental disorders (eg, anxiety disorders), residence (urban vs rural), and income-level, measured on the basis of the type of insurance used by the patient. | | | |
|  | | Dev | Val |
| 1. Were predictors defined and assessed in a similar way for all participants? | | PY |  |
| 1. Were predictor assessments made without knowledge of outcome data? | | PY |  |
| 1. Are all predictors available at the time the model is intended to be used? | | Y |  |
| **Risk of bias introduced by predictors or their assessment** | **RISK:**  *(low/ high/ unclear)* | **low** |  |
| *Rationale of bias rating: No Concerns* | | | |
| **B. Applicability** | | | |
| Concern that the definition, assessment or timing of predictors in the model do not match the review question | **CONCERN:**  *(low/ high/ unclear)* | **unclear** |  |
| *Rationale of applicability rating:*  *They used approximately  2-3 years of covariant assessment window to capture the predictors which is not clearly defined.* | | | |

| **DOMAIN 3: Outcome** | | | |
| --- | --- | --- | --- |
| **A. Risk of Bias** | | | |
| *Describe the outcome, how it was defined and determined, and the time interval between predictor assessment and outcome determination:*  Hospitalization was defined as patients who had been admitted to either inpatient or intensive care. two time intervals between prediction assessment and outcome were defined: The first week of receiving a diagnosis of COVID-19 (ie, 1-week cohort), including a measure of which patients were in need of urgent care at the time of, or soon after, diagnosis. The first 6 weeks of receiving a diagnosis of COVID-19 (ie, 6-week cohort). A metric of which patients would need inpatient care during the course of their illness | | | |
|  | | Dev | Val |
| 1. Was the outcome determined appropriately? | | PY |  |
| 1. Was a pre-specified or standard outcome definition used? | | PY |  |
| 1. Were predictors excluded from the outcome definition? | | Y |  |
| 1. Was the outcome defined and determined in a similar way for all participants? | | PY |  |
| 1. Was the outcome determined without knowledge of predictor information? | | PY |  |
| 1. Was the time interval between predictor assessment and outcome determination appropriate? | | PY |  |
| **Risk of bias introduced by the outcome or its determination** | **RISK:**  *(low/ high/ unclear)* | **low** |  |
| *Rationale of bias rating: No Concerns* | | | |
| **B. Applicability** | | | |
| *At what time point was the outcome determined:*  *If a composite outcome was used, describe the relative frequency/distribution of each contributing outcome:* | | | |
| **Concern that the outcome, its definition, timing or determination do not match the review question** | **CONCERN:**  *(low/ high/ unclear)* | **high** |  |
| *Rationale of applicability rating:*  The first week or the first 6 week excludes the needs of patients suffering long-COVID, where patients may not fully recover for several months | | | |
| **DOMAIN 4: Analysis** | | | |
| **Risk of Bias** | | | |
| *Describe numbers of participants, number of candidate predictors, outcome events and events per candidate predictor:*  DEV:  The dataset included 230,981 patients with a positive COVID-19 diagnosis but patients with some errors in their clinical and medical data were removed resulting in a total of 96,026.  A total of 18,694 (19.47%) and 22,678 (23.62%)  of these patients were hospitalized during the first week and the first 6 weeks of receiving a COVID-19 diagnosis respectively. | | | |
| *Describe how the model was developed (for example in regards to modelling technique (e.g. survival or logistic modelling), predictor selection, and risk group definition):*  XGBoost | | | |
| *Describe whether and how the model was validated, either internally (e.g. bootstrapping, cross validation, random split sample) or externally (e.g. temporal validation, geographical validation, different setting, different type of participants):*  Cross validation was used. | | | |
| *Describe the performance measures of the model, e.g. (re)calibration, discrimination, (re)classification, net benefit, and whether they were adjusted for optimism:*  The ROC analysis was performed. | | | |
| *Describe any participants who were excluded from the analysis:*  *To ensure that inpatient or intensive care unit stays influenced by COVID-19 alone, any admissions due to accidents such as falls, injuries, lacerations, and fractures, as well as suicidal ideation, overdoses, and alcohol abuse were excluded. These factors were selected for exclusion based on an assessment of the most frequently occurring admission reasons identified from patient hospitalization datasets.* | | | |
| *Describe missing data on predictors and outcomes as well as methods used for missing data:* | | | |
|  | | Dev | Val |
| 1. Were there a reasonable number of participants with the outcome? | | PY |  |
| 1. Were continuous and categorical predictors handled appropriately? | | PY |  |
| 1. Were all enrolled participants included in the analysis? | | PY |  |
| 1. Were participants with missing data handled appropriately? | | NI |  |
| 1. Was selection of predictors based on univariable analysis avoided? | | NI |  |
| 1. Were complexities in the data (e.g. censoring, competing risks, sampling of controls) accounted for appropriately? | | PY |  |
| 1. Were relevant model performance measures evaluated appropriately? | | PN |  |
| 1. Were model overfitting and optimism in model performance accounted for? | | PY |  |
| 1. Do predictors and their assigned weights in the final model correspond to the results from multivariable analysis? | | PY |  |
| **Risk of bias introduced by the analysis** | **RISK:**  *(low/ high/ unclear)* | **high** |  |
| *Rationale of bias rating:*  *The analysis was done during several waves of the COVID-19 pandemic when there were efforts to control COVID-19 infection rates. These might change health care systems resulting in changes in how many patients were provided inpatient care. This study did not consider how these variations influence the analysis. Moreover the model had not been validated with external datasets which limited its generalizability.* | | | |

**Step 4: Overall assessment**

| | **Reaching an overall judgement about risk of bias of the prediction model evaluation** | | | --- | --- | | **Low risk of bias** | If all domains were rated low risk of bias.  If a prediction model was developed without any external validation, and it was rated as low risk of bias for all domains, consider downgrading to **high risk of bias**. Such a model can only be considered as low risk of bias, if the development was based on a very large data set and included some form of internal validation. | | **High risk of bias** | If at least one domain is judged to be at **high risk of bias**. | | **Unclear risk of bias** | If an unclear risk of bias was noted in at least one domain and it was low risk for all other domains. |  | **Reaching an overall judgement about applicability of the prediction model evaluation** | | | --- | --- | | **Low concerns regarding applicability** | If low concerns regarding applicability for all domains, the prediction model evaluation is judged to have **low concerns regarding applicability**. | | **High concerns regarding applicability** | If high concerns regarding applicability for at least one domain, the prediction model evaluation is judged to have **high concerns regarding applicability**. | | **Unclear concerns regarding applicability** | If unclear concerns (but no “high concern”) regarding applicability for at least one domain, the prediction model evaluation is judged to have **unclear concerns regarding applicability** overall. | |
| --- | --- | --- | --- | --- | --- | --- | --- | --- | --- | --- | --- | --- | --- | --- | --- | --- |

| **Overall judgement about risk of bias and applicability of the prediction model evaluation** | | |
| --- | --- | --- |
| **Overall judgement of risk of bias** | **RISK:**  *(low/ high/ unclear)* | **low** |
| *Summary of sources of potential bias:* | | |
| **Overall judgement of applicability** | **CONCERN:**  *(low/ high/ unclear)* | **high** |
| *Summary of applicability concerns:*  *They used approximately  2-3 years of covariant assessment window to capture the predictors which is not clearly defined.*  the first week or the first 6 week excludes the needs of patients suffering long-COVID, where patients may not fully recover for several months  *The analysis was done during several waves of the COVID-19 pandemic when there were efforts to control COVID-19 infection rates. These might change health care systems resulting in changes in how many patients were provided inpatient care. This study did not consider how these variations influence the analysis. Moreover the model had not been validated with external datasets which limited its generalizability.* | | |

**Appendix 8**. Polilli 2022 - Bias analysis using PROBAST.

**Step 1: Specify your systematic review question**

| **Criteria** | **Specify your systematic review question** |
| --- | --- |
| *Intended use of model:* | To identify patients positive to COVID-19 who are at risk of hospitalization or death. |
| ***Participants*** *including selection criteria and setting:* | Patients positive to COVID-19. |
| ***Predictors*** *(used in prediction modelling), including types of predictors (e.g. history, clinical examination, biochemical markers, imaging tests), time of measurement, specific measurement issues (e.g., any requirements/ prohibitions for specialized equipment):* | Sociodemographic and clinical characteristics of patients, drug exposure, and biochemical/immunological markers. |
| *Outcome to be predicted:* | Hospitalization or death following COVID-19 diagnosis. |

**Step 2: Classify the type of prediction model evaluation**

| **Classify the evaluation based on its aim** | | | |
| --- | --- | --- | --- |
| **Type of prediction study** | **PROBAST boxes to complete** | **Tick as appropriate** | **Definition for type of prediction model study** |
| Development only | Development | ✖ | Prediction model development without external validation. These studies may include internal validation methods, such as bootstrapping and cross-validation techniques. |
| Development and validation | Development and validation | ✔ | Prediction model development combined with external validation in other participants in the same article. |
| Validation only | Validation | ✖ | External validation of existing (previously developed) model in other participants. |

|  | |
| --- | --- |
| **Publication reference** | Polilli E, Frattari A, Esposito JE, D'Amato M, Rapacchiale G, D'Intino A, Albani A, Di Iorio G, Carinci F, Parruti G. Reliability of predictive models to support early decision making in the emergency department for patients with confirmed diagnosis of COVID-19: the Pescara Covid Hospital score. BMC Health Serv Res. 2022 Aug 19;22(1):1062. doi: 10.1186/s12913-022-08421-4. PMID: 35986291; PMCID: PMC9390116. | |
| **Models of interest** | Logistic regression and Cox proportional hazard model | |
| **Outcome of interest** | Hospitalization and Mortality (combined) | |

**Step 3: Assess risk of bias and applicability**

| **DOMAIN 1:  Participants** | | | |
| --- | --- | --- | --- |
| **A. Risk of Bias** | | | |
| *Describe the sources of data and criteria for participant selection:*  DEV: All consecutive patients presented with a confirmed diagnosis of COVID-19 at the emergency department of the General Hospital of Pescara (Abruzzo, Italy), between 1st March – 30th June 2020. The diagnosis was confirmed through a swab test performed by the hospital personnel in the same occasion. Data were merged from records available at different sources, including the hospital discharge abstract database, the computerized hospital information system including personal health records of laboratory measurements, and paper-based clinical abstracts, from which other characteristics were manually extracted.  VAL: Two additional samples were collected for external validation, using a large subset of entries to the same emergency department between December 2020–January 2021 (corresponding to the transition from the “Wild” lineage of COVID-19 to the “Alpha” variant) and January–March 2022 (covering the transition between the “Delta” variant and “Omicron”). | | | |
|  | | Dev | Val |
| 1. Were appropriate data sources used, e.g. cohort, RCT or nested case-control study data? | | Y | PY |
| 1. Were all inclusions and exclusions of participants appropriate? | | PN | PN |
| **Risk of bias introduced by selection of participants** | **RISK:**  *(low/ high/ unclear)* | **high** | **high** |
| *Rationale of bias rating:* | | | |
| There is a major concern related to the inclusion criteria. The authors included all patients admitted to the emergency department with a positive COVID-19 diagnosis but they did not investigate the cause of access to the emergency department. Therefore, patients may have been positive but admitted to the emergency department for reasons other than COVID-19. Therefore, predictors of hospitalization and mortality may capture risk factors for highly prevalent causes of emergency department access rather than COVID-19-related risk factors. Additionally, we are not aware of the validity of the COVID-19 diagnosis at the aforementioned emergency department. Finally, data from the Pescara General Hospital only contains information on adults admitted for trauma, acute diseases of neurosurgical interest, or COVID-19, which increases the risk of selection bias and lack of representation with the source population. Both development and validation of models have been performed using data collected in a single care unit (the same for development and validation). | | | |
| **B. Applicability** | | | |
| *Describe included participants, setting and dates:*  It has been mentioned in Domain 1 – Section A. | | | |
| **Concern that the included participants and setting do not match the review question** | **CONCERN:**  *(low/ high/ unclear)* | **low** | **low** |
| *Rationale of applicability rating:* | | | |
| In our review, our source population is the general population. Therefore, we did not restrict the source population to individuals admitted to emergency department. Consequently, individuals admitted to the the emergency department are part of the general population. | | | |

| **DOMAIN 2:  Predictors** | | | |
| --- | --- | --- | --- |
| **A. Risk of Bias** | | | |
| *List and describe predictors included in the final model, e.g. definition and timing of assessment:*  Age, gender, diabetes, cardiovascular diseases, obesity, cancer, end stage renal disease, chronic obstructive pulmonary disease, hypertension, signs and symptoms related to COVID-19 (e.g. fever, cough, asthenia, diarrhoea, and dyspnoea), procalcitonin, lactate dehydrogenase, monocyte distribution width, oxygen saturation level, D-dimer, prothrombin time, C-reactive Protein, and lymphocyte counts. | | | |
|  | | Dev | Val |
| 1. Were predictors defined and assessed in a similar way for all participants? | | Y | Y |
| 1. Were predictor assessments made without knowledge of outcome data? | | Y | Y |
| 1. Are all predictors available at the time the model is intended to be used? | | Y | Y |
| **Risk of bias introduced by predictors or their assessment** | **RISK:**  *(low/ high/ unclear)* | **low** | **low** |
| *Rationale of bias rating:* | | | |
| **B. Applicability** | | | |
| Concern that the definition, assessment or timing of predictors in the model do not match the review question | **CONCERN:**  *(low/ high/ unclear)* | **low** | **low** |
| *Rationale of applicability rating:*  Predictors were assessed prior to the occurrence of the outcome. Therefore, all predictors included in this article can be considered valid. | | | |

| **DOMAIN 3: Outcome** | | | |
| --- | --- | --- | --- |
| **A. Risk of Bias** | | | |
| *Describe the outcome, how it was defined and determined, and the time interval between predictor assessment and outcome determination:*  We investigated hospitalisation and death in/out of hospital through follow-up of all patients in the study population. | | | |
|  | | Dev | Val |
| 1. Was the outcome determined appropriately? | | PY | PY |
| 1. Was a pre-specified or standard outcome definition used? | | PY | PN |
| 1. Were predictors excluded from the outcome definition? | | Y | Y |
| 1. Was the outcome defined and determined in a similar way for all participants? | | Y | Y |
| 1. Was the outcome determined without knowledge of predictor information? | | PY | PY |
| 1. Was the time interval between predictor assessment and outcome determination appropriate? | | NI | NI |
| **Risk of bias introduced by the outcome or its determination** | **RISK:**  *(low/ high/ unclear)* | **high** | **high** |
| *Rationale of bias rating:*  We cannot excluded other aetiology for death after the patient left the hospital. The authors did not provide a definition of hospitalization. There are no information regarding the time interval for the assessment of the predictors. | | | |
| **B. Applicability** | | | |
| *At what time point was the outcome determined:*  There are no information regarding the time interval for the assessment of the outcome.  *If a composite outcome was used, describe the relative frequency/distribution of each contributing outcome:* | | | |
| **Concern that the outcome, its definition, timing or determination do not match the review question** | **CONCERN:**  *(low/ high/ unclear)* | **high** | **high** |
| *Rationale of applicability rating: mentioned in above.* | | | |

| **DOMAIN 4: Analysis** | | | |
| --- | --- | --- | --- |
| **Risk of Bias** | | | |
| *Describe numbers of participants, number of candidate predictors, outcome events and events per candidate predictor:*  DEV: 536 participants, 10 candidate predictors  VAL: 579 participants and 10 candidate predictors  365 hospitalizations, 400 deaths | | | |
| *Describe how the model was developed (for example in regards to modelling technique (e.g. survival or logistic modelling), predictor selection, and risk group definition):*  Logistic regression was used for univariate and multivariate odds ratios of hospitalization. Survival analysis was used to consider censoring in the analysis of time to fatal events, calculated as the difference between the date of death and admission to the hospital. For survivors, the censoring time was defined as the lag between presentation at the emergency department and the earliest date between the first negative swab result (an indicator of full recovery) and the date of study closure (30th June 2020). Cox proportional hazards was used for the calculation of univariate and multivariate hazard ratios (HR) for time-to-event analysis. An alpha level of 0.05 was used to present odds and hazard ratios together with their 95% confidence intervals (95%CI) and p values. Forest plots were used to visualize results.  Predictive factors using a fully automated four-step backward elimination process in all multivariate regressions. Age and gender were forced in all models, with all other variables sequentially excluded in three consecutive rounds using a p value ≥0.20, ≥0.10 and ≥ 0.05. | | | |
| *Describe whether and how the model was validated, either internally (e.g. bootstrapping, cross validation, random split sample) or externally (e.g. temporal validation, geographical validation, different setting, different type of participants):*  Not available. | | | |
| *Describe the performance measures of the model, e.g. (re)calibration, discrimination, (re)classification, net benefit, and whether they were adjusted for optimism:*  A ROC analysis was performed using a separate 2 × 2 “confusion matrix” for every possible threshold applied to the total score. The best threshold was defined by the highest value of the Youden Index. Point estimates and confidence intervals were computed using the bootstrap for sensitivity, specificity, Positive Predictive Value (PPV), Negative Predictive Value (NPV) and the De Long method for the Area Under the Curve (AUC)  Reliability analysis of the predictive formula was summarized using ROC curves showing optimal thresholds and sensitivity, specificity, AUC with point estimates and 95% confidence intervals. | | | |
| *Describe any participants who were excluded from the analysis:*  Not available. | | | |
| *Describe missing data on predictors and outcomes as well as methods used for missing data:*  Not available. | | | |
|  | | Dev | Val |
| 1. Were there a reasonable number of participants with the outcome? | | NI | NI |
| 1. Were continuous and categorical predictors handled appropriately? | | PY | PY |
| 1. Were all enrolled participants included in the analysis? | | PY | PY |
| 1. Were participants with missing data handled appropriately? | | NI | NI |
| 1. Was selection of predictors based on univariable analysis avoided? | | Y |  |
| 1. Were complexities in the data (e.g. censoring, competing risks, sampling of controls) accounted for appropriately? | | Y |  |
| 1. Were relevant model performance measures evaluated appropriately? | | Y | Y |
| 1. Were model overfitting and optimism in model performance accounted for? | | N |  |
| 1. Do predictors and their assigned weights in the final model correspond to the results from multivariable analysis? | | NI |  |
| **Risk of bias introduced by the analysis** | **RISK:**  *(low/ high/ unclear)* | **high** |  |
| *Rationale of bias rating:*  We lack information on the number of patients with the outcome separately for the development and validation models. We assume that they used the same predictors in the development and validation models.  They do not describe how they handled missing data. | | | |

**Step 4: Overall assessment**

| | **Reaching an overall judgement about risk of bias of the prediction model evaluation** | | | --- | --- | | **Low risk of bias** | If all domains were rated low risk of bias.  If a prediction model was developed without any external validation, and it was rated as low risk of bias for all domains, consider downgrading to **high risk of bias**. Such a model can only be considered as low risk of bias, if the development was based on a very large data set and included some form of internal validation. | | **High risk of bias** | If at least one domain is judged to be at **high risk of bias**. | | **Unclear risk of bias** | If an unclear risk of bias was noted in at least one domain and it was low risk for all other domains. |  | **Reaching an overall judgement about applicability of the prediction model evaluation** | | | --- | --- | | **Low concerns regarding applicability** | If low concerns regarding applicability for all domains, the prediction model evaluation is judged to have **low concerns regarding applicability**. | | **High concerns regarding applicability** | If high concerns regarding applicability for at least one domain, the prediction model evaluation is judged to have **high concerns regarding applicability**. | | **Unclear concerns regarding applicability** | If unclear concerns (but no “high concern”) regarding applicability for at least one domain, the prediction model evaluation is judged to have **unclear concerns regarding applicability** overall. | |
| --- | --- | --- | --- | --- | --- | --- | --- | --- | --- | --- | --- | --- | --- | --- | --- | --- |

| **Overall judgement about risk of bias and applicability of the prediction model evaluation** | | |
| --- | --- | --- |
| **Overall judgement of risk of bias** | **RISK:**  *(low/ high/ unclear)* | **high** |
| *Summary of sources of potential bias:* | | |
| **Overall judgement of applicability** | **CONCERN:**  *(low/ high/ unclear)* | **high** |
| *Summary of applicability concerns:* | | |

**Appendix 9**. Jimenez 2022 - Bias analysis using PROBAST.

**Step 1: Specify your systematic review question**

| **Criteria** | **Specify your systematic review question** |
| --- | --- |
| *Intended use of model:* | To predict risks at different stages of management and thereby provide insights into drivers and prognostic markers of disease progression and death. |
| ***Participants*** *including selection criteria and setting:* | Positive tested COVID-19 patients |
| ***Predictors*** *(used in prediction modelling), including types of predictors (e.g. history, clinical examination, biochemical markers, imaging tests), time of measurement, specific measurement issues (e.g., any requirements/ prohibitions for specialized equipment):* | Demographics, comorbidities and prescription medication. In-hospital data included laboratory results and vital signs |
| *Outcome to be predicted:* | Mortality and hospitalization |

**Step 2: Classify the type of prediction model evaluation**

| **Classify the evaluation based on its aim** | | | |
| --- | --- | --- | --- |
| **Type of prediction study** | **PROBAST boxes to complete** | **Tick as appropriate** | **Definition for type of prediction model study** |
| Development only | Development | ✖ | Prediction model development without external validation. These studies may include internal validation methods, such as bootstrapping and cross-validation techniques. |
| Development and validation | Development and validation | ✔ | Prediction model development combined with external validation in other participants in the same article. |
| Validation only | Validation | ✖ | External validation of existing (previously developed) model in other participants. |

|  | |
| --- | --- |
| **Publication reference** | Jimenez-Solem E, Petersen TS, Hansen C, Hansen C, Lioma C, Igel C, Boomsma W, Krause O, Lorenzen S, Selvan R, Petersen J, Nyeland ME, Ankarfeldt MZ, Virenfeldt GM, Winther-Jensen M, Linneberg A, Ghazi MM, Detlefsen N, Lauritzen AD, Smith AG, de Bruijne M, Ibragimov B, Petersen J, Lillholm M, Middleton J, Mogensen SH, Thorsen-Meyer HC, Perner A, Helleberg M, Kaas-Hansen BS, Bonde M, Bonde A, Pai A, Nielsen M, Sillesen M. Developing and validating COVID-19 adverse outcome risk prediction models from a bi-national European cohort of 5594 patients. Sci Rep. 2021 Feb 5;11(1):3246. doi: 10.1038/s41598-021-81844-x. PMID: 33547335; PMCID: PMC7864944. | |
| **Models of interest** | Random Forest |  |
| **Outcome of interest** | Mortality and Hospitalization | |

**Step 3: Assess risk of bias and applicability**

| **DOMAIN 1:  Participants** | | | |
| --- | --- | --- | --- |
| **A. Risk of Bias** | | | |
| *Describe the sources of data and criteria for participant selection:*  DEV: The study included  information of 3944 Covid-19 patients (1359 (34.5%) required hospitalization, 181 (4.6%) intensive care, and 324 deaths(8.2%)) in Denmark between March 1st, 2020 and June 16th 2020 which were obtained from electronic health records (EHRs) with nationwide medical registry data.  Val: For external validation data from the UK biobank including 1650 COVID-19 patients was used. | | | |
|  | | Dev | Val |
| 1. Were appropriate data sources used, e.g. cohort, RCT or nested case-control study data? | | Y | Y |
| 1. Were all inclusions and exclusions of participants appropriate? | | NI | NI |
| **Risk of bias introduced by selection of participants** | **RISK:**  *(low/ high/ unclear)* | **unclear** | **unclear** |
| *Rationale of bias rating:* | | | |
| No information found for inclusions and exclusions of participants. | | | |
| **B. Applicability** | | | |
| *Describe included participants, setting and dates:*  It has been mentioned in Domain 1 – Section A. | | | |
| **Concern that the included participants and setting do not match the review question** | **CONCERN:**  *(low/ high/ unclear)* | **unclear** | **unclear** |
| *Rationale of applicability rating:* | | | |
| The dataset that was used included a small number of patients which is not enough for the prediction model development since additional patient data could change the results. | | | |

| **DOMAIN 2:  Predictors** | | | |
| --- | --- | --- | --- |
| **A. Risk of Bias** | | | |
| *List and describe predictors included in the final model, e.g. definition and timing of assessment:*  Dev: The Danish dataset included demographics, comorbidities, prescription medication, laboratory results and vital signs.  val: only demographics, comorbidities were available on the UK biobank dataset. | | | |
|  | | Dev | Val |
| 1. Were predictors defined and assessed in a similar way for all participants? | | PY | PY |
| 1. Were predictor assessments made without knowledge of outcome data? | | PY | PY |
| 1. Are all predictors available at the time the model is intended to be used? | | NI | NI |
| **Risk of bias introduced by predictors or their assessment** | **RISK:**  *(low/ high/ unclear)* | **unclear** | **unclear** |
| *Rationale of bias rating:*  They did not use the covariant assessment window for variable generation | | | |
| **B. Applicability** | | | |
| Concern that the definition, assessment or timing of predictors in the model do not match the review question | **CONCERN:**  *(low/ high/ unclear)* | **high** | **high** |
| *Rationale of applicability rating:*    They used a subset of clinical variables from the EHR system and analysing other features could affect the model. Furthermore, the analysis was done during Covid-19 pandemic that could affected the results, for example criteria for SARS-CoV-2 testing. | | | |

| **DOMAIN 3: Outcome** | | | |
| --- | --- | --- | --- |
| **A. Risk of Bias** | | | |
| *Describe the outcome, how it was defined and determined, and the time interval between predictor assessment and outcome determination:*  The study included  information of 3944 Covid-19 patients (1359 (34.5%) required hospitalization, 181 (4.6%) intensive care, and 324 deaths(8.2%)) in Denmark between March 1st, 2020 and June 16th 2020 .  For external validation data from the UK biobank including 1650 COVID-19 patients was used. | | | |
|  | | Dev | Val |
| 1. Was the outcome determined appropriately? | | PY | PY |
| 1. Was a pre-specified or standard outcome definition used? | | PY | PY |
| 1. Were predictors excluded from the outcome definition? | | Y | Y |
| 1. Was the outcome defined and determined in a similar way for all participants? | | PY | PY |
| 1. Was the outcome determined without knowledge of predictor information? | | PY | PY |
| 1. Was the time interval between predictor assessment and outcome determination appropriate? | | NI | NI |
| **Risk of bias introduced by the outcome or its determination** | **RISK:**  *(low/ high/ unclear)* | **unclear** | **unclear** |
| *Rationale of bias rating:*  *The time interval between admission time and outcome was not defined.* | | | |
| **B. Applicability** | | | |
| *At what time point was the outcome determined:*  *If a composite outcome was used, describe the relative frequency/distribution of each contributing outcome:* | | | |
| **Concern that the outcome, its definition, timing or determination do not match the review question** | **CONCERN:**  *(low/ high/ unclear)* | **high** | **high** |
| *Rationale of applicability rating:*  There was no information regarding discharged patients after they left the hospital, they might have died shortly.  In-hospital models could not be externally validated due to lack of availability of these data points in the UK biobank. | | | |
| **DOMAIN 4: Analysis** | | | |
| **Risk of Bias** | | | |
| *Describe numbers of participants, number of candidate predictors, outcome events and events per candidate predictor:*  DEV:   The study included  information of 3944 Covid-19 patients (1359 (34.5%) required hospitalization, 181 (4.6%) intensive care, and 324 deaths(8.2%))with about 50 predictors  For external validation data from the UK biobank including 1650 COVID-19 patients was used. | | | |
| *Describe how the model was developed (for example in regards to modelling technique (e.g. survival or logistic modelling), predictor selection, and risk group definition):*  Random Forest. | | | |
| *Describe whether and how the model was validated, either internally (e.g. bootstrapping, cross validation, random split sample) or externally (e.g. temporal validation, geographical validation, different setting, different type of participants):*  Cross validation was  used. | | | |
| *Describe the performance measures of the model, e.g. (re)calibration, discrimination, (re)classification, net benefit, and whether they were adjusted for optimism:*  The ROC analysis was performed. | | | |
| *Describe any participants who were excluded from the analysis:*  *NI* | | | |
| *Describe missing data on predictors and outcomes as well as methods used for missing data:*  *Missing values for BMI were imputed by using k-nearest neighbour imputation using age and sex*[*17*](https://www.ncbi.nlm.nih.gov/pmc/articles/PMC7864944/#CR17)*, with k = 100. Other missing data points were set to “not available” for the purpose of ML modelling and deleted by case wise deletion for group comparisons.* | | | |
|  | | Dev | Val |
| 1. Were there a reasonable number of participants with the outcome? | | N | N |
| 1. Were continuous and categorical predictors handled appropriately? | | PY | PY |
| 1. Were all enrolled participants included in the analysis? | | PY | PY |
| 1. Were participants with missing data handled appropriately? | | PY | PY |
| 1. Was selection of predictors based on univariable analysis avoided? | | PY | PY |
| 1. Were complexities in the data (e.g. censoring, competing risks, sampling of controls) accounted for appropriately? | | PY | PY |
| 1. Were relevant model performance measures evaluated appropriately? | | PN | PN |
| 1. Were model overfitting and optimism in model performance accounted for? | | PY | PY |
| 1. Do predictors and their assigned weights in the final model correspond to the results from multivariable analysis? | | PY | PY |
| **Risk of bias introduced by the analysis** | **RISK:**  *(low/ high/ unclear)* | **unclear** | **unclear** |
| *Rationale of bias rating:*  The study suffered from a low number of participants both for development and validation. The model did not perform as well on external validation data with much lower auc value. | | | |

**Step 4: Overall assessment**

| | **Reaching an overall judgement about risk of bias of the prediction model evaluation** | | | --- | --- | | **Low risk of bias** | If all domains were rated low risk of bias.  If a prediction model was developed without any external validation, and it was rated as low risk of bias for all domains, consider downgrading to **high risk of bias**. Such a model can only be considered as low risk of bias, if the development was based on a very large data set and included some form of internal validation. | | **High risk of bias** | If at least one domain is judged to be at **high risk of bias**. | | **Unclear risk of bias** | If an unclear risk of bias was noted in at least one domain and it was low risk for all other domains. |  | **Reaching an overall judgement about applicability of the prediction model evaluation** | | | --- | --- | | **Low concerns regarding applicability** | If low concerns regarding applicability for all domains, the prediction model evaluation is judged to have **low concerns regarding applicability**. | | **High concerns regarding applicability** | If high concerns regarding applicability for at least one domain, the prediction model evaluation is judged to have **high concerns regarding applicability**. | | **Unclear concerns regarding applicability** | If unclear concerns (but no “high concern”) regarding applicability for at least one domain, the prediction model evaluation is judged to have **unclear concerns regarding applicability** overall. | |
| --- | --- | --- | --- | --- | --- | --- | --- | --- | --- | --- | --- | --- | --- | --- | --- | --- |

| **Overall judgement about risk of bias and applicability of the prediction model evaluation** | | |
| --- | --- | --- |
| **Overall judgement of risk of bias** | **RISK:**  *(low/ high/ unclear)* | **unclear** |
| *Summary of sources of potential bias:*  *The time interval between admission time and outcome was not defined.* No information found for inclusions and exclusions of participants. They did not use the covariant assessment window for variable generation | | |
| **Overall judgement of applicability** | **CONCERN:**  *(low/ high/ unclear)* | **high** |
| *Summary of applicability concerns:*  The dataset that was used included a small number of patients which is not enough for the prediction model development since additional patient data could change the results.  They used a subset of clinical variables from the EHR system and analysing other features could affect the model. Furthermore, the analysis was done during Covid-19 pandemic that could affected the results, for example criteria for SARS-CoV-2 testing.  There was no information regarding discharged patients after they left the hospital, they might have died shortly.  In-hospital models could not be externally validated due to lack of availability of these data points in the UK biobank.  The study suffered from a reasonable number of participants both for development and validation. The model did not perform as well on external validation data with much lower auc value. | | |

**Appendix 10**. < 2020 - Bias analysis using PROBAST.

**Step 1: Specify your systematic review question**

| **Criteria** | **Specify your systematic review question** |
| --- | --- |
| *Intended use of model:* | To develop machine learning models for making predictions about the hospital course of the patients over clinically meaningful time horizons based on patient characteristics at admission |
| ***Participants*** *including selection criteria and setting:* | positive tested COVID-19 patients and hospitalized |
| ***Predictors*** *(used in prediction modelling), including types of predictors (e.g. history, clinical examination, biochemical markers, imaging tests), time of measurement, specific measurement issues (e.g., any requirements/ prohibitions for specialized equipment):* | demographics, past medical history, and admission vital signs and laboratory test results |
| *Outcome to be predicted:* | Mortality and hospitalization |

**Step 2: Classify the type of prediction model evaluation**

| **Classify the evaluation based on its aim** | | | |
| --- | --- | --- | --- |
| **Type of prediction study** | **PROBAST boxes to complete** | **Tick as appropriate** | **Definition for type of prediction model study** |
| Development only | Development | ✖ | Prediction model development without external validation. These studies may include internal validation methods, such as bootstrapping and cross-validation techniques. |
| Development and validation | Development and validation | ✔ | Prediction model development combined with external validation in other participants in the same article. |
| Validation only | Validation | ✖ | External validation of existing (previously developed) model in other participants. |

|  | |
| --- | --- |
| **Publication reference** | Vaid A, Somani S, Russak AJ, De Freitas JK, Chaudhry FF, Paranjpe I, Johnson KW, Lee SJ, Miotto R, Richter F, Zhao S, Beckmann ND, Naik N, Kia A, Timsina P, Lala A, Paranjpe M, Golden E, Danieletto M, Singh M, Meyer D, O'Reilly PF, Huckins L, Kovatch P, Finkelstein J, Freeman RM, Argulian E, Kasarskis A, Percha B, Aberg JA, Bagiella E, Horowitz CR, Murphy B, Nestler EJ, Schadt EE, Cho JH, Cordon-Cardo C, Fuster V, Charney DS, Reich DL, Bottinger EP, Levin MA, Narula J, Fayad ZA, Just AC, Charney AW, Nadkarni GN, Glicksberg BS. Machine Learning to Predict Mortality and Critical Events in a Cohort of Patients With COVID-19 in New York City: Model Development and Validation. J Med Internet Res. 2020 Nov 6;22(11):e24018. doi: 10.2196/24018. PMID: 33027032; PMCID: PMC7652593. | |
| **Models of interest** | Extreme Gradient Boosting (XGBoost),  logistic regression, KNN |  |
| **Outcome of interest** | Mortality and Hospitalization | |

**Step 3: Assess risk of bias and applicability**

| **DOMAIN 1:  Participants** | | | |
| --- | --- | --- | --- |
| **A. Risk of Bias** | | | |
| *Describe the sources of data and criteria for participant selection:*  The study included EHRs records of 4098 COVID-19–positive patients admitted to five hospitals in New York City from March 15 to May 22, 2020.  DEV and val: The models were first trained on patients from a single hospital (n=1514) before or on May 1, externally validated on patients from four other hospitals (n=2201) before or on May 1, and prospectively validated on all patients after May 1 (n=383). | | | |
|  | | Dev | Val |
| 1. Were appropriate data sources used, e.g. cohort, RCT or nested case-control study data? | | Y | Y |
| 1. Were all inclusions and exclusions of participants appropriate? | | Y | Y |
| **Risk of bias introduced by selection of participants** | **RISK:**  *(low/ high/ unclear)* | **low** | **low** |
| *Rationale of bias rating: No Concerns* | | | |
|  | | | |
| **B. Applicability** | | | |
| *Describe included participants, setting and dates:*  It has been mentioned in Domain 1 – Section A. | | | |
| **Concern that the included participants and setting do not match the review question** | **CONCERN:**  *(low/ high/ unclear)* | **high** | **high** |
| *Rationale of applicability rating:* | | | |
| The study was conducted in a short period from March 15 to May 22, 2020 and included a small number of patients which is not enough for the prediction model development since additional patient data with different periods could change the results. | | | |

| **DOMAIN 2:  Predictors** | | | |
| --- | --- | --- | --- |
| **A. Risk of Bias** | | | |
| *List and describe predictors included in the final model, e.g. definition and timing of assessment:*  Dev: The dataset included variables from demographics, past medical history, and admission vital signs and laboratory test results of patients.  *timing of assessments* were constructed at 3-, 5-, 7-, and 10-day intervals after admission | | | |
|  | | Dev | Val |
| 1. Were predictors defined and assessed in a similar way for all participants? | | PY | PY |
| 1. Were predictor assessments made without knowledge of outcome data? | | PY | PY |
| 1. Are all predictors available at the time the model is intended to be used? | | NI | NI |
| **Risk of bias introduced by predictors or their assessment** | **RISK:**  *(low/ high/ unclear)* | **unclear** | **unclear** |
| *Rationale of bias rating:*  Predictions solely on data extracted around patient admission (ie, within 36 hours). | | | |
| **B. Applicability** | | | |
| Concern that the definition, assessment or timing of predictors in the model do not match the review question | **CONCERN:**  *(low/ high/ unclear)* | **high** | **high** |
| *Rationale of applicability rating:*They used a subset of clinical variables from the EHR system and analysing other features could affect the model. Furthermore, the analysis was done during Covid-19 pandemic that could affected the results, for example criteria for SARS-CoV-2 testing. | | | |

| **DOMAIN 3: Outcome** | | | |
| --- | --- | --- | --- |
| **A. Risk of Bias** | | | |
| *Describe the outcome, how it was defined and determined, and the time interval between predictor assessment and outcome determination:*  The two primary outcomes were (1) death versus survival or discharge and (2) critical illness versus survival or discharge through time horizons of 3, 5, 7, and 10 days. Critical illness was defined as discharge to hospice, intubation ≤48 hours prior to intensive care unit (ICU) admission, ICU admission, or death. A composite outcome (ie, mortality as opposed to discharge or survival) was chosen to bypass issues of competing risks.  Before May 1, 21.3% to 35.3% of patients had experienced a critical event (intubation, ICU admission, discharge to hospice care, or death) across all time intervals. On or after May 1, this proportion changed to 14.3% to 21.9%. Similarly, before May 1, 2.6% to 22.4% patients died across all time intervals, with the proportion changing to 1.1% to 8.0% on or after May 1. | | | |
|  | | Dev | Val |
| 1. Was the outcome determined appropriately? | | PY | PY |
| 1. Was a pre-specified or standard outcome definition used? | | PY | PY |
| 1. Were predictors excluded from the outcome definition? | | Y | Y |
| 1. Was the outcome defined and determined in a similar way for all participants? | | PY | PY |
| 1. Was the outcome determined without knowledge of predictor information? | | PY | PY |
| 1. Was the time interval between predictor assessment and outcome determination appropriate? | | NI | NI |
| **Risk of bias introduced by the outcome or its determination** | **RISK:**  *(low/ high/ unclear)* | **unclear** | **unclear** |
| *Rationale of bias rating:*  *There was no reference of whether the* time interval between predictor assessment and outcome determined *appropriate or not.* | | | |
| **B. Applicability** | | | |
| *At what time point was the outcome determined:*  *If a composite outcome was used, describe the relative frequency/distribution of each contributing outcome:* | | | |
| **Concern that the outcome, its definition, timing or determination do not match the review question** | **CONCERN:**  *(low/ high/ unclear)* | **high** | **high** |
| *Rationale of applicability rating:*  Patients admitted to the hospital later in the crisis benefited from improved patient care protocols from experiential learning but were also negatively affected by resource constraints from overburdened hospitals. These effects may also induce temporal variation between patient outcomes, which is demonstrated by the lower critical event and mortality rate in the prospective validation data set. | | | |
| **DOMAIN 4: Analysis** | | | |
| **Risk of Bias** | | | |
| *Describe numbers of participants, number of candidate predictors, outcome events and events per candidate predictor:*  DEV:   The two primary outcomes were (1) death versus survival or discharge and (2) critical illness versus survival or discharge through time horizons of 3, 5, 7, and 10 days. Critical illness was defined as discharge to hospice, intubation ≤48 hours prior to intensive care unit (ICU) admission, ICU admission, or death. A composite outcome (ie, mortality as opposed to discharge or survival) was chosen to bypass issues of competing risks.  Before May 1, 21.3% to 35.3% of patients had experienced a critical event (intubation, ICU admission, discharge to hospice care, or death) across all time intervals. On or after May 1, this proportion changed to 14.3% to 21.9%. Similarly, before May 1, 2.6% to 22.4% patients died across all time intervals, with the proportion changing to 1.1% to 8.0% on or after May 1. | | | |
| *Describe how the model was developed (for example in regards to modelling technique (e.g. survival or logistic modelling), predictor selection, and risk group definition):*  Logistic modelling !? (a method like XGBoost is not modelling technique, I would say) | | | |
| *Describe whether and how the model was validated, either internally (e.g. bootstrapping, cross validation, random split sample) or externally (e.g. temporal validation, geographical validation, different setting, different type of participants):*  Cross validation was  used. | | | |
| *Describe the performance measures of the model, e.g. (re)calibration, discrimination, (re)classification, net benefit, and whether they were adjusted for optimism:*  The ROC analysis was performed. | | | |
| *Describe any participants who were excluded from the analysis:*  *To restrict our data to only primary COVID-19–related encounters, we excluded patients who had a first positive COVID-19 RT-PCR result more than two days after admission.* | | | |
| *Describe missing data on predictors and outcomes as well as methods used for missing data:* | | | |
|  | | Dev | Val |
| 1. Were there a reasonable number of participants with the outcome? | | N | N |
| 1. Were continuous and categorical predictors handled appropriately? | | PY | PY |
| 1. Were all enrolled participants included in the analysis? | | PY | PY |
| 1. Were participants with missing data handled appropriately? | | PY | PY |
| 1. Was selection of predictors based on univariable analysis avoided? | | PY | PY |
| 1. Were complexities in the data (e.g. censoring, competing risks, sampling of controls) accounted for appropriately? | | PY | PY |
| 1. Were relevant model performance measures evaluated appropriately? | | PY | PY |
| 1. Were model overfitting and optimism in model performance accounted for? | | PY | PY |
| 1. Do predictors and their assigned weights in the final model correspond to the results from multivariable analysis? | | PY | PY |
| **Risk of bias introduced by the analysis** | **RISK:**  *(low/ high/ unclear)* | **unclear** | **unclear** |
| *Rationale of bias rating:*  The analysis suffered from a  reasonable number of participants  with high imbalance of outcomes both for development and validation | | | |

**Step 4: Overall assessment**

| | **Reaching an overall judgement about risk of bias of the prediction model evaluation** | | | --- | --- | | **Low risk of bias** | If all domains were rated low risk of bias.  If a prediction model was developed without any external validation, and it was rated as low risk of bias for all domains, consider downgrading to **high risk of bias**. Such a model can only be considered as low risk of bias, if the development was based on a very large data set and included some form of internal validation. | | **High risk of bias** | If at least one domain is judged to be at **high risk of bias**. | | **Unclear risk of bias** | If an unclear risk of bias was noted in at least one domain and it was low risk for all other domains. |  | **Reaching an overall judgement about applicability of the prediction model evaluation** | | | --- | --- | | **Low concerns regarding applicability** | If low concerns regarding applicability for all domains, the prediction model evaluation is judged to have **low concerns regarding applicability**. | | **High concerns regarding applicability** | If high concerns regarding applicability for at least one domain, the prediction model evaluation is judged to have **high concerns regarding applicability**. | | **Unclear concerns regarding applicability** | If unclear concerns (but no “high concern”) regarding applicability for at least one domain, the prediction model evaluation is judged to have **unclear concerns regarding applicability** overall. | |
| --- | --- | --- | --- | --- | --- | --- | --- | --- | --- | --- | --- | --- | --- | --- | --- | --- |

| **Overall judgement about risk of bias and applicability of the prediction model evaluation** | | |
| --- | --- | --- |
| **Overall judgement of risk of bias** | **RISK:**  *(low/ high/ unclear)* | **high !?** |
| *Summary of sources of potential bias:*  Predictions solely on data extracted around patient admission (ie, within 36 hours).  There was no reference of whether the time interval between predictor assessment and outcome determined appropriate or not.  The analysis suffered from a  reasonable number of participants  with high imbalance of outcomes both for development and validation | | |
| **Overall judgement of applicability** | **CONCERN:**  *(low/ high/ unclear)* | **high** |
| *Summary of applicability concerns:*  *The study was conducted in a short period from March 15 to May 22, 2020 and included a small number of patients which is not enough for the prediction model development since additional patient data  with different periods could change the results.*  They used a subset of clinical variables from the EHR system and analysing other features could affect the model. Furthermore, the analysis was done during Covid-19 pandemic that could affected the results, for example criteria for SARS-CoV-2 testing.  Patients admitted to the hospital later in the crisis benefited from improved patient care protocols from experiential learning but were also negatively affected by resource constraints from overburdened hospitals. These effects may also induce temporal variation between patient outcomes, which is demonstrated by the lower critical event and mortality rate in the prospective validation data set.  The dataset that was used included a small number of patients which is not enough for the prediction model development since additional patient data could change the results. | | |

**Appendix 11**. Wanyan 2022 - Bias analysis using PROBAST.

**Step 1: Specify your systematic review question**

| **Criteria** | **Specify your systematic review question** |
| --- | --- |
| *Intended use of model:* | To propose a supervised pre-training model with a unique embedded k-nearest-neighbor positive sampling strategy for Electronic health records (EHR) data which is naturally heterogeneous and contains abundant sub-phenotype leading to high intra-class variance. c. The model used to  predict the mortality of COVID-19 patients. |
| ***Participants*** *including selection criteria and setting:* | Patients positive to COVID-19. |
| ***Predictors*** *(used in prediction modelling), including types of predictors (e.g. history, clinical examination, biochemical markers, imaging tests), time of measurement, specific measurement issues (e.g., any requirements/ prohibitions for specialized equipment):* | Demographics and clinical |
| *Outcome to be predicted:* | Death following COVID-19 diagnosis. |

**Step 2: Classify the type of prediction model evaluation**

| **Classify the evaluation based on its aim** | | | |
| --- | --- | --- | --- |
| **Type of prediction study** | **PROBAST boxes to complete** | **Tick as appropriate** | **Definition for type of prediction model study** |
| Development only | Development | ✔ | Prediction model development without external validation. These studies may include internal validation methods, such as bootstrapping and cross-validation techniques. |
| Development and validation | Development and validation | ✖ | Prediction model development combined with external validation in other participants in the same article. |
| Validation only | Validation | ✖ | External validation of existing (previously developed) model in other participants. |

|  | |
| --- | --- |
| **Publication reference** | Wanyan T, Lin M, Klang E, Menon KM, Gulamali FF, Azad A, Zhang Y, Ding Y, Wang Z, Wang F, Glicksberg B, Peng Y. Supervised Pretraining through Contrastive Categorical Positive Samplings to Improve COVID-19 Mortality Prediction. ACM BCB. 2022 Aug;2022:9. doi: 10.1145/3535508.3545541. Epub 2022 Aug 7. PMID: 35960866; PMCID: PMC9365529. | |
| **Models of interest** | Embedding-Based K Nearest Neighborhood (KNN) Positive Sampling Contrastive Learning (SEKPS-CL), KNN, Logistic Regression , Random Forest, Support Vector Machine (SVM), and Xgboost | |
| **Outcome of interest** | Mortality | |

**Step 3: Assess risk of bias and applicability**

| **DOMAIN 1:  Participants** | | | |
| --- | --- | --- | --- |
| **A. Risk of Bias** | | | |
| *Describe the sources of data and criteria for participant selection:*  DEV:  The EHR data of COVID-19 patients obtained from five hospitals within the Mount Sinai Health System located in New York City. The EHR data collected contains the following patient data: COVID-19 status, Intensive Care Unit (ICU) status, demographics, lab test results, vital signs, comorbid diseases, and outcome (e.g., mortality, discharge). Lab tests and vital signs were measured at multiple time points along the hospital course including nine frequently measured vital signs: heart rate, respiration rate, pulse oximetry, blood pressure (diastolic and systolic), temperature, oxygen saturation, height, and weight. other information were added during patients admission including age, gender, and race as demographics and 12 comorbid diseases: atrial fibrillation, asthma, coronary artery disease, cancer, chronic kidney disease, chronic obstructive pulmonary disease, diabetes mellitus, health failure, hypertension, stroke, alcoholism, and liver disease. | | | |
|  | | Dev | Val |
| 1. Were appropriate data sources used, e.g. cohort, RCT or nested case-control study data? | | Y |  |
| 1. Were all inclusions and exclusions of participants appropriate? | | PN |  |
| **Risk of bias introduced by selection of participants** | **RISK:**  *(low/ high/ unclear)* | **high** |  |
| *Rationale of bias rating:* | | | |
| There is a major concern related to the inclusion criteria. The time period when the study was designed is not clear. The study lacks to define the covariate assessment windows. for example how to capture disease  information of patients  in an appropriate period of time from their disease history. | | | |
| **B. Applicability** | | | |
| *Describe included participants, setting and dates:*  It has been mentioned in Domain 1 – Section A. | | | |
| **Concern that the included participants and setting do not match the review question** | **CONCERN:**  *(low/ high/ unclear)* | **low** |  |
| *Rationale of applicability rating:* | | | |
| In our review, our source population is the general population. Therefore, we did not restrict the source population to EHR data. Consequently, EHR data which covid-19 are part of the general population. | | | |

| **DOMAIN 2:  Predictors** | | | |
| --- | --- | --- | --- |
| **A. Risk of Bias** | | | |
| *List and describe predictors included in the final model, e.g. definition and timing of assessment:*  Age, gender, and race as demographics, COVID-19 status, Intensive Care Unit (ICU) status, lab test results: 76 lab tests, vital signs: s: heart rate, respiration rate, pulse oximetry, blood pressure (diastolic and systolic), temperature, oxygen saturation, height, and weight, comorbid diseases and 12 comorbid diseases: atrial fibrillation, asthma, coronary artery disease, cancer, chronic kidney disease, chronic obstructive pulmonary disease, diabetes mellitus, health failure, hypertension, stroke, alcoholism, and liver disease. . | | | |
|  | | Dev | Val |
| 1. Were predictors defined and assessed in a similar way for all participants? | | Y |  |
| 1. Were predictor assessments made without knowledge of outcome data? | | Y |  |
| 1. Are all predictors available at the time the model is intended to be used? | | Y |  |
| **Risk of bias introduced by predictors or their assessment** | **RISK:**  *(low/ high/ unclear)* | **low** |  |
| *Rationale of bias rating:* | | | |
| **B. Applicability** | | | |
| Concern that the definition, assessment or timing of predictors in the model do not match the review question | **CONCERN:**  *(low/ high/ unclear)* | **high** |  |
| *Rationale of applicability rating:*  Predictors were assessed prior to the occurrence of the outcome. Therefore, all predictors included in this article can be considered valid.  However, the timing of predictors in the mode is not clear. | | | |

| **DOMAIN 3: Outcome** | | | |
| --- | --- | --- | --- |
| **A. Risk of Bias** | | | |
| *Describe the outcome, how it was defined and determined, and the time interval between predictor assessment and outcome determination:*  Death in/out of hospital through follow-up of all patients in the study population was investigated.. | | | |
|  | | Dev | Val |
| 1. Was the outcome determined appropriately? | | PY |  |
| 1. Was a pre-specified or standard outcome definition used? | | PY |  |
| 1. Were predictors excluded from the outcome definition? | | Y |  |
| 1. Was the outcome defined and determined in a similar way for all participants? | | Y |  |
| 1. Was the outcome determined without knowledge of predictor information? | | PY |  |
| 1. Was the time interval between predictor assessment and outcome determination appropriate? | | NI |  |
| **Risk of bias introduced by the outcome or its determination** | **RISK:**  *(low/ high/ unclear)* | **high** |  |
| *Rationale of bias rating:*  We cannot excluded other aetiology for death after the patient left the hospital. There are no information regarding the time interval for the assessment of the predictors. | | | |
| **B. Applicability** | | | |
| *At what time point was the outcome determined:*  There is no information regarding the time interval for the assessment of the outcome.  *If a composite outcome was used, describe the relative frequency/distribution of each contributing outcome:* | | | |
| **Concern that the outcome, its definition, timing or determination do not match the review question** | **CONCERN:**  *(low/ high/ unclear)* | **high** |  |
| *Rationale of applicability rating: See above.* | | | |

| **DOMAIN 4: Analysis** | | | |
| --- | --- | --- | --- |
| **Risk of Bias** | | | |
| *Describe numbers of participants, number of candidate predictors, outcome events and events per candidate predictor:*  DEV: 7,067participants, 100 candidate predictors  all hospitalizations, ~23% mortality rate | | | |
| *Describe how the model was developed (for example in regards to modelling technique (e.g. survival or logistic modelling), predictor selection, and risk group definition):*  The method was divided into two groups. The first group of methods does not apply pre-training. The baseline models included: Logistic Regression, Random Forest,, Support Vector Machine (SVM), and XGboost. The input features for these baseline models are the averaged feature values (labs and vitals) in the observation window. Additionally, an LSTM model with Cross-Entropy Loss without pre-training was included. The second group contains two pre-training methodologies: Sim-CLR and SupCLR. SimCLR requires the augmented data generated from the training instance. Therefore, the positive sample by picking a temporal embedding from the longitudinal structure at a random time step was generated. For SupCLR, the same procedure was implemented from the original paper. | | | |
| *Describe whether and how the model was validated, either internally (e.g. bootstrapping, cross validation, random split sample) or externally (e.g. temporal validation, geographical validation, different setting, different type of participants):*  Cross validation has not been used. | | | |
| *Describe the performance measures of the model, e.g. (re)calibration, discrimination, (re)classification, net benefit, and whether they were adjusted for optimism:*  A ROC analysis was performed using a separate 2 × 2 “confusion matrix” for every possible threshold applied to the total score. The best threshold was defined by the highest value of the Youden Index. Point estimates and confidence intervals were computed using the bootstrap for sensitivity, specificity, Positive Predictive Value (PPV), Negative Predictive Value (NPV) and the De Long method for the Area Under the Curve (AUC)  Reliability analysis of the predictive formula was summarized using ROC curves showing optimal thresholds and sensitivity, specificity, AUC with point estimates and 95% confidence intervals. | | | |
| *Describe any participants who were excluded from the analysis:*  Not available. | | | |
| *Describe missing data on predictors and outcomes as well as methods used for missing data:*  Not available. | | | |
|  | | Dev | Val |
| 1. Were there a reasonable number of participants with the outcome? | | NI |  |
| 1. Were continuous and categorical predictors handled appropriately? | | PY |  |
| 1. Were all enrolled participants included in the analysis? | | PY |  |
| 1. Were participants with missing data handled appropriately? | | NI |  |
| 1. Was selection of predictors based on univariable analysis avoided? | | Y |  |
| 1. Were complexities in the data (e.g. censoring, competing risks, sampling of controls) accounted for appropriately? | | Y |  |
| 1. Were relevant model performance measures evaluated appropriately? | | Y |  |
| 1. Were model overfitting and optimism in model performance accounted for? | | N |  |
| 1. Do predictors and their assigned weights in the final model correspond to the results from multivariable analysis? | | NI |  |
| **Risk of bias introduced by the analysis** | **RISK:**  *(low/ high/ unclear)* | **high** |  |
| *Rationale of bias rating:*  *They use* averaged feature values for labs and vital data. Why not use the maximum value which can be more appropriate.  *They did not perform cross validation.*  They did not describe how they handled missing data. | | | |

**Step 4: Overall assessment**

| | **Reaching an overall judgement about risk of bias of the prediction model evaluation** | | | --- | --- | | **Low risk of bias** | If all domains were rated low risk of bias.  If a prediction model was developed without any external validation, and it was rated as low risk of bias for all domains, consider downgrading to **high risk of bias**. Such a model can only be considered as low risk of bias, if the development was based on a very large data set and included some form of internal validation. | | **High risk of bias** | If at least one domain is judged to be at **high risk of bias**. | | **Unclear risk of bias** | If an unclear risk of bias was noted in at least one domain and it was low risk for all other domains. |  | **Reaching an overall judgement about applicability of the prediction model evaluation** | | | --- | --- | | **Low concerns regarding applicability** | If low concerns regarding applicability for all domains, the prediction model evaluation is judged to have **low concerns regarding applicability**. | | **High concerns regarding applicability** | If high concerns regarding applicability for at least one domain, the prediction model evaluation is judged to have **high concerns regarding applicability**. | | **Unclear concerns regarding applicability** | If unclear concerns (but no “high concern”) regarding applicability for at least one domain, the prediction model evaluation is judged to have **unclear concerns regarding applicability** overall. | |
| --- | --- | --- | --- | --- | --- | --- | --- | --- | --- | --- | --- | --- | --- | --- | --- | --- |

| **Overall judgement about risk of bias and applicability of the prediction model evaluation** | | |
| --- | --- | --- |
| **Overall judgement of risk of bias** | **RISK:**  *(low/ high/ unclear)* | **high** |
| *Summary of sources of potential bias:*  *They use* averaged feature values for labs and vital data. Why not use the maximum value which can be more appropriate.  *They did not perform cross validation.*  They did not describe how they handled missing data. | | |
| **Overall judgement of applicability** | **CONCERN:**  *(low/ high/ unclear)* | **high** |
| *Summary of applicability concerns:*  There is no information regarding the time interval for the assessment of the outcome. | | |

**Appendix 12**. Vezzoli 2022 - Bias analysis using PROBAST.

**Step 1: Specify your systematic review question**

| **Criteria** | **Specify your systematic review question** |
| --- | --- |
| *Intended use of model:* | It aims to obtain a risk score for in-hospital mortality in patients with coronavirus disease infection (COVID-19) based on a limited number of features collected at hospital admission. |
| ***Participants*** *including selection criteria and setting:* | infected COVID-19 patients, who were hospitalized in cardiology units |
| ***Predictors*** *(used in prediction modelling), including types of predictors (e.g. history, clinical examination, biochemical markers, imaging tests), time of measurement, specific measurement issues (e.g., any requirements/ prohibitions for specialized equipment):* | demographics, medical history (particularly cardiovascular diseases), and in-hospital clinical course including treatments |
| *Outcome to be predicted:* | Mortality |

**Step 2: Classify the type of prediction model evaluation**

| **Classify the evaluation based on its aim** | | | |
| --- | --- | --- | --- |
| **Type of prediction study** | **PROBAST boxes to complete** | **Tick as appropriate** | **Definition for type of prediction model study** |
| Development only | Development | ✔ | Prediction model development without external validation. These studies may include internal validation methods, such as bootstrapping and cross-validation techniques. |
| Development and validation | Development and validation | ✖ | Prediction model development combined with external validation in other participants in the same article. |
| Validation only | Validation | ✖ | External validation of existing (previously developed) model in other participants. |

|  | |
| --- | --- |
| **Publication reference** | Vezzoli M, Inciardi RM, Oriecuia C, Paris S, Murillo NH, Agostoni P, Ameri P, Bellasi A, Camporotondo R, Canale C, Carubelli V, Carugo S, Catagnano F, Danzi G, Dalla Vecchia L, Giovinazzo S, Gnecchi M, Guazzi M, Iorio A, La Rovere MT, Leonardi S, Maccagni G, Mapelli M, Margonato D, Merlo M, Monzo L, Mortara A, Nuzzi V, Pagnesi M, Piepoli M, Porto I, Pozzi A, Provenzale G, Sarullo F, Senni M, Sinagra G, Tomasoni D, Adamo M, Volterrani M, Maroldi R, Metra M, Lombardi CM, Specchia C. Machine learning for prediction of in-hospital mortality in coronavirus disease 2019 patients: results from an Italian multicenter study. J Cardiovasc Med (Hagerstown). 2022 Jul 1;23(7):439-446. doi: 10.2459/JCM.0000000000001329. PMID: 35763764. | |
| **Models of interest** | Random Forest, Gradient Boosting Machine (GBM), logistic regression |  |
| **Outcome of interest** | Mortality | |

**Step 3: Assess risk of bias and applicability**

| **DOMAIN 1:  Participants** | | | |
| --- | --- | --- | --- |
| **A. Risk of Bias** | | | |
| *Describe the sources of data and criteria for participant selection:*  DEV:  This multicenter observational study involved a cohort of consecutive adult Caucasian patients with laboratory-confirmed COVID-19 who were hospitalized in 13 Italian cardiology units from 1 March to 9 April 2020. Acute cardiovascular diagnosis (i.e. acute heart failure, acute coronary syndrome and new-onset arrhythmias) upon admission were exclusion criteria. | | | |
|  | | Dev | Val |
| 1. Were appropriate data sources used, e.g. cohort, RCT or nested case-control study data? | | Y |  |
| 1. Were all inclusions and exclusions of participants appropriate? | | PY |  |
| **Risk of bias introduced by selection of participants** | **RISK:**  *(low/ high/ unclear)* | **low** |  |
| *Rationale of bias rating: No Concerns* | | | |
|  | | | |
| **B. Applicability** | | | |
| *Describe included participants, setting and dates:*  It has been mentioned in Domain 1 – Section A. | | | |
| **Concern that the included participants and setting do not match the review question** | **CONCERN:**  *(low/ high/ unclear)* | **low** |  |
| *Rationale of applicability rating:* | | | |
| In our review, our source population is the general population. Therefore, we did not restrict the source population to the data obtained from cardiology units. Consequently, their data are part of the general population. | | | |

| **DOMAIN 2:  Predictors** | | | |
| --- | --- | --- | --- |
| **A. Risk of Bias** | | | |
| *List and describe predictors included in the final model, e.g. definition and timing of assessment:*  Patients’ data at admission were extracted from the electronic medical records of each designated hospital. Detailed demographics information, medical history (particularly cardiovascular diseases), and in-hospital clinical course including treatments were recorded. Laboratory examinations including routine blood tests; lymphocyte subsets; inflammatory or infection-related biomarkers; and cardiac, renal, liver, and coagulation function tests were obtained at initial diagnosis. Renal function was measured as estimated glomerular filtration rate (eGFR) and was calculated by the chronic kidney disease epidemiology collaboration equation; chronic kidney disease was defined when eGFR was less than 60 ml/kg/1.73 m^2^. Cardiac injury was defined by plasma levels of high-sensitivity troponin, either troponin T or troponin I, greater than the 99th percentile of normal values, as per manufacturer's indications. | | | |
|  | | Dev | Val |
| 1. Were predictors defined and assessed in a similar way for all participants? | | PY |  |
| 1. Were predictor assessments made without knowledge of outcome data? | | PY |  |
| 1. Are all predictors available at the time the model is intended to be used? | | Y |  |
| **Risk of bias introduced by predictors or their assessment** | **RISK:**  *(low/ high/ unclear)* | **low** |  |
| *Rationale of bias rating:*  *No Concerns* | | | |
| **B. Applicability** | | | |
| Concern that the definition, assessment or timing of predictors in the model do not match the review question | **CONCERN:**  *(low/ high/ unclear)* | **High** |  |
| *Rationale of applicability rating:*  The higher proportion of cardiovascular comorbidities in the population may have influenced the observed results. | | | |

| **DOMAIN 3: Outcome** | | | |
| --- | --- | --- | --- |
| **A. Risk of Bias** | | | |
| *Describe the outcome, how it was defined and determined, and the time interval between predictor assessment and outcome determination:*  Patients were followed up after the COVID-19 diagnosis and all causes of in-hospital mortality or discharge were ascertained until 23 April 2020 | | | |
|  | | Dev | Val |
| 1. Was the outcome determined appropriately? | | PY |  |
| 1. Was a pre-specified or standard outcome definition used? | | PY |  |
| 1. Were predictors excluded from the outcome definition? | | Y |  |
| 1. Was the outcome defined and determined in a similar way for all participants? | | PY |  |
| 1. Was the outcome determined without knowledge of predictor information? | | PY |  |
| 1. Was the time interval between predictor assessment and outcome determination appropriate? | | N |  |
| **Risk of bias introduced by the outcome or its determination** | **RISK:**  *(low/ high/ unclear)* | **High** |  |
| *Rationale of bias rating:*  There is a concern regarding the time interval between predictors and outcome. | | | |
| **B. Applicability** | | | |
| *At what time point was the outcome determined:*  The analysis lacks postdischarge follow-up data, thus it could not assess long-term mortality.  *If a composite outcome was used, describe the relative frequency/distribution of each contributing outcome:* | | | |
| **Concern that the outcome, its definition, timing or determination do not match the review question** | **CONCERN:**  *(low/ high/ unclear)* | **high** |  |
| *Rationale of applicability rating:*  *The study has been done from the first pandemic wave when no effective treatment strategies were available.* The outcome was characterized by a significant burden of cardiovascular comorbidities justifying the relatively high rate of death. | | | |
| **DOMAIN 4: Analysis** | | | |
| **Risk of Bias** | | | |
| *Describe numbers of participants, number of candidate predictors, outcome events and events per candidate predictor:*  DEV: 701 patients enrolled , 165 (23.5%) died during a median hospitalization | | | |
| *Describe how the model was developed (for example in regards to modelling technique (e.g. survival or logistic modelling), predictor selection, and risk group definition):*  Random Forest, Gradient Boosting Machine (GBM), logistic regression | | | |
| *Describe whether and how the model was validated, either internally (e.g. bootstrapping, cross validation, random split sample) or externally (e.g. temporal validation, geographical validation, different setting, different type of participants):*  Cross validation has been used. | | | |
| *Describe the performance measures of the model, e.g. (re)calibration, discrimination, (re)classification, net benefit, and whether they were adjusted for optimism:*  Model accuracy was reported. The ROC analysis was performed. | | | |
| *Describe any participants who were excluded from the analysis:*  Not available. | | | |
| *Describe missing data on predictors and outcomes as well as methods used for missing data:*  Not available. | | | |
|  | | Dev | Val |
| 1. Were there a reasonable number of participants with the outcome? | | Y |  |
| 1. Were continuous and categorical predictors handled appropriately? | | PY |  |
| 1. Were all enrolled participants included in the analysis? | | PY |  |
| 1. Were participants with missing data handled appropriately? | | PY |  |
| 1. Was selection of predictors based on univariable analysis avoided? | | PY |  |
| 1. Were complexities in the data (e.g. censoring, competing risks, sampling of controls) accounted for appropriately? | | PY |  |
| 1. Were relevant model performance measures evaluated appropriately? | | Y |  |
| 1. Were model overfitting and optimism in model performance accounted for? | | PY |  |
| 1. Do predictors and their assigned weights in the final model correspond to the results from multivariable analysis? | | PY |  |
| **Risk of bias introduced by the analysis** | **RISK:**  *(low/ high/ unclear)* | **LOW** |  |
| *Rationale of bias rating:* | | | |

**Step 4: Overall assessment**

| | **Reaching an overall judgement about risk of bias of the prediction model evaluation** | | | --- | --- | | **Low risk of bias** | If all domains were rated low risk of bias.  If a prediction model was developed without any external validation, and it was rated as low risk of bias for all domains, consider downgrading to **high risk of bias**. Such a model can only be considered as low risk of bias, if the development was based on a very large data set and included some form of internal validation. | | **High risk of bias** | If at least one domain is judged to be at **high risk of bias**. | | **Unclear risk of bias** | If an unclear risk of bias was noted in at least one domain and it was low risk for all other domains. |  | **Reaching an overall judgement about applicability of the prediction model evaluation** | | | --- | --- | | **Low concerns regarding applicability** | If low concerns regarding applicability for all domains, the prediction model evaluation is judged to have **low concerns regarding applicability**. | | **High concerns regarding applicability** | If high concerns regarding applicability for at least one domain, the prediction model evaluation is judged to have **high concerns regarding applicability**. | | **Unclear concerns regarding applicability** | If unclear concerns (but no “high concern”) regarding applicability for at least one domain, the prediction model evaluation is judged to have **unclear concerns regarding applicability** overall. | |
| --- | --- | --- | --- | --- | --- | --- | --- | --- | --- | --- | --- | --- | --- | --- | --- | --- |

| **Overall judgement about risk of bias and applicability of the prediction model evaluation** | | |
| --- | --- | --- |
| **Overall judgement of risk of bias** | **RISK:**  *(low/ high/ unclear)* | **high** |
| *Summary of sources of potential bias:*  *The study has been done from the first pandemic wave when no effective treatment strategies were available.* The outcome was characterized by a significant burden of cardiovascular comorbidities justifying the relatively high rate of death. The analysis lacks postdischarge follow-up data, thus it could not assess long-term mortality. | | |
| **Overall judgement of applicability** | **CONCERN:**  *(low/ high/ unclear)* | **high** |
| *Summary of applicability concerns:* | | |

**Appendix 13**. Ali 2022 - Bias analysis using PROBAST.

**Step 1: Specify your systematic review question**

| **Criteria** | **Specify your systematic review question** |
| --- | --- |
| *Intended use of model:* | It aims to predict patient mortality and likelihood of testing positive/negative for COVID-19 as a function of many different factors. |
| ***Participants*** *including selection criteria and setting:* | Infected COVID-19 patients |
| ***Predictors*** *(used in prediction modelling), including types of predictors (e.g. history, clinical examination, biochemical markers, imaging tests), time of measurement, specific measurement issues (e.g., any requirements/ prohibitions for specialized equipment):* | Clinical, demographics,  laboratory |
| *Outcome to be predicted:* | Mortality |

**Step 2: Classify the type of prediction model evaluation**

| **Classify the evaluation based on its aim** | | | |
| --- | --- | --- | --- |
| **Type of prediction study** | **PROBAST boxes to complete** | **Tick as appropriate** | **Definition for type of prediction model study** |
| Development only | Development | ✔ | Prediction model development without external validation. These studies may include internal validation methods, such as bootstrapping and cross-validation techniques. |
| Development and validation | Development and validation | ✖ | Prediction model development combined with external validation in other participants in the same article. |
| Validation only | Validation | ✖ | External validation of existing (previously developed) model in other participants. |

|  | |
| --- | --- |
| **Publication reference** | Ali S, Zhou Y, Patterson M. Efficient analysis of COVID-19 clinical data using machine learning models. Med Biol Eng Comput. 2022 Jul;60(7):1881-1896. doi: 10.1007/s11517-022-02570-8. Epub 2022 May 4. PMID: 35507111; PMCID: PMC9066140. | |
| **Models of interest** | Support Vector Machine (SVM), Naive Bayes (NB), Multiple Linear Regression (MLP), K-Nearest Neighbors (KNN), Random Forest (RF), Logistic Regression (LR), and Decision Tree (DT) |  |
| **Outcome of interest** | Mortality | |

**Step 3: Assess risk of bias and applicability**

| **DOMAIN 1:  Participants** | | | |
| --- | --- | --- | --- |
| **A. Risk of Bias** | | | |
| *Describe the sources of data and criteria for participant selection:*  DEV:  Two data sets were used: 1-COVID-19 Case Surveillance dataset, which is publicly available on the Centers for Disease Control and Prevention CDC, USA’s website included factors such as age group, sex, ethnicity, and residence, 2-patients with COVID-19 in the Israelita Albert Einstein Hospital in Sao Paulo, Brazil which included many factors from a blood test, such as leukocytes, platelets, and red blood cells counts.. | | | |
|  | | Dev | Val |
| 1. Were appropriate data sources used, e.g. cohort, RCT or nested case-control study data? | | PN |  |
| 1. Were all inclusions and exclusions of participants appropriate? | | PY |  |
| **Risk of bias introduced by selection of participants** | **RISK:**  *(low/ high/ unclear)* | **High** |  |
| *Rationale of bias rating:* | | | |
| It i not clear how two data sets were used or combine together. | | | |
| **B. Applicability** | | | |
| *Describe included participants, setting and dates:*  It has been mentioned in Domain 1 – Section A. | | | |
| **Concern that the included participants and setting do not match the review question** | **CONCERN:**  *(low/ high/ unclear)* | **low** |  |
| *Rationale of applicability rating:* | | | |
| In our review, our source population is the general population. Therefore, we did not restrict the source population to the data obtained from blood test. Consequently, their data are part of the general population. | | | |

| **DOMAIN 2:  Predictors** | | | |
| --- | --- | --- | --- |
| **A. Risk of Bias** | | | |
| *List and describe predictors included in the final model, e.g. definition and timing of assessment:*  age group, sex, ethnicity, residence, exposure, conditions include diabetes mellitus, hypertension, severe obesity (occurs when BMI is greater than 40), cardiovascular disease, chronic renal disease, chronic liver disease, chronic lung disease, other chronic diseases, immunosuppressive condition, autoimmune condition, current smoker, former smoker, substance abuse or misuse, disability, psychological/psychiatric, pregnancy, other. Red blood Cells, Hemoglobin, Platelets, Hematocrit, Aspartate transaminase, Lymphocytes, Monocytes, Sodium, Urea, Basophils, Creatinine, Serum Glucose, Alanine transaminase, Leukocytes, Potassium, Eosinophils, Proteina C reativa mg/dL, Neutrophils, SARS-Cov-2 exam result (positive or negative) | | | |
|  | | Dev | Val |
| 1. Were predictors defined and assessed in a similar way for all participants? | | PN |  |
| 1. Were predictor assessments made without knowledge of outcome data? | | PY |  |
| 1. Are all predictors available at the time the model is intended to be used? | | PN |  |
| **Risk of bias introduced by predictors or their assessment** | **RISK:**  *(low/ high/ unclear)* | **High** |  |
| *Rationale of bias rating:*  They used two different data sources which contains different variables and it is not clear how the two data sets were matched at the time the model was used. | | | |
| **B. Applicability** | | | |
| Concern that the definition, assessment or timing of predictors in the model do not match the review question | **CONCERN:**  *(low/ high/ unclear)* | **High** |  |
| *Rationale of applicability rating:*  The time time interval in the two data sets are different | | | |

| **DOMAIN 3: Outcome** | | | |
| --- | --- | --- | --- |
| **A. Risk of Bias** | | | |
| *Describe the outcome, how it was defined and determined, and the time interval between predictor assessment and outcome determination:*  Data set 2 included Patients with COVID-19 at the beginning of the year 2020 | | | |
|  | | Dev | Val |
| 1. Was the outcome determined appropriately? | | PY |  |
| 1. Was a pre-specified or standard outcome definition used? | | PY |  |
| 1. Were predictors excluded from the outcome definition? | | Y |  |
| 1. Was the outcome defined and determined in a similar way for all participants? | | PY |  |
| 1. Was the outcome determined without knowledge of predictor information? | | PY |  |
| 1. Was the time interval between predictor assessment and outcome determination appropriate? | | N |  |
| **Risk of bias introduced by the outcome or its determination** | **RISK:**  *(low/ high/ unclear)* | **High** |  |
| *Rationale of bias rating:* There is a concerns regarding the time interval between predictors and outcome. | | | |
| **B. Applicability** | | | |
| *At what time point was the outcome determined:*  The analysis lacks postdischarge follow-up data, thus it could not assess long-term mortality.  *If a composite outcome was used, describe the relative frequency/distribution of each contributing outcome:* | | | |
| **Concern that the outcome, its definition, timing or determination do not match the review question** | **CONCERN:**  *(low/ high/ unclear)* | **high** |  |
| *Rationale of applicability rating:*  *The study has been done from the first pandemic wave when no effective treatment strategies were available.* | | | |
| **DOMAIN 4: Analysis** | | | |
| **Risk of Bias** | | | |
| *Describe numbers of participants, number of candidate predictors, outcome events and events per candidate predictor:*  DEV:  data set1: 95984 patients with 19 features.  data set2: 608 patients enrolled, 84 had Covid_19 with 18 laboratory findings. | | | |
| *Describe how the model was developed (for example in regards to modelling technique (e.g. survival or logistic modelling), predictor selection, and risk group definition):*  Support Vector Machine (SVM), Naive Bayes (NB), Multiple Linear Regression (MLP), K-Nearest Neighbors (KNN), Random Forest (RF), Logistic Regression (LR), and Decision Tree (DT) | | | |
| *Describe whether and how the model was validated, either internally (e.g. bootstrapping, cross validation, random split sample) or externally (e.g. temporal validation, geographical validation, different setting, different type of participants):*  Cross validation has been used. | | | |
| *Describe the performance measures of the model, e.g. (re)calibration, discrimination, (re)classification, net benefit, and whether they were adjusted for optimism:*  Model accuracy was reported. The ROC analysis was performed. | | | |
| *Describe any participants who were excluded from the analysis:*  Not available. | | | |
| *Describe missing data on predictors and outcomes as well as methods used for missing data:*  Not available. | | | |
|  | | Dev | Val |
| 1. Were there a reasonable number of participants with the outcome? | | Y |  |
| 1. Were continuous and categorical predictors handled appropriately? | | PY |  |
| 1. Were all enrolled participants included in the analysis? | | PY |  |
| 1. Were participants with missing data handled appropriately? | | PY |  |
| 1. Was selection of predictors based on univariable analysis avoided? | | PY |  |
| 1. Were complexities in the data (e.g. censoring, competing risks, sampling of controls) accounted for appropriately? | | PY |  |
| 1. Were relevant model performance measures evaluated appropriately? | | Y |  |
| 1. Were model overfitting and optimism in model performance accounted for? | | PY |  |
| 1. Do predictors and their assigned weights in the final model correspond to the results from multivariable analysis? | | PY |  |
| **Risk of bias introduced by the analysis** | **RISK:**  *(low/ high/ unclear)* | **LOW** |  |
| *Rationale of bias rating:* | | | |

**Step 4: Overall assessment**

| | **Reaching an overall judgement about risk of bias of the prediction model evaluation** | | | --- | --- | | **Low risk of bias** | If all domains were rated low risk of bias.  If a prediction model was developed without any external validation, and it was rated as low risk of bias for all domains, consider downgrading to **high risk of bias**. Such a model can only be considered as low risk of bias, if the development was based on a very large data set and included some form of internal validation. | | **High risk of bias** | If at least one domain is judged to be at **high risk of bias**. | | **Unclear risk of bias** | If an unclear risk of bias was noted in at least one domain and it was low risk for all other domains. |  | **Reaching an overall judgement about applicability of the prediction model evaluation** | | | --- | --- | | **Low concerns regarding applicability** | If low concerns regarding applicability for all domains, the prediction model evaluation is judged to have **low concerns regarding applicability**. | | **High concerns regarding applicability** | If high concerns regarding applicability for at least one domain, the prediction model evaluation is judged to have **high concerns regarding applicability**. | | **Unclear concerns regarding applicability** | If unclear concerns (but no “high concern”) regarding applicability for at least one domain, the prediction model evaluation is judged to have **unclear concerns regarding applicability** overall. | |
| --- | --- | --- | --- | --- | --- | --- | --- | --- | --- | --- | --- | --- | --- | --- | --- | --- |

| **Overall judgement about risk of bias and applicability of the prediction model evaluation** | | |
| --- | --- | --- |
| **Overall judgement of risk of bias** | **RISK:**  *(low/ high/ unclear)* | **high** |
| *Summary of sources of potential bias:*  *The study has been done from the first pandemic wave when no effective treatment strategies were available.* The analysis lacks postdischarge follow-up data, thus it could not assess long-term mortality. It is not clear how the two datasets could be used together and matched. | | |
| **Overall judgement of applicability** | **CONCERN:**  *(low/ high/ unclear)* | **high** |
| *Summary of applicability concerns:* | | |

**Appendix 14**. Zarei 2022 - Bias analysis using PROBAST.

**Step 1: Specify your systematic review question**

| **Criteria** | **Specify your systematic review question** |
| --- | --- |
| *Intended use of model:* | To develop machine learning models to predict in-hospital death among COVID-19patients |
| ***Participants*** *including selection criteria and setting:* | Patients positive to COVID-19. |
| ***Predictors*** *(used in prediction modelling), including types of predictors (e.g. history, clinical examination, biochemical markers, imaging tests), time of measurement, specific measurement issues (e.g., any requirements/ prohibitions for specialized equipment):* | Demographics, clinical |
| *Outcome to be predicted:* | Death following COVID-19 diagnosis. |

**Step 2: Classify the type of prediction model evaluation**

| **Classify the evaluation based on its aim** | | | |
| --- | --- | --- | --- |
| **Type of prediction study** | **PROBAST boxes to complete** | **Tick as appropriate** | **Definition for type of prediction model study** |
| Development only | Development | ✖ | Prediction model development without external validation. These studies may include internal validation methods, such as bootstrapping and cross-validation techniques. |
| Development and validation | Development and validation | ✔ | Prediction model development combined with external validation in other participants in the same article. |
| Validation only | Validation | ✖ | External validation of existing (previously developed) model in other participants. |

|  | |
| --- | --- |
| **Publication reference** | Zarei J, Jamshidnezhad A, Haddadzadeh Shoushtari M, Mohammad Hadianfard A, Cheraghi M, Sheikhtaheri A. Machine Learning Models to Predict In-Hospital Mortality among Inpatients with COVID-19: Underestimation and Overestimation Bias Analysis in Subgroup Populations. J Healthc Eng. 2022 Jun 23;2022:1644910. doi: 10.1155/2022/1644910. PMID: 35756093; PMCID: PMC9226971. | |
| **Models of interest** | Chi-Squared Detection of Automatic Interaction (CHAID),  C5, and Random Forest (RF) decision trees, SVM,  Multiple Layer Perceptron (MLP) neural networks and Bayesian network | |
| **Outcome of interest** | Mortality | |

**Step 3: Assess risk of bias and applicability**

| **DOMAIN 1:  Participants** | | | |
| --- | --- | --- | --- |
| **A. Risk of Bias** | | | |
| *Describe the sources of data and criteria for participant selection:*  DEV: data was from the Khuzestan-Iran COVID-19 registry system belonging to Ahvaz Jundishapure University of Medical Sciences (AJUMS). This registry collects demographic data, signs and symptoms, patient outcomes, PCR and CT results, and comorbidities from 38 hospitals. Furthermore, only patients who were hospitalized for more than 24 hours were included.  VAL: external validation data were from four different hospitals in different time frames from the Khuzestan COVID-19 registry system. | | | |
|  | | Dev | Val |
| 1. Were appropriate data sources used, e.g. cohort, RCT or nested case-control study data? | | Y | PY |
| 1. Were all inclusions and exclusions of participants appropriate? | | PN | PN |
| **Risk of bias introduced by selection of participants** | **RISK:**  *(low/ high/ unclear)* | **high** | **high** |
| *Rationale of bias rating:* | | | |
| In the discussion section it is mentioned that only the subpopulation bias based on gender and age group was analysed. It could be potentially bias | | | |
| **B. Applicability** | | | |
| *Describe included participants, setting and dates:*  It has been mentioned in Domain 1 – Section A. | | | |
| **Concern that the included participants and setting do not match the review question** | **CONCERN:**  *(low/ high/ unclear)* | **low** | **low** |
| *Rationale of applicability rating:* | | | |
| In our review, our source population is the general population which is in accordance with the data used in this study. | | | |

| **DOMAIN 2:  Predictors** | | | |
| --- | --- | --- | --- |
| **A. Risk of Bias** | | | |
| *List and describe predictors included in the final model, e.g. definition and timing of assessment:*  Intubation, number of comorbidities, age, gender, respiratory distress, blood oxygen saturation level, ICU admission, cough, unconsciousness, positive PCR, and abnormal CT | | | |
|  | | Dev | Val |
| 1. Were predictors defined and assessed in a similar way for all participants? | | Y | Y |
| 1. Were predictor assessments made without knowledge of outcome data? | | Y | Y |
| 1. Are all predictors available at the time the model is intended to be used? | | Y | Y |
| **Risk of bias introduced by predictors or their assessment** | **RISK:**  *(low/ high/ unclear)* | **low** | **low** |
| *Rationale of bias rating:* | | | |
| **B. Applicability** | | | |
| Concern that the definition, assessment or timing of predictors in the model do not match the review question | **CONCERN:**  *(low/ high/ unclear)* | **low** | **low** |
| *Rationale of applicability rating:*  Predictors were assessed prior to the occurrence of the outcome. Therefore, all predictors included in this article can be considered valid. | | | |

| **DOMAIN 3: Outcome** | | | |
| --- | --- | --- | --- |
| **A. Risk of Bias** | | | |
| *Describe the outcome, how it was defined and determined, and the time interval between predictor assessment and outcome determination:*  They investigated death through follow-up of all patients in the study population. | | | |
|  | | Dev | Val |
| 1. Was the outcome determined appropriately? | | PY | PY |
| 1. Was a pre-specified or standard outcome definition used? | | PY | PY |
| 1. Were predictors excluded from the outcome definition? | | Y | Y |
| 1. Was the outcome defined and determined in a similar way for all participants? | | Y | Y |
| 1. Was the outcome determined without knowledge of predictor information? | | PY | PY |
| 1. Was the time interval between predictor assessment and outcome determination appropriate? | | NI | NI |
| **Risk of bias introduced by the outcome or its determination** | **RISK:**  *(low/ high/ unclear)* | **high** | **high** |
| *Rationale of bias rating:*  There is no information regarding the time interval for the assessment of the predictors. | | | |
| **B. Applicability** | | | |
| *At what time point was the outcome determined:*  There is no information regarding the time interval for the assessment of the outcome.  *If a composite outcome was used, describe the relative frequency/distribution of each contributing outcome:* | | | |
| **Concern that the outcome, its definition, timing or determination do not match the review question** | **CONCERN:**  *(low/ high/ unclear)* | **high** | **high** |
| *Rationale of applicability rating: see above.* | | | |

| **DOMAIN 4: Analysis** | | | |
| --- | --- | --- | --- |
| **Risk of Bias** | | | |
| *Describe numbers of participants, number of candidate predictors, outcome events and events per candidate predictor:*  DEV: 10,657 participants, 1711 (16.06%) deaths 60 candidate predictors  VAL: 1425 participants, 309 deaths and 60 candidate predictors | | | |
| *Describe how the model was developed (for example in regards to modelling technique (e.g. survival or logistic modelling), predictor selection, and risk group definition):*  Support Vector Machine (SVM), Naive Bayes (NB), Multiple Linear Regression (MLP), K-Nearest Neighbors (KNN), Random Forest (RF), Logistic Regression (LR), and Decision Tree (DT) | | | |
| *Describe whether and how the model was validated, either internally (e.g. bootstrapping, cross validation, random split sample) or externally (e.g. temporal validation, geographical validation, different setting, different type of participants):*  Not available. | | | |
| *Describe the performance measures of the model, e.g. (re)calibration, discrimination, (re)classification, net benefit, and whether they were adjusted for optimism:*  A ROC analysis was performed using a separate 2 × 2 “confusion matrix” for every possible threshold applied to the total score. The best threshold was defined by the highest value of the Youden Index. Point estimates and confidence intervals were computed using the bootstrap for sensitivity, specificity, Positive Predictive Value (PPV), Negative Predictive Value (NPV) and the De Long method for the Area Under the Curve (AUC)  Reliability analysis of the predictive formula was summarized using ROC curves showing optimal thresholds and sensitivity, specificity, AUC with point estimates and 95% confidence intervals. | | | |
| *Describe any participants who were excluded from the analysis:*  Outpatients and hospitalized patients with a short stay (less than 24 hours) were excluded from the final analysis. | | | |
| *Describe missing data on predictors and outcomes as well as methods used for missing data:*  The database had a low rate of missing data. The 28 variables had a missing rate below 4%. The missing values with the mean for age and the highest frequency of values for nonnumerical variables were imputed. | | | |
|  | | Dev | Val |
| 1. Were there a reasonable number of participants with the outcome? | | NI | NI |
| 1. Were continuous and categorical predictors handled appropriately? | | PN | PN |
| 1. Were all enrolled participants included in the analysis? | | PY | PY |
| 1. Were participants with missing data handled appropriately? | | NI | NI |
| 1. Was selection of predictors based on univariable analysis avoided? | | Y | Y |
| 1. Were complexities in the data (e.g. censoring, competing risks, sampling of controls) accounted for appropriately? | | Y | Y |
| 1. Were relevant model performance measures evaluated appropriately? | | Y | Y |
| 1. Were model overfitting and optimism in model performance accounted for? | | Y | Y |
| 1. Do predictors and their assigned weights in the final model correspond to the results from multivariable analysis? | | NI | NI |
| **Risk of bias introduced by the analysis** | **RISK:**  *(low/ high/ unclear)* | **high** | **high** |
| *Rationale of bias rating:*  There is great concern regarding how the missing values for age and nonnumerical variables were imputed. Moreover all variables in the data set except age are binary which can cause bias in the results as age variable can have more effect in some machine learning models. | | | |

**Step 4: Overall assessment**

| | **Reaching an overall judgement about risk of bias of the prediction model evaluation** | | | --- | --- | | **Low risk of bias** | If all domains were rated low risk of bias.  If a prediction model was developed without any external validation, and it was rated as low risk of bias for all domains, consider downgrading to **high risk of bias**. Such a model can only be considered as low risk of bias, if the development was based on a very large data set and included some form of internal validation. | | **High risk of bias** | If at least one domain is judged to be at **high risk of bias**. | | **Unclear risk of bias** | If an unclear risk of bias was noted in at least one domain and it was low risk for all other domains. |  | **Reaching an overall judgement about applicability of the prediction model evaluation** | | | --- | --- | | **Low concerns regarding applicability** | If low concerns regarding applicability for all domains, the prediction model evaluation is judged to have **low concerns regarding applicability**. | | **High concerns regarding applicability** | If high concerns regarding applicability for at least one domain, the prediction model evaluation is judged to have **high concerns regarding applicability**. | | **Unclear concerns regarding applicability** | If unclear concerns (but no “high concern”) regarding applicability for at least one domain, the prediction model evaluation is judged to have **unclear concerns regarding applicability** overall. | |
| --- | --- | --- | --- | --- | --- | --- | --- | --- | --- | --- | --- | --- | --- | --- | --- | --- |

| **Overall judgement about risk of bias and applicability of the prediction model evaluation** | | |
| --- | --- | --- |
| **Overall judgement of risk of bias** | **RISK:**  *(low/ high/ unclear)* | **high** |
| *Summary of sources of potential bias:*  Outpatients and hospitalized patients with a short stay (less than 24 hours) were excluded from the final analysis.  There is great concern regarding how the missing values for age and nonnumerical variables were imputed. Moreover all variables in the data set except age are binary which can cause bias in the results as age variable can have more effect in some machine learning models. | | |
| **Overall judgement of applicability** | **CONCERN:**  *(low/ high/ unclear)* | **high** |
| *Summary of applicability concerns:* | | |

**Appendix 15**. Wang 2020 - Bias analysis using PROBAST.

**Step 1: Specify your systematic review question**

| **Criteria** | **Specify your systematic review question** |
| --- | --- |
| *Intended use of model:* | To  develop accurate models to predict the mortality of hospitalized patients with COVID-19 |
| ***Participants*** *including selection criteria and setting:* | Positive tested COVID-19 patients and hospitalized |
| ***Predictors*** *(used in prediction modelling), including types of predictors (e.g. history, clinical examination, biochemical markers, imaging tests), time of measurement, specific measurement issues (e.g., any requirements/ prohibitions for specialized equipment):* | Epidemiological, demographic, clinical, and laboratory |
| *Outcome to be predicted:* | Mortality |

**Step 2: Classify the type of prediction model evaluation**

| **Classify the evaluation based on its aim** | | | |
| --- | --- | --- | --- |
| **Type of prediction study** | **PROBAST boxes to complete** | **Tick as appropriate** | **Definition for type of prediction model study** |
| Development only | Development | ✔ | Prediction model development without external validation. These studies may include internal validation methods, such as bootstrapping and cross-validation techniques. |
| Development and validation | Development and validation | ✖ | Prediction model development combined with external validation in other participants in the same article. |
| Validation only | Validation | ✖ | External validation of existing (previously developed) model in other participants. |

|  | |
| --- | --- |
| **Publication reference** | Wang T, Paschalidis A, Liu Q, Liu Y, Yuan Y, Paschalidis IC. Predictive Models of Mortality for Hospitalized Patients With COVID-19: Retrospective Cohort Study. JMIR Med Inform. 2020 Oct 15;8(10):e21788. doi: 10.2196/21788. PMID: 33055061; PMCID: PMC7572117. | |
| **Models of interest** | Logistic regression and support vector machine |  |
| **Outcome of interest** | Mortality | |

**Step 3: Assess risk of bias and applicability**

| **DOMAIN 1:  Participants** | | | |
| --- | --- | --- | --- |
| **A. Risk of Bias** | | | |
| *Describe the sources of data and criteria for participant selection:*  DEV:  This retrospective study included 375 (174 deaths  (46.4%))  COVID-19 patients admitted to Tongji Hospital in Wuhan, China  between January 10 and February 18, 2020. | | | |
|  | | Dev | Val |
| 1. Were appropriate data sources used, e.g. cohort, RCT or nested case-control study data? | | Y |  |
| 1. Were all inclusions and exclusions of participants appropriate? | | PY |  |
| **Risk of bias introduced by selection of participants** | **RISK:**  *(low/ high/ unclear)* | **low** |  |
| *Rationale of bias rating:* | | | |
|  | | | |
| **B. Applicability** | | | |
| *Describe included participants, setting and dates:*  It has been mentioned in Domain 1 – Section A. | | | |
| **Concern that the included participants and setting do not match the review question** | **CONCERN:**  *(low/ high/ unclear)* | **high** |  |
| *Rationale of applicability rating:* | | | |
| The dataset that was used included a small number of patients, only 375 patients which is not enough for the prediction model development. | | | |

| **DOMAIN 2:  Predictors** | | | |
| --- | --- | --- | --- |
| **A. Risk of Bias** | | | |
| *List and describe predictors included in the final model, e.g. definition and timing of assessment:*  The dataset included epidemiological, demographic, clinical, and laboratory results.  Important predictors were identified such as lactate dehydrogenase, high-sensitivity C-reactive protein, and percentage of lymphocytes in the blood. | | | |
|  | | Dev | Val |
| 1. Were predictors defined and assessed in a similar way for all participants? | | PY |  |
| 1. Were predictor assessments made without knowledge of outcome data? | | PY |  |
| 1. Are all predictors available at the time the model is intended to be used? | | Y |  |
| **Risk of bias introduced by predictors or their assessment** | **RISK:**  *(low/ high/ unclear)* | **unclear** |  |
| *Rationale of bias rating:*  They did not use the time interval between the predictors and the outcome. | | | |
| **B. Applicability** | | | |
| Concern that the definition, assessment or timing of predictors in the model do not match the review question | **CONCERN:**  *(low/ high/ unclear)* | **high** |  |
| *Rationale of applicability rating:*  The input variables were obtained only from blood test information. There were more useful predictors from demographic and clinical data that were ignored in this study. | | | |

| **DOMAIN 3: Outcome** | | | |
| --- | --- | --- | --- |
| **A. Risk of Bias** | | | |
| *Describe the outcome, how it was defined and determined, and the time interval between predictor assessment and outcome determination:*  375 (174 deaths (46.4%))  The outcome was defined as in-hospital mortality. | | | |
|  | | Dev | Val |
| 1. Was the outcome determined appropriately? | | PY |  |
| 1. Was a pre-specified or standard outcome definition used? | | PY |  |
| 1. Were predictors excluded from the outcome definition? | | Y |  |
| 1. Was the outcome defined and determined in a similar way for all participants? | | PY |  |
| 1. Was the outcome determined without knowledge of predictor information? | | PY |  |
| 1. Was the time interval between predictor assessment and outcome determination appropriate? | | NI |  |
| **Risk of bias introduced by the outcome or its determination** | **RISK:**  *(low/ high/ unclear)* | **unclear** |  |
| *Rationale of bias rating:*  *The time interval between admission time and outcome was not defined.* | | | |
| **B. Applicability** | | | |
| *At what time point was the outcome determined:*  *If a composite outcome was used, describe the relative frequency/distribution of each contributing outcome:* | | | |
| **Concern that the outcome, its definition, timing or determination do not match the review question** | **CONCERN:**  *(low/ high/ unclear)* | **high** |  |
| *Rationale of applicability rating:*  There was no information regarding discharged patients after they left the hospital, they might have died shortly. | | | |
| **DOMAIN 4: Analysis** | | | |
| **Risk of Bias** | | | |
| *Describe numbers of participants, number of candidate predictors, outcome events and events per candidate predictor:*  DEV:  375 (174 deaths  (46.4%)) with 18 of the predictors | | | |
| *Describe how the model was developed (for example in regards to modelling technique (e.g. survival or logistic modelling), predictor selection, and risk group definition):*  logistic regression and support vector machine | | | |
| *Describe whether and how the model was validated, either internally (e.g. bootstrapping, cross validation, random split sample) or externally (e.g. temporal validation, geographical validation, different setting, different type of participants):*  Cross validation was  not used. | | | |
| *Describe the performance measures of the model, e.g. (re)calibration, discrimination, (re)classification, net benefit, and whether they were adjusted for optimism:*  The ROC analysis was not performed. | | | |
| *Describe any participants who were excluded from the analysis:*  *Data originating from pregnant and breastfeeding women or patients aged younger than 18 years and records with more than 20% missing data were excluded from the analysis [* | | | |
| *Describe missing data on predictors and outcomes as well as methods used for missing data:*  *missing data in the remaining variables were imputed using the median values of the respective variables* | | | |
|  | | Dev | Val |
| 1. Were there a reasonable number of participants with the outcome? | | N |  |
| 1. Were continuous and categorical predictors handled appropriately? | | NI |  |
| 1. Were all enrolled participants included in the analysis? | | PY |  |
| 1. Were participants with missing data handled appropriately? | | NI |  |
| 1. Was selection of predictors based on univariable analysis avoided? | | PY |  |
| 1. Were complexities in the data (e.g. censoring, competing risks, sampling of controls) accounted for appropriately? | | PY |  |
| 1. Were relevant model performance measures evaluated appropriately? | | PN |  |
| 1. Were model overfitting and optimism in model performance accounted for? | | PY |  |
| 1. Do predictors and their assigned weights in the final model correspond to the results from multivariable analysis? | | PY |  |
| **Risk of bias introduced by the analysis** | **RISK:**  *(low/ high/ unclear)* | **high** |  |
| *Rationale of bias rating:*  There was no information regarding discharged patients after they left the hospital, they might have died shortly.  Data were collected between January 10 and February 18, 2020, from patients admitted to Tongji Hospital in Wuhan, China. It was during the lockdown or Covid wave pick.  It might differ from other time periods due to the availability of therapies like steroids or vaccination. The analysis is based only on input data from hospitals in Wuhan, China without external validation. It is less clear how well the models generalize to cohorts in other countries, where patient characteristics and care practices may differ. | | | |

**Step 4: Overall assessment**

| | **Reaching an overall judgement about risk of bias of the prediction model evaluation** | | | --- | --- | | **Low risk of bias** | If all domains were rated low risk of bias.  If a prediction model was developed without any external validation, and it was rated as low risk of bias for all domains, consider downgrading to **high risk of bias**. Such a model can only be considered as low risk of bias, if the development was based on a very large data set and included some form of internal validation. | | **High risk of bias** | If at least one domain is judged to be at **high risk of bias**. | | **Unclear risk of bias** | If an unclear risk of bias was noted in at least one domain and it was low risk for all other domains. |  | **Reaching an overall judgement about applicability of the prediction model evaluation** | | | --- | --- | | **Low concerns regarding applicability** | If low concerns regarding applicability for all domains, the prediction model evaluation is judged to have **low concerns regarding applicability**. | | **High concerns regarding applicability** | If high concerns regarding applicability for at least one domain, the prediction model evaluation is judged to have **high concerns regarding applicability**. | | **Unclear concerns regarding applicability** | If unclear concerns (but no “high concern”) regarding applicability for at least one domain, the prediction model evaluation is judged to have **unclear concerns regarding applicability** overall. | |
| --- | --- | --- | --- | --- | --- | --- | --- | --- | --- | --- | --- | --- | --- | --- | --- | --- |

| **Overall judgement about risk of bias and applicability of the prediction model evaluation** | | |
| --- | --- | --- |
| **Overall judgement of risk of bias** | **RISK:**  *(low/ high/ unclear)* | **high** |
| *Summary of sources of potential bias:*  *The time interval between admission time and outcome was not defined.*  There was no information regarding discharged patients after they left the hospital, they might have died shortly. | | |
| **Overall judgement of applicability** | **CONCERN:**  *(low/ high/ unclear)* | **high** |
| *Summary of applicability concerns:*  The dataset that was used included a small number of patients, only 375 patients which is not enough for the prediction model development.  The input variables were obtained only from blood test information. There were more useful predictors from demographic and clinical data that were ignored in this study.  There was no information regarding discharged patients after they left the hospital, they might have died shortly.  Data were collected between January 10 and February 18, 2020, from patients admitted to Tongji Hospital in Wuhan, China. It was during the lockdown or Covid wave pick.  It might differ from other time periods due to the availability of therapies like steroids or vaccination. The analysis is based only on input data from hospitals in Wuhan, China without external validation. It is less clear how well the models generalize to cohorts in other countries, where patient characteristics and care practices may differ. | | |

**Appendix 16**. Baik 2020 - Bias analysis using PROBAST.

**Step 1: Specify your systematic review question**

| **Criteria** | **Specify your systematic review question** |
| --- | --- |
| *Intended use of model:* | It aims to predict patient mortality and identify its key features based on clinical characteristics and laboratory tests |
| ***Participants*** *including selection criteria and setting:* | infected COVID-19 patients |
| ***Predictors*** *(used in prediction modelling), including types of predictors (e.g. history, clinical examination, biochemical markers, imaging tests), time of measurement, specific measurement issues (e.g., any requirements/ prohibitions for specialized equipment):* | Clinical, demographics,  laboratory, medical |
| *Outcome to be predicted:* | Mortality |

**Step 2: Classify the type of prediction model evaluation**

| **Classify the evaluation based on its aim** | | | |
| --- | --- | --- | --- |
| **Type of prediction study** | **PROBAST boxes to complete** | **Tick as appropriate** | **Definition for type of prediction model study** |
| Development only | Development | ✔ | Prediction model development without external validation. These studies may include internal validation methods, such as bootstrapping and cross-validation techniques. |
| Development and validation | Development and validation | ✖ | Prediction model development combined with external validation in other participants in the same article. |
| Validation only | Validation | ✖ | External validation of existing (previously developed) model in other participants. |

|  | |
| --- | --- |
| **Publication reference** | Baik SM, Lee M, Hong KS, Park DJ. Development of Machine-Learning Model to Predict COVID-19 Mortality: Application of Ensemble Model and Regarding Feature Impacts. Diagnostics (Basel). 2022 Jun 14;12(6):1464. doi: 10.3390/diagnostics12061464. PMID: 35741274; PMCID: PMC9221552. | |
| **Models of interest** | Deep Learnig, the decision-tree models, the bagging and boosting ML models, SVM and K-nearest neighbors |  |
| **Outcome of interest** | Mortality | |

**Step 3: Assess risk of bias and applicability**

| **DOMAIN 1:  Participants** | | | |
| --- | --- | --- | --- |
| **A. Risk of Bias** | | | |
| *Describe the sources of data and criteria for participant selection:*  DEV:  This study included patients diagnosed with COVID-19 who were admitted to the sub-intensive and intensive care units between September 2021 and January 2022. All had moderate-to-severe COVID-19. Moderate severity was defined as requiring oxygen supplied by a high-flow system or mechanical ventilation. Data were collected regarding the following clinical characteristics: sex, age, medical history, vital signs, chief complaints, review of systems, and mortality  Data were also collected regarding the following laboratory data: complete blood count with differential count, prothrombin time, activated partial thromboplastin time, total calcium, phosphorus, glucose, blood urea nitrogen (BUN), creatinine, estimated glomerular filtration rate, triglyceride, total cholesterol, total protein, albumin, aspartate aminotransferase (AST), alanine aminotransferase (ALT), alkaline phosphatase, total bilirubin, sodium, potassium, chloride, total carbon dioxide (CO2), magnesium, amylase, lipase, C-reactive protein, ammonia, arterial blood gas analysis, lactate, creatinine kinase (CK), CK-MB, high sensitivity troponin T, procalcitonin, lactate dehydrogenase, N-terminal pro-B-type natri- Diagnostics 2022, 12, 1464 3 of 13 uretic peptide, uric acid, serum osmolarity, ferritin, fibrinogen, fibrinogen degradation production, and D-dimer. All clinical characteristics and laboratory findings were the initial values obtained after admission | | | |
|  | | Dev | Val |
| 1. Were appropriate data sources used, e.g. cohort, RCT or nested case-control study data? | | PY |  |
| 1. Were all inclusions and exclusions of participants appropriate? | | NI |  |
| **Risk of bias introduced by selection of participants** | **RISK:**  *(low/ high/ unclear)* | **High** |  |
| *Rationale of bias rating:* | | | |
| It is not clear how different variables in the data set were scaled, for example how laboratory values for each patient were measured which could be an average or maximum value during the assessment window. | | | |
| **B. Applicability** | | | |
| *Describe included participants, setting and dates:*  It has been mentioned in Domain 1 – Section A. | | | |
| **Concern that the included participants and setting do not match the review question** | **CONCERN:**  *(low/ high/ unclear)* | **low** |  |
| *Rationale of applicability rating:* | | | |
| In our review, our source population is the general population and their data are part of the general population. | | | |

| **DOMAIN 2:  Predictors** | | | |
| --- | --- | --- | --- |
| **A. Risk of Bias** | | | |
| *List and describe predictors included in the final model, e.g. definition and timing of assessment:*  Sex, age, medical history, vital signs, chief complaints, review of systems, mortality, complete blood count with differential count, prothrombin time, activated partial thromboplastin time, total calcium, phosphorus, glucose, blood urea nitrogen (BUN), creatinine, estimated glomerular filtration rate, triglyceride, total cholesterol, total protein, albumin, aspartate aminotransferase (AST), alanine aminotransferase (ALT), alkaline phosphatase, total bilirubin, sodium, potassium, chloride, total carbon dioxide (CO2), magnesium, amylase, lipase, C-reactive protein, ammonia, arterial blood gas analysis, lactate, creatinine kinase (CK), CK-MB, high sensitivity troponin T, procalcitonin, lactate dehydrogenase, N-terminal pro-B-type natriuretic peptide, uric acid, serum osmolarity, ferritin, fibrinogen, fibrinogen degradation production, and D-dimer. | | | |
|  | | Dev | Val |
| 1. Were predictors defined and assessed in a similar way for all participants? | | PN |  |
| 1. Were predictor assessments made without knowledge of outcome data? | | PY |  |
| 1. Are all predictors available at the time the model is intended to be used? | | PN |  |
| **Risk of bias introduced by predictors or their assessment** | **RISK:**  *(low/ high/ unclear)* | **High** |  |
| *Rationale of bias rating:*  The predictors were not defined appropriately. | | | |
| **B. Applicability** | | | |
| Concern that the definition, assessment or timing of predictors in the model do not match the review question | **CONCERN:**  *(low/ high/ unclear)* | **High** |  |
| *Rationale of applicability rating:*  The time intervals in the datasets are not defined for medical, hospital and laboratory data sets. | | | |

| **DOMAIN 3: Outcome** | | | |
| --- | --- | --- | --- |
| **A. Risk of Bias** | | | |
| *Describe the outcome, how it was defined and determined, and the time interval between predictor assessment and outcome determination:*  the outcomes is the mortality that obtained from hospital data | | | |
|  | | Dev | Val |
| 1. Was the outcome determined appropriately? | | PY |  |
| 1. Was a pre-specified or standard outcome definition used? | | PY |  |
| 1. Were predictors excluded from the outcome definition? | | Y |  |
| 1. Was the outcome defined and determined in a similar way for all participants? | | PY |  |
| 1. Was the outcome determined without knowledge of predictor information? | | PY |  |
| 1. Was the time interval between predictor assessment and outcome determination appropriate? | | N |  |
| **Risk of bias introduced by the outcome or its determination** | **RISK:**  *(low/ high/ unclear)* | **High** |  |
| *Rationale of bias rating:*  The time interval between predictor assessment and outcome determination was not defined appropriately. Perhaps for hospital data all data available during life of each patient can be useful. | | | |
| **B. Applicability** | | | |
| *At what time point was the outcome determined:*  *If a composite outcome was used, describe the relative frequency/distribution of each contributing outcome:* | | | |
| **Concern that the outcome, its definition, timing or determination do not match the review question** | **CONCERN:**  *(low/ high/ unclear)* | **high** |  |
| *Rationale of applicability rating:*  This study included patients diagnosed with COVID-19 who were admitted to the sub-intensive and intensive care units between September 2021 and January 2022, therefore the analysis lacks postdischarge follow-up data, thus it could not assess long-term mortality. | | | |
| **DOMAIN 4: Analysis** | | | |
| **Risk of Bias** | | | |
| *Describe numbers of participants, number of candidate predictors, outcome events and events per candidate predictor:*  DEV: 203 Korean patients between September 2021 and January 2022 who had moderate-to-severe COVID-19. Of these, 49 (23.1%) were in the non-survivor group, and 154 were in the survivor group (76.9%). | | | |
| *Describe how the model was developed (for example in regards to modelling technique (e.g. survival or logistic modelling), predictor selection, and risk group definition):*  Deep Learnig, the decision-tree models, the bagging and boosting ML models, SVM and K-nearest neighbors | | | |
| *Describe whether and how the model was validated, either internally (e.g. bootstrapping, cross validation, random split sample) or externally (e.g. temporal validation, geographical validation, different setting, different type of participants):*  Cross validation has been used. | | | |
| *Describe the performance measures of the model, e.g. (re)calibration, discrimination, (re)classification, net benefit, and whether they were adjusted for optimism:*  Model accuracy was reported. The ROC analysis was performed. | | | |
| *Describe any participants who were excluded from the analysis:*  Not available. | | | |
| *Describe missing data on predictors and outcomes as well as methods used for missing data:*  In this study, there were 8.0% missing values in 17,052 datasets from 203 COVID-19 cases | | | |
|  | | Dev | Val |
| 1. Were there a reasonable number of participants with the outcome? | | Y |  |
| 1. Were continuous and categorical predictors handled appropriately? | | PN |  |
| 1. Were all enrolled participants included in the analysis? | | PY |  |
| 1. Were participants with missing data handled appropriately? | | PY |  |
| 1. Was selection of predictors based on univariable analysis avoided? | | PY |  |
| 1. Were complexities in the data (e.g. censoring, competing risks, sampling of controls) accounted for appropriately? | | PY |  |
| 1. Were relevant model performance measures evaluated appropriately? | | Y |  |
| 1. Were model overfitting and optimism in model performance accounted for? | | PY |  |
| 1. Do predictors and their assigned weights in the final model correspond to the results from multivariable analysis? | | PY |  |
| **Risk of bias introduced by the analysis** | **RISK:**  *(low/ high/ unclear)* | **high** |  |
| *Rationale of bias rating:*  It is not clear how continuous and categorical predictors were handled appropriately. | | | |

**Step 4: Overall assessment**

| | **Reaching an overall judgement about risk of bias of the prediction model evaluation** | | | --- | --- | | **Low risk of bias** | If all domains were rated low risk of bias.  If a prediction model was developed without any external validation, and it was rated as low risk of bias for all domains, consider downgrading to **high risk of bias**. Such a model can only be considered as low risk of bias, if the development was based on a very large data set and included some form of internal validation. | | **High risk of bias** | If at least one domain is judged to be at **high risk of bias**. | | **Unclear risk of bias** | If an unclear risk of bias was noted in at least one domain and it was low risk for all other domains. |  | **Reaching an overall judgement about applicability of the prediction model evaluation** | | | --- | --- | | **Low concerns regarding applicability** | If low concerns regarding applicability for all domains, the prediction model evaluation is judged to have **low concerns regarding applicability**. | | **High concerns regarding applicability** | If high concerns regarding applicability for at least one domain, the prediction model evaluation is judged to have **high concerns regarding applicability**. | | **Unclear concerns regarding applicability** | If unclear concerns (but no “high concern”) regarding applicability for at least one domain, the prediction model evaluation is judged to have **unclear concerns regarding applicability** overall. | |
| --- | --- | --- | --- | --- | --- | --- | --- | --- | --- | --- | --- | --- | --- | --- | --- | --- |

| **Overall judgement about risk of bias and applicability of the prediction model evaluation** | | |
| --- | --- | --- |
| **Overall judgement of risk of bias** | **RISK:**  *(low/ high/ unclear)* | **high** |
| *Summary of sources of potential bias:*  It is not clear how different variables in the data set were scaled, for example how laboratory values for each patient were measured which could be an average or maximum value during the assessment window.  The time interval between predictor assessment and outcome determination was not defined appropriately. Perhaps for hospital data all data available during the life of each patient can be useful.  It is not clear how continuous and categorical predictors were handled appropriately. | | |
| **Overall judgement of applicability** | **CONCERN:**  *(low/ high/ unclear)* | **high** |
| *Summary of applicability concerns:*  The analysis lacks postdischarge follow-up data, thus it could not assess long-term mortality. | | |

**Appendix 17**. Wan 2022 - Bias analysis using PROBAST.

**Step 1: Specify your systematic review question**

| **Criteria** | **Specify your systematic review question** |
| --- | --- |
| *Intended use of model:* | To investigate COVID-19 mortality in patients with pre-existing health conditions and to examine the association between COVID-19 mortality and other morbidities |
| ***Participants*** *including selection criteria and setting:* | Patients positive to COVID-19. |
| ***Predictors*** *(used in prediction modelling), including types of predictors (e.g. history, clinical examination, biochemical markers, imaging tests), time of measurement, specific measurement issues (e.g., any requirements/ prohibitions for specialized equipment):* | Demographics, disease history and lifestyle factors |
| *Outcome to be predicted:* | Death following COVID-19 diagnosis. |

**Step 2: Classify the type of prediction model evaluation**

| **Classify the evaluation based on its aim** | | | |
| --- | --- | --- | --- |
| **Type of prediction study** | **PROBAST boxes to complete** | **Tick as appropriate** | **Definition for type of prediction model study** |
| Development only | Development | ✔ | Prediction model development without external validation. These studies may include internal validation methods, such as bootstrapping and cross-validation techniques. |
| Development and validation | Development and validation | ✖ | Prediction model development combined with external validation in other participants in the same article. |
| Validation only | Validation | ✖ | External validation of existing (previously developed) model in other participants. |

|  | |
| --- | --- |
| **Publication reference** | Wan TK, Huang RX, Tulu TW, Liu JD, Vodencarevic A, Wong CW, Chan KK. Identifying Predictors of COVID-19 Mortality Using Machine Learning. Life (Basel). 2022 Apr 6;12(4):547. doi: 10.3390/life12040547. PMID: 35455038; PMCID: PMC9028639. | |
| **Models of interest** | Deep Neural Networks (DNN), Random Forest Classifier (RF), eXtreme Gradient Boosting classifier (XGB) and Support Vector Machine (SVM) | |
| **Outcome of interest** | Mortality | |

**Step 3: Assess risk of bias and applicability**

| **DOMAIN 1:  Participants** | | | |
| --- | --- | --- | --- |
| **A. Risk of Bias** | | | |
| *Describe the sources of data and criteria for participant selection:*  DEV: De-identified data from 113,882 individuals, including 14,877 COVID-19 patients collected from the UK Biobank, were used in this study. | | | |
|  | | Dev | Val |
| 1. Were appropriate data sources used, e.g. cohort, RCT or nested case-control study data? | | Y |  |
| 1. Were all inclusions and exclusions of participants appropriate? | | NI |  |
| **Risk of bias introduced by selection of participants** | **RISK:**  *(low/ high/ unclear)* | *unclear* |  |
| *Rationale of bias rating:* | | | |
| It is unclear how inclusions and exclusions of participants were applied. | | | |
| **B. Applicability** | | | |
| *Describe included participants, setting and dates:*  It has been mentioned in Domain 1 – Section A. | | | |
| **Concern that the included participants and setting do not match the review question** | **CONCERN:**  *(low/ high/ unclear)* | **low** |  |
| *Rationale of applicability rating:* | | | |
| In our review, our source population is the general population which is in accordance with the data used in this study. | | | |

| **DOMAIN 2:  Predictors** | | | |
| --- | --- | --- | --- |
| **A. Risk of Bias** | | | |
| *List and describe predictors included in the final model, e.g. definition and timing of assessment:*  Age, lifestyle, illness, income, and family history | | | |
|  | | Dev | Val |
| 1. Were predictors defined and assessed in a similar way for all participants? | | Y |  |
| 1. Were predictor assessments made without knowledge of outcome data? | | Y |  |
| 1. Are all predictors available at the time the model is intended to be used? | | Y |  |
| **Risk of bias introduced by predictors or their assessment** | **RISK:**  *(low/ high/ unclear)* | **low** |  |
| *Rationale of bias rating: No Concerns.* | | | |
| **B. Applicability** | | | |
| Concern that the definition, assessment or timing of predictors in the model do not match the review question | **CONCERN:**  *(low/ high/ unclear)* | **unclear** |  |
| *Rationale of applicability rating:*  There is a big concern regarding the definition, assessment or timing of predictors in the study as they are not clear at all. | | | |

| **DOMAIN 3: Outcome** | | | |
| --- | --- | --- | --- |
| **A. Risk of Bias** | | | |
| *Describe the outcome, how it was defined and determined, and the time interval between predictor assessment and outcome determination:*  They investigated death through follow-up of all patients in the study population. | | | |
|  | | Dev | Val |
| 1. Was the outcome determined appropriately? | | PY |  |
| 1. Was a pre-specified or standard outcome definition used? | | PY |  |
| 1. Were predictors excluded from the outcome definition? | | Y |  |
| 1. Was the outcome defined and determined in a similar way for all participants? | | Y |  |
| 1. Was the outcome determined without knowledge of predictor information? | | PY |  |
| 1. Was the time interval between predictor assessment and outcome determination appropriate? | | NI |  |
| **Risk of bias introduced by the outcome or its determination** | **RISK:**  *(low/ high/ unclear)* | **high** |  |
| *Rationale of bias rating:*  There is no information regarding the time interval for the assessment of the predictors. | | | |
| **B. Applicability** | | | |
| *At what time point was the outcome determined:*  There is no information regarding the time interval for the assessment of the outcome.  *If a composite outcome was used, describe the relative frequency/distribution of each contributing outcome:* | | | |
| **Concern that the outcome, its definition, timing or determination do not match the review question** | **CONCERN:**  *(low/ high/ unclear)* | **high** |  |
| *Rationale of applicability rating:* | | | |

| **DOMAIN 4: Analysis** | | | |
| --- | --- | --- | --- |
| **Risk of Bias** | | | |
| *Describe numbers of participants, number of candidate predictors, outcome events and events per candidate predictor:*  The dataset included 17,954 features and a target variable. Of the COVID-19 patients, 799 patients died from the disease. | | | |
| *Describe how the model was developed (for example in regards to modelling technique (e.g. survival or logistic modelling), predictor selection, and risk group definition):*  Deep Neural Networks (DNN), Random Forest Classifier (RF), eXtreme Gradient Boosting classifier (XGB) and Support Vector Machine (SVM) | | | |
| *Describe whether and how the model was validated, either internally (e.g. bootstrapping, cross validation, random split sample) or externally (e.g. temporal validation, geographical validation, different setting, different type of participants):*  5-fold cross-validation | | | |
| *Describe the performance measures of the model, e.g. (re)calibration, discrimination, (re)classification, net benefit, and whether they were adjusted for optimism:*  A ROC analysis was performed. | | | |
| *Describe any participants who were excluded from the analysis:*  Not avaiable | | | |
| *Describe missing data on predictors and outcomes as well as methods used for missing data:*  Primary feature selection involved the elimination of missing data. The threshold of missing data was 30%, and 296 features were eliminated by primary feature selection. | | | |
|  | | Dev | Val |
| 1. Were there a reasonable number of participants with the outcome? | | Y |  |
| 1. Were continuous and categorical predictors handled appropriately? | | NI |  |
| 1. Were all enrolled participants included in the analysis? | | PY |  |
| 1. Were participants with missing data handled appropriately? | | NI |  |
| 1. Was selection of predictors based on univariable analysis avoided? | | Y |  |
| 1. Were complexities in the data (e.g. censoring, competing risks, sampling of controls) accounted for appropriately? | | NI |  |
| 1. Were relevant model performance measures evaluated appropriately? | | Y |  |
| 1. Were model overfitting and optimism in model performance accounted for? | | Y |  |
| 1. Do predictors and their assigned weights in the final model correspond to the results from multivariable analysis? | | NI |  |
| **Risk of bias introduced by the analysis** | **RISK:**  *(low/ high/ unclear)* | **high** |  |
| *Rationale of bias rating:*  There is great concern regarding how the missing data were handled. Moreover the parameters of the models might not be fully optimized and a higher performance could be possible  by hyperparameter tuning. There were more than a thousand features in the original dataset, many of the features could be similar. For example, age represented the highest risk for Covid-19 mortality however, more than five out of the 20 most important features were related to age. The features filtering methods might not be able to eliminate all of the related data because the correlations between them were lower than the threshold. | | | |

**Step 4: Overall assessment**

| | **Reaching an overall judgement about risk of bias of the prediction model evaluation** | | | --- | --- | | **Low risk of bias** | If all domains were rated low risk of bias.  If a prediction model was developed without any external validation, and it was rated as low risk of bias for all domains, consider downgrading to **high risk of bias**. Such a model can only be considered as low risk of bias, if the development was based on a very large data set and included some form of internal validation. | | **High risk of bias** | If at least one domain is judged to be at **high risk of bias**. | | **Unclear risk of bias** | If an unclear risk of bias was noted in at least one domain and it was low risk for all other domains. |  | **Reaching an overall judgement about applicability of the prediction model evaluation** | | | --- | --- | | **Low concerns regarding applicability** | If low concerns regarding applicability for all domains, the prediction model evaluation is judged to have **low concerns regarding applicability**. | | **High concerns regarding applicability** | If high concerns regarding applicability for at least one domain, the prediction model evaluation is judged to have **high concerns regarding applicability**. | | **Unclear concerns regarding applicability** | If unclear concerns (but no “high concern”) regarding applicability for at least one domain, the prediction model evaluation is judged to have **unclear concerns regarding applicability** overall. | |
| --- | --- | --- | --- | --- | --- | --- | --- | --- | --- | --- | --- | --- | --- | --- | --- | --- |

| **Overall judgement about risk of bias and applicability of the prediction model evaluation** | | |
| --- | --- | --- |
| **Overall judgement of risk of bias** | **RISK:**  *(low/ high/ unclear)* | **high** |
| *Summary of sources of potential bias:*  There is a big concern regarding the definition, assessment or timing of predictors in the study as they are not clear at all. There is no information regarding the time interval for the assessment of the predictors. | | |
| **Overall judgement of applicability** | **CONCERN:**  *(low/ high/ unclear)* | **high** |
| *Summary of applicability concerns:*  There is great concern regarding how the missing data were handled. Moreover the parameters of the models might not be fully optimized and a higher performance could be possible by hyperparameter tuning. There were more than a thousand features in the original dataset, many of the features could be similar. For example, age represented the highest risk for Covid-19 mortality however, more than five out of the 20 most important features were related to age. The features filtering methods might not be able to eliminate all of the related data because the correlations between them were lower than the threshold. | | |

**Appendix 18**. Park 2022 - Bias analysis using PROBAST.

**Step 1: Specify your systematic review question**

| **Criteria** | **Specify your systematic review question** |
| --- | --- |
| *Intended use of model:* | To develop a model predicting mortality rate and the severity of COVID-19 |
| ***Participants*** *including selection criteria and setting:* | Patients positive to COVID-19. |
| ***Predictors*** *(used in prediction modelling), including types of predictors (e.g. history, clinical examination, biochemical markers, imaging tests), time of measurement, specific measurement issues (e.g., any requirements/ prohibitions for specialized equipment):* | Demographic, geographic, and clinical characteristics of a superior performance |
| *Outcome to be predicted:* | *Mortality* |

**Step 2: Classify the type of prediction model evaluation**

| **Classify the evaluation based on its aim** | | | |
| --- | --- | --- | --- |
| **Type of prediction study** | **PROBAST boxes to complete** | **Tick as appropriate** | **Definition for type of prediction model study** |
| Development only | Development | ✔ | Prediction model development without external validation. These studies may include internal validation methods, such as bootstrapping and cross-validation techniques. |
| Development and validation | Development and validation | ✖ | Prediction model development combined with external validation in other participants in the same article. |
| Validation only | Validation | ✖ | External validation of existing (previously developed) model in other participants. |

|  | |
| --- | --- |
| **Publication reference** | Park MS, Jo H, Lee H, Jung SY, Hwang HJ. Machine Learning-Based COVID-19 Patients Triage Algorithm Using Patient-Generated Health Data from Nationwide Multicenter Database. Infect Dis Ther. 2022 Apr;11(2):787-805. doi: 10.1007/s40121-022-00600-4. Epub 2022 Feb 16. PMID: 35174469; PMCID: PMC8853007. | |
| **Models of interest** | XGBoost, Light GBM, Random forest, CatBoost | |
| **Outcome of interest** | Mortality | |

**Step 3: Assess risk of bias and applicability**

| **DOMAIN 1:  Participants** | | | |
| --- | --- | --- | --- |
| **A. Risk of Bias** | | | |
| *Describe the sources of data and criteria for participant selection:*  DEV: The data set was collected from February 2020 to July 2021 by the Korea Disease Control and Prevention Agency (KDCA), a government-affiliated organization, for all Koreans who tested positive for SARS-CoV-2 in polymerase chain reaction (PCR).  The data set are mainly composed of three types of patient data: (i) basic personal information, (ii) types of first symptoms, and (iii) underlying diseases. | | | |
|  | | Dev | Val |
| 1. Were appropriate data sources used, e.g. cohort, RCT or nested case-control study data? | | Y |  |
| 1. Were all inclusions and exclusions of participants appropriate? | | NI |  |
| **Risk of bias introduced by selection of participants** | **RISK:**  *(low/ high/ unclear)* | *unclear* |  |
| *Rationale of bias rating:* | | | |
| It is unclear how inclusions and exclusions of participants were applied. | | | |
| **B. Applicability** | | | |
| *Describe included participants, setting and dates:*  It has been mentioned in Domain 1 – Section A. | | | |
| **Concern that the included participants and setting do not match the review question** | **CONCERN:**  *(low/ high/ unclear)* | **low** |  |
| *Rationale of applicability rating:* | | | |
| In our review, our source population is the general population which is in accordance with the data used in this study. | | | |

| **DOMAIN 2:  Predictors** | | | |
| --- | --- | --- | --- |
| **A. Risk of Bias** | | | |
| *List and describe predictors included in the final model, e.g. definition and timing of assessment:*  Age, body temperature, renal disease, degenerative disease, cancer, liver, cardiovascular, and lung disease, dyspnea, sex, Geographic information: Higher longitude and latitude. | | | |
|  | | Dev | Val |
| 1. Were predictors defined and assessed in a similar way for all participants? | | Y |  |
| 1. Were predictor assessments made without knowledge of outcome data? | | Y |  |
| 1. Are all predictors available at the time the model is intended to be used? | | NI |  |
| **Risk of bias introduced by predictors or their assessment** | **RISK:**  *(low/ high/ unclear)* | **low** |  |
| *Rationale of bias rating:* There is a concerns regarding the time interval between predictors and outcome. | | | |
| **B. Applicability** | | | |
| Concern that the definition, assessment or timing of predictors in the model do not match the review question | **CONCERN:**  *(low/ high/ unclear)* | **unclear** |  |
| *Rationale of applicability rating:*  There is a big concern regarding the definition, assessment or timing of predictors in the study as they are not clear at all. | | | |

| **DOMAIN 3: Outcome** | | | |
| --- | --- | --- | --- |
| **A. Risk of Bias** | | | |
| *Describe the outcome, how it was defined and determined, and the time interval between predictor assessment and outcome determination:*  *The outcome was defined as deceased cases due to COVID-19 in hospitals, CTCs, and at homes. The mortality cases were collected by the KDCA from national statistics.* | | | |
|  | | Dev | Val |
| 1. Was the outcome determined appropriately? | | PY |  |
| 1. Was a pre-specified or standard outcome definition used? | | PY |  |
| 1. Were predictors excluded from the outcome definition? | | Y |  |
| 1. Was the outcome defined and determined in a similar way for all participants? | | Y |  |
| 1. Was the outcome determined without knowledge of predictor information? | | PY |  |
| 1. Was the time interval between predictor assessment and outcome determination appropriate? | | NI |  |
| **Risk of bias introduced by the outcome or its determination** | **RISK:**  *(low/ high/ unclear)* | **unclear** |  |
| *Rationale of bias rating:*  There is no information regarding the time interval for the assessment of the predictors. | | | |
| **B. Applicability** | | | |
| *At what time point was the outcome determined:*  There is no information regarding the time interval for the assessment of the outcome.  *If a composite outcome was used, describe the relative frequency/distribution of each contributing outcome:* | | | |
| **Concern that the outcome, its definition, timing or determination do not match the review question** | **CONCERN:**  *(low/ high/ unclear)* | **unclear** |  |
| *Rationale of applicability rating:* | | | |

| **DOMAIN 4: Analysis** | | | |
| --- | --- | --- | --- |
| **Risk of Bias** | | | |
| *Describe numbers of participants, number of candidate predictors, outcome events and events per candidate predictor:*  The data set consists of 149,471 patients who tested positive, of whom 2000 died. The data set is labeled according to whether the patient is dead or alive, and it is highly imbalanced (98.7% imbalance ratio). | | | |
| *Describe how the model was developed (for example in regards to modelling technique (e.g. survival or logistic modelling), predictor selection, and risk group definition):* | | | |
| *Describe whether and how the model was validated, either internally (e.g. bootstrapping, cross validation, random split sample) or externally (e.g. temporal validation, geographical validation, different setting, different type of participants):*  cross-validation was not performed | | | |
| *Describe the performance measures of the model, e.g. (re)calibration, discrimination, (re)classification, net benefit, and whether they were adjusted for optimism:*  A ROC analysis was performed. | | | |
| *Describe any participants who were excluded from the analysis:*  Not avaiable | | | |
| *Describe missing data on predictors and outcomes as well as methods used for missing data:*  there is no information regarding how missing data was handled | | | |
|  | | Dev | Val |
| 1. Were there a reasonable number of participants with the outcome? | | Y |  |
| 1. Were continuous and categorical predictors handled appropriately? | | PY |  |
| 1. Were all enrolled participants included in the analysis? | | PY |  |
| 1. Were participants with missing data handled appropriately? | | NI |  |
| 1. Was selection of predictors based on univariable analysis avoided? | | Y |  |
| 1. Were complexities in the data (e.g. censoring, competing risks, sampling of controls) accounted for appropriately? | | NI |  |
| 1. Were relevant model performance measures evaluated appropriately? | | Y |  |
| 1. Were model overfitting and optimism in model performance accounted for? | | Y |  |
| 1. Do predictors and their assigned weights in the final model correspond to the results from multivariable analysis? | | NI |  |
| **Risk of bias introduced by the analysis** | **RISK:**  *(low/ high/ unclear)* | **high** |  |
| *Rationale of bias rating:*  There is great concern regarding how the missing data were handled. They used only few machine learning models but potentially it would have been more useful to perform other more models especially linear models as baseline. | | | |

**Step 4: Overall assessment**

| | **Reaching an overall judgement about risk of bias of the prediction model evaluation** | | | --- | --- | | **Low risk of bias** | If all domains were rated low risk of bias.  If a prediction model was developed without any external validation, and it was rated as low risk of bias for all domains, consider downgrading to **high risk of bias**. Such a model can only be considered as low risk of bias, if the development was based on a very large data set and included some form of internal validation. | | **High risk of bias** | If at least one domain is judged to be at **high risk of bias**. | | **Unclear risk of bias** | If an unclear risk of bias was noted in at least one domain and it was low risk for all other domains. |  | **Reaching an overall judgement about applicability of the prediction model evaluation** | | | --- | --- | | **Low concerns regarding applicability** | If low concerns regarding applicability for all domains, the prediction model evaluation is judged to have **low concerns regarding applicability**. | | **High concerns regarding applicability** | If high concerns regarding applicability for at least one domain, the prediction model evaluation is judged to have **high concerns regarding applicability**. | | **Unclear concerns regarding applicability** | If unclear concerns (but no “high concern”) regarding applicability for at least one domain, the prediction model evaluation is judged to have **unclear concerns regarding applicability** overall. | |
| --- | --- | --- | --- | --- | --- | --- | --- | --- | --- | --- | --- | --- | --- | --- | --- | --- |

| **Overall judgement about risk of bias and applicability of the prediction model evaluation** | | |
| --- | --- | --- |
| **Overall judgement of risk of bias** | **RISK:**  *(low/ high/ unclear)* | **high** |
| *Summary of sources of potential bias:*  There is a big concern regarding the definition, assessment or timing of predictors in the study as they are not clear at all. There is no information regarding the time interval for the assessment of the predictors.  They used only few machine learning models but potentially it would have been more useful to perform other more models especially linear models as baseline. | | |
| **Overall judgement of applicability** | **CONCERN:**  *(low/ high/ unclear)* | **high** |
| *Summary of applicability concerns:*  There is great concern regarding how the missing data were handled. The study suffers from lacking cross validation for internal validation and testing new data for external validation. | | |

**Appendix 19**. Guan 2022 - Bias analysis using PROBAST.

**Step 1: Specify your systematic review question**

| **Criteria** | **Specify your systematic review question** |
| --- | --- |
| *Intended use of model:* | To appraise effective predictors for COVID-19 mortality in a retrospective cohort study |
| ***Participants*** *including selection criteria and setting:* | Patients positive to COVID-19. |
| ***Predictors*** *(used in prediction modelling), including types of predictors (e.g. history, clinical examination, biochemical markers, imaging tests), time of measurement, specific measurement issues (e.g., any requirements/ prohibitions for specialized equipment):* | Demographic, Clinical,  Laboratory |
| *Outcome to be predicted:* | Death following COVID-19 diagnosis. |

**Step 2: Classify the type of prediction model evaluation**

| **Classify the evaluation based on its aim** | | | |
| --- | --- | --- | --- |
| **Type of prediction study** | **PROBAST boxes to complete** | **Tick as appropriate** | **Definition for type of prediction model study** |
| Development only | Development | ✔ | Prediction model development without external validation. These studies may include internal validation methods, such as bootstrapping and cross-validation techniques. |
| Development and validation | Development and validation | ✖ | Prediction model development combined with external validation in other participants in the same article. |
| Validation only | Validation | ✖ | External validation of existing (previously developed) model in other participants. |

|  | |
| --- | --- |
| **Publication reference** | Guan X, Zhang B, Fu M, Li M, Yuan X, Zhu Y, Peng J, Guo H, Lu Y. Clinical and inflammatory features based machine learning model for fatal risk prediction of hospitalized COVID-19 patients: results from a retrospective cohort study. Ann Med. 2021 Dec;53(1):257-266. doi: 10.1080/07853890.2020.1868564. PMID: 33410720; PMCID: PMC7799376. | |
| **Models of interest** | Simple-tree XGBoost | |
| **Outcome of interest** | Mortality | |

**Step 3: Assess risk of bias and applicability**

| **DOMAIN 1:  Participants** | | | |
| --- | --- | --- | --- |
| **A. Risk of Bias** | | | |
| *Describe the sources of data and criteria for participant selection:*  DEV:.  the 1270 COVID-19 patients admitted in two hospitals in Wuhan between 27 January and 5 April, 2020 were enrolled in our study. Among these patients, 984 were admitted in the Sino French New City Branch of Tongji Hospital in Wuhan with recruitment period ranging from 27 January to 5 April, 2020  VAL: 286 cases were admitted in the Optical Valley Branch of Tongji Hospital in Wuhan between 3 February 2020 and 26 March 2020.  the dataset included demographic, clinical characteristics (onset symptoms, disease severity, and comorbidities), laboratory examinations (blood routine examination, cytokines and infection-related factors, blood coagulation factors, and serum biochemical index), and chest CT scan findings on admission. For some laboratory markers were below the limits of detection (LOD) among >15% subjects | | | |
|  | | Dev | Val |
| 1. Were appropriate data sources used, e.g. cohort, RCT or nested case-control study data? | | Y | PY |
| 1. Were all inclusions and exclusions of participants appropriate? | | NI | NI |
| **Risk of bias introduced by selection of participants** | **RISK:**  *(low/ high/ unclear)* | **high** | **high** |
| *Rationale of bias rating:* | | | |
| There is no information regarding covariate assessment window to include or exclude patients. | | | |
| **B. Applicability** | | | |
| *Describe included participants, setting and dates:*  It has been mentioned in Domain 1 – Section A. | | | |
| **Concern that the included participants and setting do not match the review question** | **CONCERN:**  *(low/ high/ unclear)* | **unclear** | **unclear** |
| *Rationale of applicability rating:* | | | |
| In our review, our source population is the general population. Therefore this dataset is part of the general population. | | | |

| **DOMAIN 2:  Predictors** | | | |
| --- | --- | --- | --- |
| **A. Risk of Bias** | | | |
| *List and describe predictors included in the final model, e.g. definition and timing of assessment:*  disease severity, age, levels of high-sensitivity C-reactive protein (hs-CRP), lactate dehydrogenase (LDH), ferritin, and interleukin-10 (IL-10) | | | |
|  | | Dev | Val |
| 1. Were predictors defined and assessed in a similar way for all participants? | | Y | Y |
| 1. Were predictor assessments made without knowledge of outcome data? | | Y | Y |
| 1. Are all predictors available at the time the model is intended to be used? | | Y | Y |
| **Risk of bias introduced by predictors or their assessment** | **RISK:**  *(low/ high/ unclear)* | **low** | **low** |
| *Rationale of bias rating:* | | | |
| **B. Applicability** | | | |
| Concern that the definition, assessment or timing of predictors in the model do not match the review question | **CONCERN:**  *(low/ high/ unclear)* | **high** | **high** |
| *Rationale of applicability rating:*  Predictors were assessed prior to the occurrence of the outcome. however it is not clear the time period which was used to obtain the  predictors for each patients | | | |

| **DOMAIN 3: Outcome** | | | |
| --- | --- | --- | --- |
| **A. Risk of Bias** | | | |
| *Describe the outcome, how it was defined and determined, and the time interval between predictor assessment and outcome determination:*  They investigated death in/out of hospital through follow-up of all patients in the study population. | | | |
|  | | Dev | Val |
| 1. Was the outcome determined appropriately? | | PY | PY |
| 1. Was a pre-specified or standard outcome definition used? | | PY | PN |
| 1. Were predictors excluded from the outcome definition? | | Y | Y |
| 1. Was the outcome defined and determined in a similar way for all participants? | | Y | Y |
| 1. Was the outcome determined without knowledge of predictor information? | | PY | PY |
| 1. Was the time interval between predictor assessment and outcome determination appropriate? | | NI | NI |
| **Risk of bias introduced by the outcome or its determination** | **RISK:**  *(low/ high/ unclear)* | **high** | **high** |
| *Rationale of bias rating:*  There is no information regarding in/out of hospital death through follow-up of all patients in the study population.. There is no information regarding the time interval for the assessment of the predictors and outcome. | | | |
| **B. Applicability** | | | |
| *At what time point was the outcome determined:*  There is no information regarding the time interval for the assessment of the outcome.  *If a composite outcome was used, describe the relative frequency/distribution of each contributing outcome:* | | | |
| **Concern that the outcome, its definition, timing or determination do not match the review question** | **CONCERN:**  *(low/ high/ unclear)* | **high** | **high** |
| *Rationale of applicability rating:*  There is no information regarding the time interval for the assessment *between predictor assessment and outcome determination*. | | | |

| **DOMAIN 4: Analysis** | | | |
| --- | --- | --- | --- |
| **Risk of Bias** | | | |
| *Describe numbers of participants, number of candidate predictors, outcome events and events per candidate predictor:*  DEV: 554+ 233 participants, 41+16 death 6 candidate predictors  VAL: 286 participants and 7 death 6 candidate predictors | | | |
| *Describe how the model was developed (for example in regards to modelling technique (e.g. survival or logistic modelling), predictor selection, and risk group definition):*  Simple-tree XGBoost | | | |
| *Describe whether and how the model was validated, either internally (e.g. bootstrapping, cross validation, random split sample) or externally (e.g. temporal validation, geographical validation, different setting, different type of participants):*  cross validation | | | |
| *Describe the performance measures of the model, e.g. (re)calibration, discrimination, (re)classification, net benefit, and whether they were adjusted for optimism:*  A ROC analysis was performed using a separate 2 × 2 “confusion matrix” for every possible threshold applied to the total score. The best threshold was defined by the highest value of the Youden Index. Point estimates and confidence intervals were computed using the bootstrap for sensitivity, specificity, Positive Predictive Value (PPV), Negative Predictive Value (NPV) and the De Long method for the Area Under the Curve (AUC)  Reliability analysis of the predictive formula was summarized using ROC curves showing optimal thresholds and sensitivity, specificity, AUC with point estimates and 95% confidence intervals. | | | |
| *Describe any participants who were excluded from the analysis:*  pregnant women and subjects with missing information about comorbidities, | | | |
| *Describe missing data on predictors and outcomes as well as methods used for missing data:*  Not available. | | | |
|  | | Dev | Val |
| 1. Were there a reasonable number of participants with the outcome? | | NI | NI |
| 1. Were continuous and categorical predictors handled appropriately? | | PY | PY |
| 1. Were all enrolled participants included in the analysis? | | PY | PY |
| 1. Were participants with missing data handled appropriately? | | NI | NI |
| 1. Was selection of predictors based on univariable analysis avoided? | | Y | Y |
| 1. Were complexities in the data (e.g. censoring, competing risks, sampling of controls) accounted for appropriately? | | Y | Y |
| 1. Were relevant model performance measures evaluated appropriately? | | Y | Y |
| 1. Were model overfitting and optimism in model performance accounted for? | | N | N |
| 1. Do predictors and their assigned weights in the final model correspond to the results from multivariable analysis? | | NI | NI |
| **Risk of bias introduced by the analysis** | **RISK:**  *(low/ high/ unclear)* | **high** |  |
| *Rationale of bias rating:*  Linear models such as a logistic regression model was not used to get useful information regarding the significant relevant predictors which is familiar to clinical audiences.  The sample size for external validation was too small.  They do not describe how they handled missing data. | | | |

**Step 4: Overall assessment**

| | **Reaching an overall judgement about risk of bias of the prediction model evaluation** | | | --- | --- | | **Low risk of bias** | If all domains were rated low risk of bias.  If a prediction model was developed without any external validation, and it was rated as low risk of bias for all domains, consider downgrading to **high risk of bias**. Such a model can only be considered as low risk of bias, if the development was based on a very large data set and included some form of internal validation. | | **High risk of bias** | If at least one domain is judged to be at **high risk of bias**. | | **Unclear risk of bias** | If an unclear risk of bias was noted in at least one domain and it was low risk for all other domains. |  | **Reaching an overall judgement about applicability of the prediction model evaluation** | | | --- | --- | | **Low concerns regarding applicability** | If low concerns regarding applicability for all domains, the prediction model evaluation is judged to have **low concerns regarding applicability**. | | **High concerns regarding applicability** | If high concerns regarding applicability for at least one domain, the prediction model evaluation is judged to have **high concerns regarding applicability**. | | **Unclear concerns regarding applicability** | If unclear concerns (but no “high concern”) regarding applicability for at least one domain, the prediction model evaluation is judged to have **unclear concerns regarding applicability** overall. | |
| --- | --- | --- | --- | --- | --- | --- | --- | --- | --- | --- | --- | --- | --- | --- | --- | --- |

| **Overall judgement about risk of bias and applicability of the prediction model evaluation** | | |
| --- | --- | --- |
| **Overall judgement of risk of bias** | **RISK:**  *(low/ high/ unclear)* | **high** |
| *Summary of sources of potential bias:*  Linear models such as a logistic regression model was not used to get useful information regarding the significant relevant predictors which is familiar to clinical audiences.  There is no information regarding the time interval for the assessment*between predictor assessment and outcome determination*.  They do not describe how they handled missing data. | | |
| **Overall judgement of applicability** | **CONCERN:**  *(low/ high/ unclear)* | **high** |
| *Summary of applicability concerns:*  The sample size for external validation was too small and is from the same dataset which was used for development, which might restrict the generalizability of the results. | | |

**Appendix 20**. Feng 2021 - Bias analysis using PROBAST.

**Step 1: Specify your systematic review question**

| **Criteria** | **Specify your systematic review question** |
| --- | --- |
| *Intended use of model:* | To assess the predictive accuracy of machine learning methods to predict the COVID-19 mortality risk |
| ***Participants*** *including selection criteria and setting:* | positive tested COVID-19 patients |
| ***Predictors*** *(used in prediction modelling), including types of predictors (e.g. history, clinical examination, biochemical markers, imaging tests), time of measurement, specific measurement issues (e.g., any requirements/ prohibitions for specialized equipment):* | Demographic, Clinical, Laboratory, Social and Economic, daily temperature |
| *Outcome to be predicted:* | Mortality |

**Step 2: Classify the type of prediction model evaluation**

| **Classify the evaluation based on its aim** | | | |
| --- | --- | --- | --- |
| **Type of prediction study** | **PROBAST boxes to complete** | **Tick as appropriate** | **Definition for type of prediction model study** |
| Development only | Development | ✔ | Prediction model development without external validation. These studies may include internal validation methods, such as bootstrapping and cross-validation techniques. |
| Development and validation | Development and validation | ✖ | Prediction model development combined with external validation in other participants in the same article. |
| Validation only | Validation | ✖ | External validation of existing (previously developed) model in other participants. |

|  | |
| --- | --- |
| **Publication reference** | Feng C, Kephart G, Juarez-Colunga E. Predicting COVID-19 mortality risk in Toronto, Canada: a comparison of tree-based and regression-based machine learning methods. BMC Med Res Methodol. 2021 Nov 27;21(1):267. doi: 10.1186/s12874-021-01441-4. PMID: 34837951; PMCID: PMC8627169. | |
| **Models of interest** | classification tree, random forest (RF), extreme gradient boosting (XGBoost), logistic regression, generalized additive model (GAM) and linear discriminant analysis (LDA) |  |
| **Outcome of interest** | Mortality | |

**Step 3: Assess risk of bias and applicability**

| **DOMAIN 1:  Participants** | | | |
| --- | --- | --- | --- |
| **A. Risk of Bias** | | | |
| *Describe the sources of data and criteria for participant selection:*  DEV:  Demographic, Clinical and Laboratory datasets of 49,216 COVID-19 positive cases in Toronto, Canada, between March 1 to December 10, 2020 were obtained from the Ontario Ministry of Health. Social and Economic was obtained from the 2016 Canadian Census data, and daily temperature was downloaded from the Government of Canada Daily Weather Data Report. | | | |
|  | | Dev | Val |
| 1. Were appropriate data sources used, e.g. cohort, RCT or nested case-control study data? | | Y |  |
| 1. Were all inclusions and exclusions of participants appropriate? | | NI |  |
| **Risk of bias introduced by selection of participants** | **RISK:**  *(low/ high/ unclear)* | **low** |  |
| *Rationale of bias rating:* | | | |
|  | | | |
| **B. Applicability** | | | |
| *Describe included participants, setting and dates:*  It has been mentioned in Domain 1 – Section A. | | | |
| **Concern that the included participants and setting do not match the review question** | **CONCERN:**  *(low/ high/ unclear)* | **low** |  |
| *Rationale of applicability rating:* | | | |
| In our review, our source population is the general population. Therefore, this data is part of the general population. | | | |

| **DOMAIN 2:  Predictors** | | | |
| --- | --- | --- | --- |
| **A. Risk of Bias** | | | |
| *List and describe predictors included in the final model, e.g. definition and timing of assessment:*  Age, Gender, Ever Hospitalized, Ever in ICU, Ever Intubated, Population Density, Average Income, Daily Mean Temperature | | | |
|  | | Dev | Val |
| 1. Were predictors defined and assessed in a similar way for all participants? | | PY |  |
| 1. Were predictor assessments made without knowledge of outcome data? | | PY |  |
| 1. Are all predictors available at the time the model is intended to be used? | | Y |  |
| **Risk of bias introduced by predictors or their assessment** | **RISK:**  *(low/ high/ unclear)* | **high** |  |
| *Rationale of bias rating:*  *The covariant assessment window was not defined to generate variables. For example, for average income, even if we assume it is* between March 1 to December 10, 2020 like covid-19 dataset, it may not be suitable. | | | |
| **B. Applicability** | | | |
| Concern that the definition, assessment or timing of predictors in the model do not match the review question | **CONCERN:**  *(low/ high/ unclear)* | **high** |  |
| *Rationale of applicability rating:*  There could be correlation between some strong variables such as population density and average income and between income and ability to have an access to healthcare units. | | | |

| **DOMAIN 3: Outcome** | | | |
| --- | --- | --- | --- |
| **A. Risk of Bias** | | | |
| *Describe the outcome, how it was defined and determined, and the time interval between predictor assessment and outcome determination:*  The time interval between predictors assessment and outcome was evaluated by predictive ability of the  ML models.  It was defined from the last 7 to 30 days at the end of the observational period. The results indicate that the accuracy of all the methods tends to decrease as the number of forecast days increases. | | | |
|  | | Dev | Val |
| 1. Was the outcome determined appropriately? | | PY |  |
| 1. Was a pre-specified or standard outcome definition used? | | PY |  |
| 1. Were predictors excluded from the outcome definition? | | Y |  |
| 1. Was the outcome defined and determined in a similar way for all participants? | | PY |  |
| 1. Was the outcome determined without knowledge of predictor information? | | PY |  |
| 1. Was the time interval between predictor assessment and outcome determination appropriate? | | PY |  |
| **Risk of bias introduced by the outcome or its determination** | **RISK:**  *(low/ high/ unclear)* | **unclear** |  |
| *Rationale of bias rating:*  The time interval between predictors assessment and outcome was evaluated by predictive ability of the  ML models.  It was defined from the last 7 to 30 days at the end of the observational period. The results indicate that the accuracy of all the methods tends to decrease as the number of forecast days increases. There was no reference for choosing this time interval. | | | |
| **B. Applicability** | | | |
| *At what time point was the outcome determined:*  *If a composite outcome was used, describe the relative frequency/distribution of each contributing outcome:* | | | |
| **Concern that the outcome, its definition, timing or determination do not match the review question** | **CONCERN:**  *(low/ high/ unclear)* | **unclear** |  |
| *Rationale of applicability rating:*  Having different time intervals between predictors assessment and outcome might cause different results in different settings. | | | |
| **DOMAIN 4: Analysis** | | | |
| **Risk of Bias** | | | |
| *Describe numbers of participants, number of candidate predictors, outcome events and events per candidate predictor:*  DEV:  49,216 COVID-19 positive cases, 1938 (3.9%) deaths | | | |
| *Describe how the model was developed (for example in regards to modelling technique (e.g. survival or logistic modelling), predictor selection, and risk group definition):*  classification tree, random forest (RF), extreme gradient boosting (XGBoost), logistic regression, generalized additive model (GAM) and linear discriminant analysis (LDA) | | | |
| *Describe whether and how the model was validated, either internally (e.g. bootstrapping, cross validation, random split sample) or externally (e.g. temporal validation, geographical validation, different setting, different type of participants):*  Cross validation was used. | | | |
| *Describe the performance measures of the model, e.g. (re)calibration, discrimination, (re)classification, net benefit, and whether they were adjusted for optimism:*  The ROC analysis was performed. | | | |
| *Describe any participants who were excluded from the analysis:*  *NI* | | | |
| *Describe missing data on predictors and outcomes as well as methods used for missing data:*  *NI* | | | |
|  | | Dev | Val |
| 1. Were there a reasonable number of participants with the outcome? | | PY |  |
| 1. Were continuous and categorical predictors handled appropriately? | | PY |  |
| 1. Were all enrolled participants included in the analysis? | | PY |  |
| 1. Were participants with missing data handled appropriately? | | NI |  |
| 1. Was selection of predictors based on univariable analysis avoided? | | NI |  |
| 1. Were complexities in the data (e.g. censoring, competing risks, sampling of controls) accounted for appropriately? | | PY |  |
| 1. Were relevant model performance measures evaluated appropriately? | | PN |  |
| 1. Were model overfitting and optimism in model performance accounted for? | | PY |  |
| 1. Do predictors and their assigned weights in the final model correspond to the results from multivariable analysis? | | PY |  |
| **Risk of bias introduced by the analysis** | **RISK:**  *(low/ high/ unclear)* | **high** |  |
| *Rationale of bias rating:*  The analysis is based on input data only from the region of Ontario. Therefore the model may not be generalized to other settings.  The analysis was performed without using clinical characteristics of patients, such as comorbidities. | | | |

**Step 4: Overall assessment**

| | **Reaching an overall judgement about risk of bias of the prediction model evaluation** | | | --- | --- | | **Low risk of bias** | If all domains were rated low risk of bias.  If a prediction model was developed without any external validation, and it was rated as low risk of bias for all domains, consider downgrading to **high risk of bias**. Such a model can only be considered as low risk of bias, if the development was based on a very large data set and included some form of internal validation. | | **High risk of bias** | If at least one domain is judged to be at **high risk of bias**. | | **Unclear risk of bias** | If an unclear risk of bias was noted in at least one domain and it was low risk for all other domains. |  | **Reaching an overall judgement about applicability of the prediction model evaluation** | | | --- | --- | | **Low concerns regarding applicability** | If low concerns regarding applicability for all domains, the prediction model evaluation is judged to have **low concerns regarding applicability**. | | **High concerns regarding applicability** | If high concerns regarding applicability for at least one domain, the prediction model evaluation is judged to have **high concerns regarding applicability**. | | **Unclear concerns regarding applicability** | If unclear concerns (but no “high concern”) regarding applicability for at least one domain, the prediction model evaluation is judged to have **unclear concerns regarding applicability** overall. | |
| --- | --- | --- | --- | --- | --- | --- | --- | --- | --- | --- | --- | --- | --- | --- | --- | --- |

| **Overall judgement about risk of bias and applicability of the prediction model evaluation** | | |
| --- | --- | --- |
| **Overall judgement of risk of bias** | **RISK:**  *(low/ high/ unclear)* | **high** |
| *Summary of sources of potential bias:*  *The covariant assessment window was not defined to generate variables. For example, for average income, even if we assume it is* between March 1 to December 10, 2020, like covid-19 dataset, it may not be suitable.  The time interval between predictors assessment and outcome was evaluated by predictive ability of the  ML models.  It was defined from the last 7 to 30 days at the end of the observational period. The results indicate that the accuracy of all the methods tends to decrease as the number of forecast days increases. There was no reference for choosing this time interval.  The analysis was performed without using clinical characteristics of patients, such as comorbidities. | | |
| **Overall judgement of applicability** | **CONCERN:**  *(low/ high/ unclear)* | **high** |
| *Summary of applicability concerns:*  There could be correlation between some strong variables such as population density and average income and between income and ability to have an access to healthcare units.  Having different time intervals between predictors assessment and outcome might cause different results in different settings.  The analysis is based on input data only from the region of Ontario without external validation. Therefore the model may not be generalized to other settings. | | |

**Appendix 21**. Murri 2021 - Bias analysis using PROBAST.

**Step 1: Specify your systematic review question**

| **Criteria** | **Specify your systematic review question** |
| --- | --- |
| *Intended use of model:* | To develop a machine-learning risk prediction model using easy-to-obtain parameters to identify COVID-19 patients with  higher risk of death. |
| ***Participants*** *including selection criteria and setting:* | Positive tested COVID-19 patients |
| ***Predictors*** *(used in prediction modelling), including types of predictors (e.g. history, clinical examination, biochemical markers, imaging tests), time of measurement, specific measurement issues (e.g., any requirements/ prohibitions for specialized equipment):* | Demographics, comorbidities, vital signs, laboratory characteristics,  medical history, symptoms at onset, treatment, and outcome data on admission and during hospitalization |
| *Outcome to be predicted:* | Mortality |

**Step 2: Classify the type of prediction model evaluation**

| **Classify the evaluation based on its aim** | | | |
| --- | --- | --- | --- |
| **Type of prediction study** | **PROBAST boxes to complete** | **Tick as appropriate** | **Definition for type of prediction model study** |
| Development only | Development | ✔ | Prediction model development without external validation. These studies may include internal validation methods, such as bootstrapping and cross-validation techniques. |
| Development and validation | Development and validation | ✖ | Prediction model development combined with external validation in other participants in the same article. |
| Validation only | Validation | ✖ | External validation of existing (previously developed) model in other participants. |

|  | |
| --- | --- |
| **Publication reference** | Murri R, Lenkowicz J, Masciocchi C, Iacomini C, Fantoni M, Damiani A, Marchetti A, Sergi PDA, Arcuri G, Cesario A, Patarnello S, Antonelli M, Bellantone R, Bernabei R, Boccia S, Calabresi P, Cambieri A, Cauda R, Colosimo C, Crea F, De Maria R, De Stefano V, Franceschi F, Gasbarrini A, Parolini O, Richeldi L, Sanguinetti M, Urbani A, Zega M, Scambia G, Valentini V; Gemelli against Covid Group. A machine-learning parsimonious multivariable predictive model of mortality risk in patients with Covid-19. Sci Rep. 2021 Oct 27;11(1):21136. doi: 10.1038/s41598-021-99905-6. PMID: 34707184; PMCID: PMC8551240. | |
| **Models of interest** | Logistic regression |  |
| **Outcome of interest** | Mortality | |

**Step 3: Assess risk of bias and applicability**

| **DOMAIN 1:  Participants** | | | |
| --- | --- | --- | --- |
| **A. Risk of Bias** | | | |
| *Describe the sources of data and criteria for participant selection:*  DEV:  The dataset was obtained from all patients admitted to Fondazione Policlinico Gemelli with COVID-19 and divided to train and test datasets. The training cohort included 921 patients (120 died (13%)) from March 5, 2020, to November 5, 2020 while testing cohort included 1463 (with 22.6% death) patients from November 6, 2020, to February 5, 2021. | | | |
|  | | Dev | Val |
| 1. Were appropriate data sources used, e.g. cohort, RCT or nested case-control study data? | | Y |  |
| 1. Were all inclusions and exclusions of participants appropriate? | | NI |  |
| **Risk of bias introduced by selection of participants** | **RISK:**  *(low/ high/ unclear)* | **unclear** |  |
| *Rationale of bias rating:* | | | |
| inclusions and excursions of participants are not clear. | | | |
| **B. Applicability** | | | |
| *Describe included participants, setting and dates:*  It has been mentioned in Domain 1 – Section A. | | | |
| **Concern that the included participants and setting do not match the review question** | **CONCERN:**  *(low/ high/ unclear)* | **low** |  |
| *Rationale of applicability rating:* | | | |
| In our review, our source population is the general population. Therefore, this data is part of the general population. | | | |

| **DOMAIN 2:  Predictors** | | | |
| --- | --- | --- | --- |
| **A. Risk of Bias** | | | |
| *List and describe predictors included in the final model, e.g. definition and timing of assessment:*  Age, platelet count, SpO2, blood urea nitrogen (BUN), hemoglobin, C-reactive protein, neutrophil count, and sodium. | | | |
|  | | Dev | Val |
| 1. Were predictors defined and assessed in a similar way for all participants? | | PY |  |
| 1. Were predictor assessments made without knowledge of outcome data? | | PY |  |
| 1. Are all predictors available at the time the model is intended to be used? | | Y |  |
| **Risk of bias introduced by predictors or their assessment** | **RISK:**  *(low/ high/ unclear)* | **high** |  |
| *Rationale of bias rating:*  *The covariant assessment window was not defined to generate variables. For example, for* comorbidities*, even if we assume it was* between November 6, 2020 to February 5, it might not be suitable to check all chronic diseases during the life of a patients. | | | |
| **B. Applicability** | | | |
| Concern that the definition, assessment or timing of predictors in the model do not match the review question | **CONCERN:**  *(low/ high/ unclear)* | **high** |  |
| *Rationale of applicability rating:*  When the covariate assessment window was not defined for predictors, it could cause limited reliability of the results and its applicability | | | |

| **DOMAIN 3: Outcome** | | | |
| --- | --- | --- | --- |
| **A. Risk of Bias** | | | |
| *Describe the outcome, how it was defined and determined, and the time interval between predictor assessment and outcome determination:*  The primary outcome was in-hospital case-fatality rate. | | | |
|  | | Dev | Val |
| 1. Was the outcome determined appropriately? | | PY |  |
| 1. Was a pre-specified or standard outcome definition used? | | PY |  |
| 1. Were predictors excluded from the outcome definition? | | Y |  |
| 1. Was the outcome defined and determined in a similar way for all participants? | | PY |  |
| 1. Was the outcome determined without knowledge of predictor information? | | PY |  |
| 1. Was the time interval between predictor assessment and outcome determination appropriate? | | NI |  |
| **Risk of bias introduced by the outcome or its determination** | **RISK:**  *(low/ high/ unclear)* | **high** |  |
| *Rationale of bias rating:*  The time interval between predictors’ assessment and outcome was not defined. | | | |
| **B. Applicability** | | | |
| *At what time point was the outcome determined:*  *If a composite outcome was used, describe the relative frequency/distribution of each contributing outcome:* | | | |
| **Concern that the outcome, its definition, timing or determination do not match the review question** | **CONCERN:**  *(low/ high/ unclear)* | **high** |  |
| *Rationale of applicability rating:*  having different time intervals between predictors assessment and outcome might cause different results in different settings. | | | |
| **DOMAIN 4: Analysis** | | | |
| **Risk of Bias** | | | |
| *Describe numbers of participants, number of candidate predictors, outcome events and events per candidate predictor:*  DEV:   training: 921 patients (120 died (13%))  testing: 1463 ( 22.6% death) | | | |
| *Describe how the model was developed (for example in regards to modelling technique (e.g. survival or logistic modelling), predictor selection, and risk group definition):*  logistic modelling | | | |
| *Describe whether and how the model was validated, either internally (e.g. bootstrapping, cross validation, random split sample) or externally (e.g. temporal validation, geographical validation, different setting, different type of participants):*  Cross validation was used. | | | |
| *Describe the performance measures of the model, e.g. (re)calibration, discrimination, (re)classification, net benefit, and whether they were adjusted for optimism:*  The ROC analysis was performed. | | | |
| *Describe any participants who were excluded from the analysis:*  *NI* | | | |
| *Describe missing data on predictors and outcomes as well as methods used for missing data:*  *NI* | | | |
|  | | Dev | Val |
| 1. Were there a reasonable number of participants with the outcome? | | PY |  |
| 1. Were continuous and categorical predictors handled appropriately? | | NI |  |
| 1. Were all enrolled participants included in the analysis? | | PY |  |
| 1. Were participants with missing data handled appropriately? | | NI |  |
| 1. Was selection of predictors based on univariable analysis avoided? | | NI |  |
| 1. Were complexities in the data (e.g. censoring, competing risks, sampling of controls) accounted for appropriately? | | PY |  |
| 1. Were relevant model performance measures evaluated appropriately? | | PN |  |
| 1. Were model overfitting and optimism in model performance accounted for? | | PY |  |
| 1. Do predictors and their assigned weights in the final model correspond to the results from multivariable analysis? | | PY |  |
| **Risk of bias introduced by the analysis** | **RISK:**  *(low/ high/ unclear)* | **high** |  |
| *Rationale of bias rating:*  The model was not evaluated with external validation. The analysis is based on input data only from Fondazione Policlinico Gemelli. Therefore the model may not be generalized to other settings. | | | |

**Step 4: Overall assessment**

| | **Reaching an overall judgement about risk of bias of the prediction model evaluation** | | | --- | --- | | **Low risk of bias** | If all domains were rated low risk of bias.  If a prediction model was developed without any external validation, and it was rated as low risk of bias for all domains, consider downgrading to **high risk of bias**. Such a model can only be considered as low risk of bias, if the development was based on a very large data set and included some form of internal validation. | | **High risk of bias** | If at least one domain is judged to be at **high risk of bias**. | | **Unclear risk of bias** | If an unclear risk of bias was noted in at least one domain and it was low risk for all other domains. |  | **Reaching an overall judgement about applicability of the prediction model evaluation** | | | --- | --- | | **Low concerns regarding applicability** | If low concerns regarding applicability for all domains, the prediction model evaluation is judged to have **low concerns regarding applicability**. | | **High concerns regarding applicability** | If high concerns regarding applicability for at least one domain, the prediction model evaluation is judged to have **high concerns regarding applicability**. | | **Unclear concerns regarding applicability** | If unclear concerns (but no “high concern”) regarding applicability for at least one domain, the prediction model evaluation is judged to have **unclear concerns regarding applicability** overall. | |
| --- | --- | --- | --- | --- | --- | --- | --- | --- | --- | --- | --- | --- | --- | --- | --- | --- |

| **Overall judgement about risk of bias and applicability of the prediction model evaluation** | | |
| --- | --- | --- |
| **Overall judgement of risk of bias** | **RISK:**  *(low/ high/ unclear)* | **high** |
| *Summary of sources of potential bias:*  The covariant assessment window was not defined to generate variables. For example, for comorbidities, even if we assume it was between November 6, 2020 to February 5, it might not be suitable to check all chronic diseases during the life of a patients.  The time interval between predictors’ assessment and outcome was not defined. | | |
| **Overall judgement of applicability** | **CONCERN:**  *(low/ high/ unclear)* | **high** |
| *Summary of applicability concerns:*  When the covariate assessment window was not defined for predictors, it could cause limited reliability of the results and its applicability having different time intervals between predictors assessment and outcome might cause different results in different settings.  The model was not evaluated with external validation. The analysis is based on input data only from Fondazione Policlinico Gemelli. Therefore the model may not be generalizable to other settings. | | |

**Appendix 22**. Tabatabaie 2021 - Bias analysis using PROBAST.

**Step 1: Specify your systematic review question**

| **Criteria** | **Specify your systematic review question** |
| --- | --- |
| *Intended use of model:* | To predict patients’ mortality with COVID-19 who had been already  admitted to a hospital using the basic information on the first day of admission. |
| ***Participants*** *including selection criteria and setting:* | Positive tested COVID-19 patients and admitted to a hospital |
| ***Predictors*** *(used in prediction modelling), including types of predictors (e.g. history, clinical examination, biochemical markers, imaging tests), time of measurement, specific measurement issues (e.g., any requirements/ prohibitions for specialized equipment):* | Demographic, clinical, laboratory, medicine |
| *Outcome to be predicted:* | Mortality |

**Step 2: Classify the type of prediction model evaluation**

| **Classify the evaluation based on its aim** | | | |
| --- | --- | --- | --- |
| **Type of prediction study** | **PROBAST boxes to complete** | **Tick as appropriate** | **Definition for type of prediction model study** |
| Development only | Development | ✔ | Prediction model development without external validation. These studies may include internal validation methods, such as bootstrapping and cross-validation techniques. |
| Development and validation | Development and validation | ✖ | Prediction model development combined with external validation in other participants in the same article. |
| Validation only | Validation | ✖ | External validation of existing (previously developed) model in other participants. |

|  | |
| --- | --- |
| **Publication reference** | Tabatabaie M, Sarrami AH, Didehdar M, Tasorian B, Shafaat O, Sotoudeh H. Accuracy of Machine Learning Models to Predict Mortality in COVID-19 Infection Using the Clinical and Laboratory Data at the Time of Admission. Cureus. 2021 Oct 14;13(10):e18768. doi: 10.7759/cureus.18768. PMID: 34804648; PMCID: PMC8592290. | |
| **Models of interest** | K-nearest neighbors, decision tree, random forest, support vector machine, naive Bayes, AdaBoost, and neural network |  |
| **Outcome of interest** | Mortality | |

**Step 3: Assess risk of bias and applicability**

| **DOMAIN 1:  Participants** | | | |
| --- | --- | --- | --- |
| **A. Risk of Bias** | | | |
| *Describe the sources of data and criteria for participant selection:*  DEV:  The data of the 616 patients, aged above 18 years admitted to a tertiary referral hospital from March 20, 2020 to February 3, 2021 with a clinical diagnosis of COVID-19 infection were included. Multiple features related to the patient's demographic, medical history, and clinical and laboratory findings “at the time of admission” and discharge or death **during the admission** as the outcome were extracted.  520  patients (250 patients died) with a mean age of 67.48 years admitted from March 20, 2020 to November 20, 2020  and 94 patients (13 patients died) with a mean age of 59.45 years admitted from November 21, 2020, to February 3, 2021 were included in training and testing data respectively. | | | |
|  | | Dev | Val |
| 1. Were appropriate data sources used, e.g. cohort, RCT or nested case-control study data? | | Y |  |
| 1. Were all inclusions and exclusions of participants appropriate? | | NI |  |
| **Risk of bias introduced by selection of participants** | **RISK:**  *(low/ high/ unclear)* | **unclear** |  |
| *Rationale of bias rating:* | | | |
| Inclusions and excursions of participants are not clear. It includes old adults. | | | |
| **B. Applicability** | | | |
| *Describe included participants, setting and dates:*  It has been mentioned in Domain 1 – Section A. | | | |
| **Concern that the included participants and setting do not match the review question** | **CONCERN:**  *(low/ high/ unclear)* | **low** |  |
| *Rationale of applicability rating:* | | | |
| In our review, our source population is the general population. Therefore, this data is part of the general population. | | | |

| **DOMAIN 2:  Predictors** | | | |
| --- | --- | --- | --- |
| **A. Risk of Bias** | | | |
| *List and describe predictors included in the final model, e.g. definition and timing of assessment:*  saturation of O2 (O2sat), lactate dehydrogenase (LDH), age, blood urea nitrogen (BUN), base excess, creatinine, base excess in the extracellular fluid compartment, white blood cell (WBC) count, HCO3, the result of PCR test, blood sugar, bilirubin total, myalgia, monocyte count, respiratory rate, bilirubin direct, the time between initial symptoms and admission (days), nausea, temperature, eosinophil count, Aspartate aminotransferase (SGOT), and dyspnea. | | | |
|  | | Dev | Val |
| 1. Were predictors defined and assessed in a similar way for all participants? | | PY |  |
| 1. Were predictor assessments made without knowledge of outcome data? | | PY |  |
| 1. Are all predictors available at the time the model is intended to be used? | | Y |  |
| **Risk of bias introduced by predictors or their assessment** | **RISK:**  *(low/ high/ unclear)* | **high** |  |
| *Rationale of bias rating:*  *The covariant assessment window was not defined to generate variables. a limited timing for assessment on the first day of admission* | | | |
| **B. Applicability** | | | |
| Concern that the definition, assessment or timing of predictors in the model do not match the review question | **CONCERN:**  *(low/ high/ unclear)* | **high** |  |
| *Rationale of applicability rating:*  When the covariate assessment window was not defined for predictors, it could cause limited reliability of the results and its applicability. we supposed the assessment should be done *on the first day of admission which is a limited timing for assessment and may not be applicable in clinical setting to predict mortality.* | | | |

| **DOMAIN 3: Outcome** | | | |
| --- | --- | --- | --- |
| **A. Risk of Bias** | | | |
| *Describe the outcome, how it was defined and determined, and the time interval between predictor assessment and outcome determination:*  The primary outcome was in-hospital case-fatality rate. | | | |
|  | | Dev | Val |
| 1. Was the outcome determined appropriately? | | PY |  |
| 1. Was a pre-specified or standard outcome definition used? | | PY |  |
| 1. Were predictors excluded from the outcome definition? | | Y |  |
| 1. Was the outcome defined and determined in a similar way for all participants? | | PY |  |
| 1. Was the outcome determined without knowledge of predictor information? | | PY |  |
| 1. Was the time interval between predictor assessment and outcome determination appropriate? | | NI |  |
| **Risk of bias introduced by the outcome or its determination** | **RISK:**  *(low/ high/ unclear)* | **high** |  |
| *Rationale of bias rating:*  The time interval between predictors’ assessment and outcome was not defined. | | | |
| **B. Applicability** | | | |
| *At what time point was the outcome determined:*  *If a composite outcome was used, describe the relative frequency/distribution of each contributing outcome:* | | | |
| **Concern that the outcome, its definition, timing or determination do not match the review question** | **CONCERN:**  *(low/ high/ unclear)* | **high** |  |
| *Rationale of applicability rating:*  Having different time intervals between predictors’ assessment and outcome might cause different results in different settings. | | | |
| **DOMAIN 4: Analysis** | | | |
| **Risk of Bias** | | | |
| *Describe numbers of participants, number of candidate predictors, outcome events and events per candidate predictor:*  DEV:   training: 520patients (250 died)  testing: 64( 13% death) | | | |
| *Describe how the model was developed (for example in regards to modelling technique (e.g. survival or logistic modelling), predictor selection, and risk group definition):*  lk-nearest neighbors, decision tree, random forest, support vector machine, naive Bayes, AdaBoost, and neural network | | | |
| *Describe whether and how the model was validated, either internally (e.g. bootstrapping, cross validation, random split sample) or externally (e.g. temporal validation, geographical validation, different setting, different type of participants):*  Cross validation was used. | | | |
| *Describe the performance measures of the model, e.g. (re)calibration, discrimination, (re)classification, net benefit, and whether they were adjusted for optimism:*  The ROC analysis was performed. | | | |
| *Describe any participants who were excluded from the analysis:*  *The patients who left the hospital against the physicians’ recommendation were excluded.* | | | |
| *Describe missing data on predictors and outcomes as well as methods used for missing data:*  *NI* | | | |
|  | | Dev | Val |
| 1. Were there a reasonable number of participants with the outcome? | | PY |  |
| 1. Were continuous and categorical predictors handled appropriately? | | NI |  |
| 1. Were all enrolled participants included in the analysis? | | PY |  |
| 1. Were participants with missing data handled appropriately? | | NI |  |
| 1. Was selection of predictors based on univariable analysis avoided? | | NI |  |
| 1. Were complexities in the data (e.g. censoring, competing risks, sampling of controls) accounted for appropriately? | | PY |  |
| 1. Were relevant model performance measures evaluated appropriately? | | PN |  |
| 1. Were model overfitting and optimism in model performance accounted for? | | PY |  |
| 1. Do predictors and their assigned weights in the final model correspond to the results from multivariable analysis? | | PY |  |
| **Risk of bias introduced by the analysis** | **RISK:**  *(low/ high/ unclear)* | **high** |  |
| *Rationale of bias rating:*  The model was not evaluated with external validation. The analysis is based on input data only from an unknown hospital. Therefore the model may not be generalized to other settings. | | | |

**Step 4: Overall assessment**

| | **Reaching an overall judgement about risk of bias of the prediction model evaluation** | | | --- | --- | | **Low risk of bias** | If all domains were rated low risk of bias.  If a prediction model was developed without any external validation, and it was rated as low risk of bias for all domains, consider downgrading to **high risk of bias**. Such a model can only be considered as low risk of bias, if the development was based on a very large data set and included some form of internal validation. | | **High risk of bias** | If at least one domain is judged to be at **high risk of bias**. | | **Unclear risk of bias** | If an unclear risk of bias was noted in at least one domain and it was low risk for all other domains. |  | **Reaching an overall judgement about applicability of the prediction model evaluation** | | | --- | --- | | **Low concerns regarding applicability** | If low concerns regarding applicability for all domains, the prediction model evaluation is judged to have **low concerns regarding applicability**. | | **High concerns regarding applicability** | If high concerns regarding applicability for at least one domain, the prediction model evaluation is judged to have **high concerns regarding applicability**. | | **Unclear concerns regarding applicability** | If unclear concerns (but no “high concern”) regarding applicability for at least one domain, the prediction model evaluation is judged to have **unclear concerns regarding applicability** overall. | |
| --- | --- | --- | --- | --- | --- | --- | --- | --- | --- | --- | --- | --- | --- | --- | --- | --- |

| **Overall judgement about risk of bias and applicability of the prediction model evaluation** | | |
| --- | --- | --- |
| **Overall judgement of risk of bias** | **RISK:**  *(low/ high/ unclear)* | **high** |
| *Summary of sources of potential bias:*  When the covariate assessment window was not defined for predictors, it could cause limited reliability of the results and its applicability. we supposed the assessment should be done *on the first day of admission which is a limited timing for assessment and may not be applicable in clinical setting to predict mortality.*  The time interval between predictors’ assessment and outcome was not defined. | | |
| **Overall judgement of applicability** | **CONCERN:**  *(low/ high/ unclear)* | **high** |
| *Summary of applicability concerns:*  When the covariate assessment window was not defined for predictors, it could cause limited reliability of the results and its applicability  Having different time intervals between predictors assessment and outcome might cause different results in different settings.  The model was not evaluated with external validation. The analysis is based on input data only from an unknown hospital. Therefore the model may not be generalized to other settings. | | |

**Appendix 23**. Moulaei 2021 - Bias analysis using PROBAST.

**Step 1: Specify your systematic review question**

| **Criteria** | **Specify your systematic review question** |
| --- | --- |
| *Intended use of model:* | To predict COVID-19 patients' mortality  who had already been hospitalized based on data mining techniques. |
| ***Participants*** *including selection criteria and setting:* | Positive tested COVID-19 patients and admitted to a hospital |
| ***Predictors*** *(used in prediction modelling), including types of predictors (e.g. history, clinical examination, biochemical markers, imaging tests), time of measurement, specific measurement issues (e.g., any requirements/ prohibitions for specialized equipment):* | Demographic, clinical |
| *Outcome to be predicted:* | Mortality for inpatients |

**Step 2: Classify the type of prediction model evaluation**

| **Classify the evaluation based on its aim** | | | |
| --- | --- | --- | --- |
| **Type of prediction study** | **PROBAST boxes to complete** | **Tick as appropriate** | **Definition for type of prediction model study** |
| Development only | Development | ✔ | Prediction model development without external validation. These studies may include internal validation methods, such as bootstrapping and cross-validation techniques. |
| Development and validation | Development and validation | ✖ | Prediction model development combined with external validation in other participants in the same article. |
| Validation only | Validation | ✖ | External validation of existing (previously developed) model in other participants. |

|  | |
| --- | --- |
| **Publication reference** | Moulaei K, Ghasemian F, Bahaadinbeigy K, Ershad Sarbi R, Mohamadi Taghiabad Z. Predicting Mortality of COVID-19 Patients based on Data Mining Techniques. J Biomed Phys Eng. 2021 Oct 1;11(5):653-662. doi: 10.31661/jbpe.v0i0.2104-1300. PMID: 34722410; PMCID: PMC8546157. | |
| **Models of interest** | Decision tree (J48), MLP, KNN, random forest, and SVM |  |
| **Outcome of interest** | Mortality | |

**Step 3: Assess risk of bias and applicability**

| **DOMAIN 1:  Participants** | | | |
| --- | --- | --- | --- |
| **A. Risk of Bias** | | | |
| *Describe the sources of data and criteria for participant selection:*  DEV:  The dataset included demographic and clinical information of a total of 850 COVID-19 patients (250 deaths) from three hospitals in Iran from March 5 to September 22 of 2020. | | | |
|  | | Dev | Val |
| 1. Were appropriate data sources used, e.g. cohort, RCT or nested case-control study data? | | Y |  |
| 1. Were all inclusions and exclusions of participants appropriate? | | NI |  |
| **Risk of bias introduced by selection of participants** | **RISK:**  *(low/ high/ unclear)* | **unclear** |  |
| *Rationale of bias rating:* | | | |
| Inclusions and excursions of participants are not clear. | | | |
| **B. Applicability** | | | |
| *Describe included participants, setting and dates:*  It has been mentioned in Domain 1 – Section A. | | | |
| **Concern that the included participants and setting do not match the review question** | **CONCERN:**  *(low/ high/ unclear)* | **low** |  |
| *Rationale of applicability rating:* | | | |
| In our review, our source population is the general population. Therefore, this data is part of the general population. | | | |

| **DOMAIN 2:  Predictors** | | | |
| --- | --- | --- | --- |
| **A. Risk of Bias** | | | |
| *List and describe predictors included in the final model, e.g. definition and timing of assessment:*  16 factors were: Dyspnea, Underlying diseases, Headache, Weakness and lethargy, Body pain, Fatigue, Sore throat, Age, Dry cough, Diarrhea, Pain or pressure in the chest, High fever, Loss of sense of smell and taste, Nausea and Vomiting, Anorexia, Gender. The random forest performed the best based on AUC of 1.0, in predicting of mortality than other models | | | |
|  | | Dev | Val |
| 1. Were predictors defined and assessed in a similar way for all participants? | | PY |  |
| 1. Were predictor assessments made without knowledge of outcome data? | | PY |  |
| 1. Are all predictors available at the time the model is intended to be used? | | Y |  |
| **Risk of bias introduced by predictors or their assessment** | **RISK:**  *(low/ high/ unclear)* | **high** |  |
| *Rationale of bias rating:*  *The covariant assessment window was not defined to generate variables. The variable were captured in a limited timing for assessment on the time of admission* | | | |
| **B. Applicability** | | | |
| Concern that the definition, assessment or timing of predictors in the model do not match the review question | **CONCERN:**  *(low/ high/ unclear)* | **high** |  |
| *Rationale of applicability rating:*  When the covariate assessment window was not defined for predictors, it could cause limited reliability of the results and its applicability. we supposed the assessment should be done *on the time of admission which is a limited timing for assessment and may not be applicable in clinical setting to predict mortality.* | | | |

| **DOMAIN 3: Outcome** | | | |
| --- | --- | --- | --- |
| **A. Risk of Bias** | | | |
| *Describe the outcome, how it was defined and determined, and the time interval between predictor assessment and outcome determination:*  The primary outcome was in-hospital case-fatality rate. | | | |
|  | | Dev | Val |
| 1. Was the outcome determined appropriately? | | PY |  |
| 1. Was a pre-specified or standard outcome definition used? | | PY |  |
| 1. Were predictors excluded from the outcome definition? | | Y |  |
| 1. Was the outcome defined and determined in a similar way for all participants? | | PY |  |
| 1. Was the outcome determined without knowledge of predictor information? | | PY |  |
| 1. Was the time interval between predictor assessment and outcome determination appropriate? | | NI |  |
| **Risk of bias introduced by the outcome or its determination** | **RISK:**  *(low/ high/ unclear)* | **high** |  |
| *Rationale of bias rating:*  The time interval between predictors’ assessment and outcome was not defined. | | | |
| **B. Applicability** | | | |
| *At what time point was the outcome determined:*  *If a composite outcome was used, describe the relative frequency/distribution of each contributing outcome:* | | | |
| **Concern that the outcome, its definition, timing or determination do not match the review question** | **CONCERN:**  *(low/ high/ unclear)* | **high** |  |
| *Rationale of applicability rating:*  Having different time intervals between predictors assessment and outcome might cause different results in different settings. | | | |
| **DOMAIN 4: Analysis** | | | |
| **Risk of Bias** | | | |
| *Describe numbers of participants, number of candidate predictors, outcome events and events per candidate predictor:*  DEV:   training: 850 COVID-19 patients (250 deaths) | | | |
| *Describe how the model was developed (for example in regards to modelling technique (e.g. survival or logistic modelling), predictor selection, and risk group definition):*  decision tree (J48), multilayer perceptron, KNN, random forest, and SVM | | | |
| *Describe whether and how the model was validated, either internally (e.g. bootstrapping, cross validation, random split sample) or externally (e.g. temporal validation, geographical validation, different setting, different type of participants):*  Cross validation was used. | | | |
| *Describe the performance measures of the model, e.g. (re)calibration, discrimination, (re)classification, net benefit, and whether they were adjusted for optimism:*  The ROC analysis was performed. | | | |
| *Describe any participants who were excluded from the analysis:*  *Records that had missing values or had no resemblance to other data (Outlier) were deleted* | | | |
| *Describe missing data on predictors and outcomes as well as methods used for missing data:*  *NI* | | | |
|  | | Dev | Val |
| 1. Were there a reasonable number of participants with the outcome? | | PY |  |
| 1. Were continuous and categorical predictors handled appropriately? | | NI |  |
| 1. Were all enrolled participants included in the analysis? | | PY |  |
| 1. Were participants with missing data handled appropriately? | | NI |  |
| 1. Was selection of predictors based on univariable analysis avoided? | | NI |  |
| 1. Were complexities in the data (e.g. censoring, competing risks, sampling of controls) accounted for appropriately? | | PY |  |
| 1. Were relevant model performance measures evaluated appropriately? | | PN |  |
| 1. Were model overfitting and optimism in model performance accounted for? | | PY |  |
| 1. Do predictors and their assigned weights in the final model correspond to the results from multivariable analysis? | | PY |  |
| **Risk of bias introduced by the analysis** | **RISK:**  *(low/ high/ unclear)* | **high** |  |
| *Rationale of bias rating:*  The model was not evaluated with external validation. The analysis is based on input data only from three hospitals. Therefore the model may not be generalized to other settings. | | | |

**Step 4: Overall assessment**

| | **Reaching an overall judgement about risk of bias of the prediction model evaluation** | | | --- | --- | | **Low risk of bias** | If all domains were rated low risk of bias.  If a prediction model was developed without any external validation, and it was rated as low risk of bias for all domains, consider downgrading to **high risk of bias**. Such a model can only be considered as low risk of bias, if the development was based on a very large data set and included some form of internal validation. | | **High risk of bias** | If at least one domain is judged to be at **high risk of bias**. | | **Unclear risk of bias** | If an unclear risk of bias was noted in at least one domain and it was low risk for all other domains. |  | **Reaching an overall judgement about applicability of the prediction model evaluation** | | | --- | --- | | **Low concerns regarding applicability** | If low concerns regarding applicability for all domains, the prediction model evaluation is judged to have **low concerns regarding applicability**. | | **High concerns regarding applicability** | If high concerns regarding applicability for at least one domain, the prediction model evaluation is judged to have **high concerns regarding applicability**. | | **Unclear concerns regarding applicability** | If unclear concerns (but no “high concern”) regarding applicability for at least one domain, the prediction model evaluation is judged to have **unclear concerns regarding applicability** overall. | |
| --- | --- | --- | --- | --- | --- | --- | --- | --- | --- | --- | --- | --- | --- | --- | --- | --- |

| **Overall judgement about risk of bias and applicability of the prediction model evaluation** | | |
| --- | --- | --- |
| **Overall judgement of risk of bias** | **RISK:**  *(low/ high/ unclear)* | **high** |
| *Summary of sources of potential bias:*  When the covariate assessment window was not defined for predictors, it could cause limited reliability of the results and its applicability. we supposed the assessment should be done *on the time of admission which is a limited timing for assessment and may not be applicable in clinical settings to predict mortality.*  The time interval between predictors’ assessment and outcome was not defined. | | |
| **Overall judgement of applicability** | **CONCERN:**  *(low/ high/ unclear)* | **high** |
| *Summary of applicability concerns:*  When the covariate assessment window was not defined for predictors, it could cause limited reliability of the results and its applicability  Having different time intervals between predictors assessment and outcome might cause different results in different settings.  The model was not evaluated with external validation. The analysis is based on input data only from three hospitals. Therefore the model may not be generalized to other settings. | | |

**Appendix 24**. Migriño 2021 - Bias analysis using PROBAST.

**Step 1: Specify your systematic review question**

| **Criteria** | **Specify your systematic review question** |
| --- | --- |
| *Intended use of model:* | To predict the outcomes of COVID-19 cases from data publicly available |
| ***Participants*** *including selection criteria and setting:* | positive tested COVID-19 patients |
| ***Predictors*** *(used in prediction modelling), including types of predictors (e.g. history, clinical examination, biochemical markers, imaging tests), time of measurement, specific measurement issues (e.g., any requirements/ prohibitions for specialized equipment):* | Demographic and history of hospital admission |
| *Outcome to be predicted:* | Mortality |

**Step 2: Classify the type of prediction model evaluation**

| **Classify the evaluation based on its aim** | | | |
| --- | --- | --- | --- |
| **Type of prediction study** | **PROBAST boxes to complete** | **Tick as appropriate** | **Definition for type of prediction model study** |
| Development only | Development | ✔ | Prediction model development without external validation. These studies may include internal validation methods, such as bootstrapping and cross-validation techniques. |
| Development and validation | Development and validation | ✖ | Prediction model development combined with external validation in other participants in the same article. |
| Validation only | Validation | ✖ | External validation of existing (previously developed) model in other participants. |

|  | |
| --- | --- |
| **Publication reference** | Migriño JR Jr, Batangan ARU. Using machine learning to create a decision tree model to predict outcomes of COVID-19 cases in the Philippines. Western Pac Surveill Response J. 2021 Sep 14;12(3):56-64. doi: 10.5365/wpsar.2021.12.3.831. PMID: 34703636; PMCID: PMC8521127. | |
| **Models of interest** | Decision tree, naïve Bayes |  |
| **Outcome of interest** | Mortality | |

**Step 3: Assess risk of bias and applicability**

| **DOMAIN 1:  Participants** | | | |
| --- | --- | --- | --- |
| **A. Risk of Bias** | | | |
| *Describe the sources of data and criteria for participant selection:*  DEV:  The publicly available DOH COVID Data Drop database for 25 August 2020 was used to extract demographic and history of hospital admission information of  132 939 COVID-19  patients (2.3% died). | | | |
|  | | Dev | Val |
| 1. Were appropriate data sources used, e.g. cohort, RCT or nested case-control study data? | | N |  |
| 1. Were all inclusions and exclusions of participants appropriate? | | NI |  |
| **Risk of bias introduced by selection of participants** | **RISK:**  *(low/ high/ unclear)* | **high** |  |
| *Rationale of bias rating:* | | | |
| Inclusions and excursions of participants are not clear. Using only demographic and history of hospital admission is not enough data for the mortality prediction. | | | |
| **B. Applicability** | | | |
| *Describe included participants, setting and dates:*  It has been mentioned in Domain 1 – Section A. | | | |
| **Concern that the included participants and setting do not match the review question** | **CONCERN:**  *(low/ high/ unclear)* | **high** |  |
| *Rationale of applicability rating:* | | | |
|  | | | |

| **DOMAIN 2:  Predictors** | | | |
| --- | --- | --- | --- |
| **A. Risk of Bias** | | | |
| *List and describe predictors included in the final model, e.g. definition and timing of assessment:*  Age and history of hospital admission | | | |
|  | | Dev | Val |
| 1. Were predictors defined and assessed in a similar way for all participants? | | PY |  |
| 1. Were predictor assessments made without knowledge of outcome data? | | PY |  |
| 1. Are all predictors available at the time the model is intended to be used? | | Y |  |
| **Risk of bias introduced by predictors or their assessment** | **RISK:**  *(low/ high/ unclear)* | **high** |  |
| *Rationale of bias rating:*  *The covariant assessment window was not defined to generate variables.* | | | |
| **B. Applicability** | | | |
| Concern that the definition, assessment or timing of predictors in the model do not match the review question | **CONCERN:**  *(low/ high/ unclear)* | **high** |  |
| *Rationale of applicability rating:*  Age alone is not a good enough predictor. Patients might die because they are old not because of Covid-19. | | | |

| **DOMAIN 3: Outcome** | | | |
| --- | --- | --- | --- |
| **A. Risk of Bias** | | | |
| *Describe the outcome, how it was defined and determined, and the time interval between predictor assessment and outcome determination:*  Death. | | | |
|  | | Dev | Val |
| 1. Was the outcome determined appropriately? | | NI |  |
| 1. Was a pre-specified or standard outcome definition used? | | N |  |
| 1. Were predictors excluded from the outcome definition? | | Y |  |
| 1. Was the outcome defined and determined in a similar way for all participants? | | PY |  |
| 1. Was the outcome determined without knowledge of predictor information? | | PY |  |
| 1. Was the time interval between predictor assessment and outcome determination appropriate? | | NI |  |
| **Risk of bias introduced by the outcome or its determination** | **RISK:**  *(low/ high/ unclear)* | **high** |  |
| *Rationale of bias rating:*  The time interval between predictors’ assessment and outcome was not defined.  The death as an outcome was not defined whether patients died due to Covid or for some other reasons. | | | |
| **B. Applicability** | | | |
| *At what time point was the outcome determined:*  *If a composite outcome was used, describe the relative frequency/distribution of each contributing outcome:* | | | |
| **Concern that the outcome, its definition, timing or determination do not match the review question** | **CONCERN:**  *(low/ high/ unclear)* | **high** |  |
| *Rationale of applicability rating:* | | | |
| **DOMAIN 4: Analysis** | | | |
| **Risk of Bias** | | | |
| *Describe numbers of participants, number of candidate predictors, outcome events and events per candidate predictor:*  DEV:   training:  132 939 COVID-19  patients (2.3% died) | | | |
| *Describe how the model was developed (for example in regards to modelling technique (e.g. survival or logistic modelling), predictor selection, and risk group definition):*  decision tree, naïve Bayes | | | |
| *Describe whether and how the model was validated, either internally (e.g. bootstrapping, cross validation, random split sample) or externally (e.g. temporal validation, geographical validation, different setting, different type of participants):*  Cross validation was used. | | | |
| *Describe the performance measures of the model, e.g. (re)calibration, discrimination, (re)classification, net benefit, and whether they were adjusted for optimism:*  The ROC analysis was performed. | | | |
| *Describe any participants who were excluded from the analysis:*  *NI* | | | |
| *Describe missing data on predictors and outcomes as well as methods used for missing data:*  *NI* | | | |
|  | | Dev | Val |
| 1. Were there a reasonable number of participants with the outcome? | | PY |  |
| 1. Were continuous and categorical predictors handled appropriately? | | NI |  |
| 1. Were all enrolled participants included in the analysis? | | PY |  |
| 1. Were participants with missing data handled appropriately? | | NI |  |
| 1. Was selection of predictors based on univariable analysis avoided? | | NI |  |
| 1. Were complexities in the data (e.g. censoring, competing risks, sampling of controls) accounted for appropriately? | | PY |  |
| 1. Were relevant model performance measures evaluated appropriately? | | PN |  |
| 1. Were model overfitting and optimism in model performance accounted for? | | PY |  |
| 1. Do predictors and their assigned weights in the final model correspond to the results from multivariable analysis? | | PY |  |
| **Risk of bias introduced by the analysis** | **RISK:**  *(low/ high/ unclear)* | **high** |  |
| *Rationale of bias rating:*  The model was not evaluated with external validation. The analysis is based on input data only from the Philippines. Therefore the model may not be generalized to other settings. | | | |

**Step 4: Overall assessment**

| | **Reaching an overall judgement about risk of bias of the prediction model evaluation** | | | --- | --- | | **Low risk of bias** | If all domains were rated low risk of bias.  If a prediction model was developed without any external validation, and it was rated as low risk of bias for all domains, consider downgrading to **high risk of bias**. Such a model can only be considered as low risk of bias, if the development was based on a very large data set and included some form of internal validation. | | **High risk of bias** | If at least one domain is judged to be at **high risk of bias**. | | **Unclear risk of bias** | If an unclear risk of bias was noted in at least one domain and it was low risk for all other domains. |  | **Reaching an overall judgement about applicability of the prediction model evaluation** | | | --- | --- | | **Low concerns regarding applicability** | If low concerns regarding applicability for all domains, the prediction model evaluation is judged to have **low concerns regarding applicability**. | | **High concerns regarding applicability** | If high concerns regarding applicability for at least one domain, the prediction model evaluation is judged to have **high concerns regarding applicability**. | | **Unclear concerns regarding applicability** | If unclear concerns (but no “high concern”) regarding applicability for at least one domain, the prediction model evaluation is judged to have **unclear concerns regarding applicability** overall. | |
| --- | --- | --- | --- | --- | --- | --- | --- | --- | --- | --- | --- | --- | --- | --- | --- | --- |

| **Overall judgement about risk of bias and applicability of the prediction model evaluation** | | |
| --- | --- | --- |
| **Overall judgement of risk of bias** | **RISK:**  *(low/ high/ unclear)* | **high** |
| *Summary of sources of potential bias:*  Age alone is not a good enough predictor. Patients might die because they are old not because of Covid-19.  The time interval between predictors’ assessment and outcome was not defined.  The death as an outcome was not defined whether patients died due to Covid or for some other reasons. | | |
| **Overall judgement of applicability** | **CONCERN:**  *(low/ high/ unclear)* | **high** |
| *Summary of applicability concerns:*  The model was not evaluated with external validation. The analysis is based on input data only from the Philippines. Therefore the model may not be generalized to other settings. | | |

**Appendix 25**. Banoei 2021 - Bias analysis using PROBAST.

**Step 1: Specify your systematic review question**

| **Criteria** | **Specify your systematic review question** |
| --- | --- |
| *Intended use of model:* | To develop a mortality prediction model for hospitalized COVID-19 patients |
| ***Participants*** *including selection criteria and setting:* | Positive tested COVID-19 patients and hospitalized |
| ***Predictors*** *(used in prediction modelling), including types of predictors (e.g. history, clinical examination, biochemical markers, imaging tests), time of measurement, specific measurement issues (e.g., any requirements/ prohibitions for specialized equipment):* | Demographic, clinical data including comorbidities, patients’ vitals, anthropometric measurements, chronic treatments and laboratory including blood markers |
| *Outcome to be predicted:* | Mortality |

**Step 2: Classify the type of prediction model evaluation**

| **Classify the evaluation based on its aim** | | | |
| --- | --- | --- | --- |
| **Type of prediction study** | **PROBAST boxes to complete** | **Tick as appropriate** | **Definition for type of prediction model study** |
| Development only | Development | ✔ | Prediction model development without external validation. These studies may include internal validation methods, such as bootstrapping and cross-validation techniques. |
| Development and validation | Development and validation | ✖ | Prediction model development combined with external validation in other participants in the same article. |
| Validation only | Validation | ✖ | External validation of existing (previously developed) model in other participants. |

|  | |
| --- | --- |
| **Publication reference** | Banoei MM, Dinparastisaleh R, Zadeh AV, Mirsaeidi M. Machine-learning-based COVID-19 mortality prediction model and identification of patients at low and high risk of dying. Crit Care. 2021 Sep 8;25(1):328. doi: 10.1186/s13054-021-03749-5. PMID: 34496940; PMCID: PMC8424411. | |
| **Models of interest** | Inspired modification of partial least square (SIMPLS)-based model |  |
| **Outcome of interest** | Mortality | |

**Step 3: Assess risk of bias and applicability**

| **DOMAIN 1:  Participants** | | | |
| --- | --- | --- | --- |
| **A. Risk of Bias** | | | |
| *Describe the sources of data and criteria for participant selection:*  DEV:  Data were collected from 400 Covid-19 patients (31 *deaths*)admitted at the University of Miami Hospital, Miller School of Medicine, Miami, FL, USA, since June 2020.  108 out of 250 features including demographic, clinical data including comorbidities, patients’ vitals, anthropometric measurements, chronic treatments and laboratory including blood markers, were collected during the admission. | | | |
|  | | Dev | Val |
| 1. Were appropriate data sources used, e.g. cohort, RCT or nested case-control study data? | | Y |  |
| 1. Were all inclusions and exclusions of participants appropriate? | | NI |  |
| **Risk of bias introduced by selection of participants** | **RISK:**  *(low/ high/ unclear)* | **unclear** |  |
| *Rationale of bias rating:* | | | |
| Inclusions and excursions of participants are not clear. | | | |
| **B. Applicability** | | | |
| *Describe included participants, setting and dates:*  It has been mentioned in Domain 1 – Section A. | | | |
| **Concern that the included participants and setting do not match the review question** | **CONCERN:**  *(low/ high/ unclear)* | **low** |  |
| *Rationale of applicability rating:* | | | |
| In our review, our source population is the general population. Therefore, this data is part of the general population. | | | |

| **DOMAIN 2:  Predictors** | | | |
| --- | --- | --- | --- |
| **A. Risk of Bias** | | | |
| *List and describe predictors included in the final model, e.g. definition and timing of assessment:*  Age, platelet count, SpO2, blood urea nitrogen (BUN), hemoglobin, C-reactive protein, neutrophil count, and sodium. | | | |
|  | | Dev | Val |
| 1. Were predictors defined and assessed in a similar way for all participants? | | PY |  |
| 1. Were predictor assessments made without knowledge of outcome data? | | PY |  |
| 1. Are all predictors available at the time the model is intended to be used? | | Y |  |
| **Risk of bias introduced by predictors or their assessment** | **RISK:**  *(low/ high/ unclear)* | **high** |  |
| *Rationale of bias rating:*  *The covariant assessment window was not defined to generate variables. For example, for* comorbidities*, even if we assume it was* between the admission time to the outcome, it might not be suitable. | | | |
| **B. Applicability** | | | |
| Concern that the definition, assessment or timing of predictors in the model do not match the review question | **CONCERN:**  *(low/ high/ unclear)* | **high** |  |
| *Rationale of applicability rating:*  When the covariate assessment window was not defined for predictors, it could cause limited reliability of the results and its applicability | | | |

| **DOMAIN 3: Outcome** | | | |
| --- | --- | --- | --- |
| **A. Risk of Bias** | | | |
| *Describe the outcome, how it was defined and determined, and the time interval between predictor assessment and outcome determination:*  The primary outcome was in-hospital case-fatality rate. | | | |
|  | | Dev | Val |
| 1. Was the outcome determined appropriately? | | PY |  |
| 1. Was a pre-specified or standard outcome definition used? | | PY |  |
| 1. Were predictors excluded from the outcome definition? | | Y |  |
| 1. Was the outcome defined and determined in a similar way for all participants? | | PY |  |
| 1. Was the outcome determined without knowledge of predictor information? | | PY |  |
| 1. Was the time interval between predictor assessment and outcome determination appropriate? | | NI |  |
| **Risk of bias introduced by the outcome or its determination** | **RISK:**  *(low/ high/ unclear)* | **high** |  |
| *Rationale of bias rating:*  The time interval between predictors’ assessment and outcome was not defined. | | | |
| **B. Applicability** | | | |
| *At what time point was the outcome determined:*  *If a composite outcome was used, describe the relative frequency/distribution of each contributing outcome:* | | | |
| **Concern that the outcome, its definition, timing or determination do not match the review question** | **CONCERN:**  *(low/ high/ unclear)* | **high** |  |
| *Rationale of applicability rating:*  Having different time intervals between predictors’ assessment and outcome might cause different results in different settings. The data of death patients outside of hospitals are not evaluated in the model which reduces its generality. | | | |
| **DOMAIN 4: Analysis** | | | |
| **Risk of Bias** | | | |
| *Describe numbers of participants, number of candidate predictors, outcome events and events per candidate predictor:*  DEV:   400 Covid-19 patients (31 *deaths*) | | | |
| *Describe how the model was developed (for example in regards to modelling technique (e.g. survival or logistic modelling), predictor selection, and risk group definition):*  Inspired modification of partial least square (SIMPLS)-based model | | | |
| *Describe whether and how the model was validated, either internally (e.g. bootstrapping, cross validation, random split sample) or externally (e.g. temporal validation, geographical validation, different setting, different type of participants):*  Cross validation was not used. | | | |
| *Describe the performance measures of the model, e.g. (re)calibration, discrimination, (re)classification, net benefit, and whether they were adjusted for optimism:*  The ROC analysis was performed. | | | |
| *Describe any participants who were excluded from the analysis:*  *NI* | | | |
| *Describe missing data on predictors and outcomes as well as methods used for missing data:*  The maximum level of missing values was 7% among the variables. Using imputation methods, new data were created by replacing all missing values with the estimated values using mean imputation. | | | |
|  | | Dev | Val |
| 1. Were there a reasonable number of participants with the outcome? | | PY |  |
| 1. Were continuous and categorical predictors handled appropriately? | | NI |  |
| 1. Were all enrolled participants included in the analysis? | | PY |  |
| 1. Were participants with missing data handled appropriately? | | NI |  |
| 1. Was selection of predictors based on univariable analysis avoided? | | NI |  |
| 1. Were complexities in the data (e.g. censoring, competing risks, sampling of controls) accounted for appropriately? | | PY |  |
| 1. Were relevant model performance measures evaluated appropriately? | | PN |  |
| 1. Were model overfitting and optimism in model performance accounted for? | | PY |  |
| 1. Do predictors and their assigned weights in the final model correspond to the results from multivariable analysis? | | PY |  |
| **Risk of bias introduced by the analysis** | **RISK:**  *(low/ high/ unclear)* | **high** |  |
| *Rationale of bias rating:*  The model was not evaluated with external validation. The analysis is based on input data from only one hospital. Therefore the model may not be generalized to other settings.  Cross validation was not used and deaths size was so small (*n* = 31) therfore the results was dealingare with overfitting. | | | |

**Step 4: Overall assessment**

| | **Reaching an overall judgement about risk of bias of the prediction model evaluation** | | | --- | --- | | **Low risk of bias** | If all domains were rated low risk of bias.  If a prediction model was developed without any external validation, and it was rated as low risk of bias for all domains, consider downgrading to **high risk of bias**. Such a model can only be considered as low risk of bias, if the development was based on a very large data set and included some form of internal validation. | | **High risk of bias** | If at least one domain is judged to be at **high risk of bias**. | | **Unclear risk of bias** | If an unclear risk of bias was noted in at least one domain and it was low risk for all other domains. |  | **Reaching an overall judgement about applicability of the prediction model evaluation** | | | --- | --- | | **Low concerns regarding applicability** | If low concerns regarding applicability for all domains, the prediction model evaluation is judged to have **low concerns regarding applicability**. | | **High concerns regarding applicability** | If high concerns regarding applicability for at least one domain, the prediction model evaluation is judged to have **high concerns regarding applicability**. | | **Unclear concerns regarding applicability** | If unclear concerns (but no “high concern”) regarding applicability for at least one domain, the prediction model evaluation is judged to have **unclear concerns regarding applicability** overall. | |
| --- | --- | --- | --- | --- | --- | --- | --- | --- | --- | --- | --- | --- | --- | --- | --- | --- |

| **Overall judgement about risk of bias and applicability of the prediction model evaluation** | | |
| --- | --- | --- |
| **Overall judgement of risk of bias** | **RISK:**  *(low/ high/ unclear)* | **high** |
| *Summary of sources of potential bias:*  Having different time intervals between predictors’ assessment and outcome might cause different results in different settings. The data of death patients outside of hospitals are not evaluated in the model which reduces its generality.  *The covariant assessment window was not defined to generate variables. For example, for* comorbidities*, even if we assume it was* between November 6, 2020, to February 5, it might not be suitable to check all chronic diseases during the life of a patients.  The time interval between predictors assessment and outcome was not defined. | | |
| **Overall judgement of applicability** | **CONCERN:**  *(low/ high/ unclear)* | **high** |
| *Summary of applicability concerns:*  When the covariate assessment window was not defined for predictors, it could cause limited reliability of the results and its applicability  Having different time intervals between predictors assessment and outcome might cause different results in different settings.  The model was not evaluated with external validation. The analysis is based on input data from only one hospital. Therefore the model may not be generalized to other settings.  Cross validation was not used and even deaths size was so small (*n* = 31) thefore results was dealingare with overfitting. | | |

**Appendix 26**. Dabbah 2021 - Bias analysis using PROBAST.

**Step 1: Specify your systematic review question**

| **Criteria** | **Specify your systematic review question** |
| --- | --- |
| *Intended use of model:* | To develop a mortality prediction model for hospitalized COVID-19 patients |
| ***Participants*** *including selection criteria and setting:* | Positive tested COVID-19 patients and hospitalized |
| ***Predictors*** *(used in prediction modelling), including types of predictors (e.g. history, clinical examination, biochemical markers, imaging tests), time of measurement, specific measurement issues (e.g., any requirements/ prohibitions for specialized equipment):* | Demographic, clinical data including comorbidities, patients’ vitals, anthropometric measurements, chronic treatments and laboratory including blood markers |
| *Outcome to be predicted:* | Mortality |

**Step 2: Classify the type of prediction model evaluation**

| **Classify the evaluation based on its aim** | | | |
| --- | --- | --- | --- |
| **Type of prediction study** | **PROBAST boxes to complete** | **Tick as appropriate** | **Definition for type of prediction model study** |
| Development only | Development | ✔ | Prediction model development without external validation. These studies may include internal validation methods, such as bootstrapping and cross-validation techniques. |
| Development and validation | Development and validation | ✖ | Prediction model development combined with external validation in other participants in the same article. |
| Validation only | Validation | ✖ | External validation of existing (previously developed) model in other participants. |

|  | |
| --- | --- |
| **Publication reference** | Dabbah MA, Reed AB, Booth ATC, Yassaee A, Despotovic A, Klasmer B, Binning E, Aral M, Plans D, Morelli D, Labrique AB, Mohan D. Machine learning approach to dynamic risk modeling of mortality in COVID-19: a UK Biobank study. Sci Rep. 2021 Aug 19;11(1):16936. doi: 10.1038/s41598-021-95136-x. PMID: 34413324; PMCID: PMC8376891. | |
| **Models of interest** | Randome Forest |  |
| **Outcome of interest** | Mortality | |

**Step 3: Assess risk of bias and applicability**

| **DOMAIN 1:  Participants** | | | |
| --- | --- | --- | --- |
| **A. Risk of Bias** | | | |
| *Describe the sources of data and criteria for participant selection:*  DEV:  There were 11,245 COVID-19 patients (aged 51–85 years, mean: 66.9) including 640 (5.7%, mean age:76) deaths in the UK Biobank (UKB) .  Data set included patient characteristics, pre-existing conditions, symptoms, and vital signs | | | |
|  | | Dev | Val |
| 1. Were appropriate data sources used, e.g. cohort, RCT or nested case-control study data? | | Y |  |
| 1. Were all inclusions and exclusions of participants appropriate? | | PY |  |
| **Risk of bias introduced by selection of participants** | **RISK:**  *(low/ high/ unclear)* | **low** |  |
| *Rationale of bias rating: No concerns* | | | |
| **B. Applicability** | | | |
| *Describe included participants, setting and dates:*  It has been mentioned in Domain 1 – Section A. | | | |
| **Concern that the included participants and setting do not match the review question** | **CONCERN:**  *(low/ high/ unclear)* | **low** |  |
| *Rationale of applicability rating:* | | | |
| In our review, our source population is the general population. Therefore, this data is part of the general population. | | | |

| **DOMAIN 2:  Predictors** | | | |
| --- | --- | --- | --- |
| **A. Risk of Bias** | | | |
| *List and describe predictors included in the final model, e.g. definition and timing of assessment:*  64 out of 12,000 features selected as: 3 vital signs; 12 symptoms; 32 pre-existing clinical conditions; 5 medications and treatments; and 13 patient characteristics.[​](https://www.ncbi.nlm.nih.gov/pmc/articles/PMC8376891/table/Tab1/)  Pre-existing medical conditions included only when reported more than one week prior to COVID-19 positive test result. Symptoms and vitals included only from primary care (GP) records when reported within + /- two weeks of COVID-19 positive test. | | | |
|  | | Dev | Val |
| 1. Were predictors defined and assessed in a similar way for all participants? | | PY |  |
| 1. Were predictor assessments made without knowledge of outcome data? | | PY |  |
| 1. Are all predictors available at the time the model is intended to be used? | | Y |  |
| **Risk of bias introduced by predictors or their assessment** | **RISK:**  *(low/ high/ unclear)* | **unclear** |  |
| *Rationale of bias rating:*  *Different covariant assessment windows were defined for different data types to generate variables. For example,* Pre-existing medical conditions included only when reported more than one week prior to COVID-19 positive test result. Symptoms and vitals included only from primary care (GP) records when reported within + /- two weeks of COVID-19 positive test. However these definitions were without references. | | | |
| **B. Applicability** | | | |
| Concern that the definition, assessment or timing of predictors in the model do not match the review question | **CONCERN:**  *(low/ high/ unclear)* | **unclear** |  |
| *Rationale of applicability rating:* | | | |

| **DOMAIN 3: Outcome** | | | |
| --- | --- | --- | --- |
| **A. Risk of Bias** | | | |
| *Describe the outcome, how it was defined and determined, and the time interval between predictor assessment and outcome determination:*  The primary outcome was in-hospital case-fatality rate. | | | |
|  | | Dev | Val |
| 1. Was the outcome determined appropriately? | | PY |  |
| 1. Was a pre-specified or standard outcome definition used? | | PY |  |
| 1. Were predictors excluded from the outcome definition? | | Y |  |
| 1. Was the outcome defined and determined in a similar way for all participants? | | PY |  |
| 1. Was the outcome determined without knowledge of predictor information? | | PY |  |
| 1. Was the time interval between predictor assessment and outcome determination appropriate? | | NI |  |
| **Risk of bias introduced by the outcome or its determination** | **RISK:**  *(low/ high/ unclear)* | **unclear** |  |
| *Rationale of bias rating:*  The time interval between predictors’ assessment and outcome was defined without reference. | | | |
| **B. Applicability** | | | |
| *At what time point was the outcome determined:*  *If a composite outcome was used, describe the relative frequency/distribution of each contributing outcome:* | | | |
| **Concern that the outcome, its definition, timing or determination do not match the review question** | **CONCERN:**  *(low/ high/ unclear)* | **high** |  |
| *Rationale of applicability rating:*  The data of death patients outside of hospitals were not evaluated in the model which reduces its generality. The mean age of COVID-19 patients was 66.9 which was high. It happened because patients without hospital data were excluded. Consequently it could cause bias in prediction model since age was considered an important predictor. | | | |
| **DOMAIN 4: Analysis** | | | |
| **Risk of Bias** | | | |
| *Describe numbers of participants, number of candidate predictors, outcome events and events per candidate predictor:*  DEV:   400 Covid-19 patients (31 *deaths*) | | | |
| *Describe how the model was developed (for example in regards to modelling technique (e.g. survival or logistic modelling), predictor selection, and risk group definition):*  Random forest | | | |
| *Describe whether and how the model was validated, either internally (e.g. bootstrapping, cross validation, random split sample) or externally (e.g. temporal validation, geographical validation, different setting, different type of participants):*  Cross validation was used. | | | |
| *Describe the performance measures of the model, e.g. (re)calibration, discrimination, (re)classification, net benefit, and whether they were adjusted for optimism:*  The ROC analysis was performed. | | | |
| *Describe any participants who were excluded from the analysis:*  *patients without hospital records data* | | | |
| *Describe missing data on predictors and outcomes as well as methods used for missing data:*  missing value substitution (replacing missing values or records with the mean value of the UK Biobank database); | | | |
|  | | Dev | Val |
| 1. Were there a reasonable number of participants with the outcome? | | PY |  |
| 1. Were continuous and categorical predictors handled appropriately? | | NI |  |
| 1. Were all enrolled participants included in the analysis? | | PY |  |
| 1. Were participants with missing data handled appropriately? | | NI |  |
| 1. Was selection of predictors based on univariable analysis avoided? | | NI |  |
| 1. Were complexities in the data (e.g. censoring, competing risks, sampling of controls) accounted for appropriately? | | PY |  |
| 1. Were relevant model performance measures evaluated appropriately? | | PN |  |
| 1. Were model overfitting and optimism in model performance accounted for? | | PY |  |
| 1. Do predictors and their assigned weights in the final model correspond to the results from multivariable analysis? | | PY |  |
| **Risk of bias introduced by the analysis** | **RISK:**  *(low/ high/ unclear)* | **high** |  |
| *Rationale of bias rating:*  Analysis was performed only using a random forest model. It might be useful to try linear models such as logistic regression which is more familiar with clinician and compare the results.  The model was not evaluated with external validation. The analysis is based on input data from UKB which may impact the generalizability of the model. | | | |

**Step 4: Overall assessment**

| | **Reaching an overall judgement about risk of bias of the prediction model evaluation** | | | --- | --- | | **Low risk of bias** | If all domains were rated low risk of bias.  If a prediction model was developed without any external validation, and it was rated as low risk of bias for all domains, consider downgrading to **high risk of bias**. Such a model can only be considered as low risk of bias, if the development was based on a very large data set and included some form of internal validation. | | **High risk of bias** | If at least one domain is judged to be at **high risk of bias**. | | **Unclear risk of bias** | If an unclear risk of bias was noted in at least one domain and it was low risk for all other domains. |  | **Reaching an overall judgement about applicability of the prediction model evaluation** | | | --- | --- | | **Low concerns regarding applicability** | If low concerns regarding applicability for all domains, the prediction model evaluation is judged to have **low concerns regarding applicability**. | | **High concerns regarding applicability** | If high concerns regarding applicability for at least one domain, the prediction model evaluation is judged to have **high concerns regarding applicability**. | | **Unclear concerns regarding applicability** | If unclear concerns (but no “high concern”) regarding applicability for at least one domain, the prediction model evaluation is judged to have **unclear concerns regarding applicability** overall. | |
| --- | --- | --- | --- | --- | --- | --- | --- | --- | --- | --- | --- | --- | --- | --- | --- | --- |

| **Overall judgement about risk of bias and applicability of the prediction model evaluation** | | |
| --- | --- | --- |
| **Overall judgement of risk of bias** | **RISK:**  *(low/ high/ unclear)* | **high** |
| *Summary of sources of potential bias:*  *Different covariant assessment windows were defined for different data types to generate variables. For example,* Pre-existing medical conditions included only when reported more than one week prior to COVID-19 positive test result. Symptoms and vitals included only from primary care (GP) records when reported within + /- two weeks of COVID-19 positive test. However these definitions were without references.  The time interval between predictors’ assessment and outcome was defined without reference.  Analysis was performed only using a random forest model. It might be useful to try linear models such as logistic regression which is more familiar with clinician and compare the results. | | |
| **Overall judgement of applicability** | **CONCERN:**  *(low/ high/ unclear)* | **high** |
| *Summary of applicability concerns:*  The data of death patients outside of hospitals were not evaluated in the model which reduces its generality. The mean age of COVID-19 patients was 66.9 which was high. It happened because patients without hospital data were excluded. Consequently it could cause bias in the prediction model since age was considered an important predictor.  The model was not evaluated with external validation. The analysis is based on input data from UKB which may impact the generalizability of the model. | | |

**Appendix 27**. De Souza 2021 - Bias analysis using PROBAST.

**Step 1: Specify your systematic review question**

| **Criteria** | **Specify your systematic review question** |
| --- | --- |
| *Intended use of model:* | To predict the disease outcome for positive COVID-19 patients |
| ***Participants*** *including selection criteria and setting:* | positive tested COVID-19 patients |
| ***Predictors*** *(used in prediction modelling), including types of predictors (e.g. history, clinical examination, biochemical markers, imaging tests), time of measurement, specific measurement issues (e.g., any requirements/ prohibitions for specialized equipment):* | Characteristics of patients such as age, comorbidities and varied clinical symptoms and and a recent traveling history |
| *Outcome to be predicted:* | Mortality |

**Step 2: Classify the type of prediction model evaluation**

| **Classify the evaluation based on its aim** | | | |
| --- | --- | --- | --- |
| **Type of prediction study** | **PROBAST boxes to complete** | **Tick as appropriate** | **Definition for type of prediction model study** |
| Development only | Development | ✔ | Prediction model development without external validation. These studies may include internal validation methods, such as bootstrapping and cross-validation techniques. |
| Development and validation | Development and validation | ✖ | Prediction model development combined with external validation in other participants in the same article. |
| Validation only | Validation | ✖ | External validation of existing (previously developed) model in other participants. |

|  | |
| --- | --- |
| **Publication reference** | De Souza FSH, Hojo-Souza NS, Dos Santos EB, Da Silva CM, Guidoni DL. Predicting the Disease Outcome in COVID-19 Positive Patients Through Machine Learning: A Retrospective Cohort Study With Brazilian Data. Front Artif Intell. 2021 Aug 13;4:579931. doi: 10.3389/frai.2021.579931. PMID: 34514377; PMCID: PMC8427867. | |
| **Models of interest** | XGBOOST |  |
| **Outcome of interest** | Mortality | |

**Step 3: Assess risk of bias and applicability**

| **DOMAIN 1:  Participants** | | | |
| --- | --- | --- | --- |
| **A. Risk of Bias** | | | |
| *Describe the sources of data and criteria for participant selection:*  DEV:  The database used is publicly available on the Espírito Santo state portal ([Government of the state o, 2020](https://www.frontiersin.org/articles/10.3389/frai.2021.579931/full#B13)) included characteristics of patients such as age, comorbidities and varied clinical symptoms and a recent traveling history of 13, 690 COVID-19 patients. | | | |
|  | | Dev | Val |
| 1. Were appropriate data sources used, e.g. cohort, RCT or nested case-control study data? | | Y |  |
| 1. Were all inclusions and exclusions of participants appropriate? | | PY |  |
| **Risk of bias introduced by selection of participants** | **RISK:**  *(low/ high/ unclear)* | **low** |  |
| *Rationale of bias rating: No Concerns* | | | |
| **B. Applicability** | | | |
| *Describe included participants, setting and dates:*  It has been mentioned in Domain 1 – Section A. | | | |
| **Concern that the included participants and setting do not match the review question** | **CONCERN:**  *(low/ high/ unclear)* | **low** |  |
| *Rationale of applicability rating:* | | | |
| In our review, our source population is the general population. Therefore, this data is part of the general population. | | | |

| **DOMAIN 2:  Predictors** | | | |
| --- | --- | --- | --- |
| **A. Risk of Bias** | | | |
| *List and describe predictors included in the final model, e.g. definition and timing of assessment:*  Age, respiratory distress and comorbidities such as kidney disease, diabetes, cardiac disease and obesity, smoking, and fever symptoms | | | |
|  | | Dev | Val |
| 1. Were predictors defined and assessed in a similar way for all participants? | | NI |  |
| 1. Were predictor assessments made without knowledge of outcome data? | | PY |  |
| 1. Are all predictors available at the time the model is intended to be used? | | Y |  |
| **Risk of bias introduced by predictors or their assessment** | **RISK:**  *(low/ high/ unclear)* | **high** |  |
| *Rationale of bias rating:*  *Different covariant assessment windows were not clearly defined for different data types to generate variables.* | | | |
| **B. Applicability** | | | |
| Concern that the definition, assessment or timing of predictors in the model do not match the review question | **CONCERN:**  *(low/ high/ unclear)* | **high** |  |
| *Rationale of applicability rating: see above.* | | | |

| **DOMAIN 3: Outcome** | | | |
| --- | --- | --- | --- |
| **A. Risk of Bias** | | | |
| *Describe the outcome, how it was defined and determined, and the time interval between predictor assessment and outcome determination:*  The outcome was defined as death or cure due to hospitalization. | | | |
|  | | Dev | Val |
| 1. Was the outcome determined appropriately? | | PY |  |
| 1. Was a pre-specified or standard outcome definition used? | | PY |  |
| 1. Were predictors excluded from the outcome definition? | | Y |  |
| 1. Was the outcome defined and determined in a similar way for all participants? | | PY |  |
| 1. Was the outcome determined without knowledge of predictor information? | | PY |  |
| 1. Was the time interval between predictor assessment and outcome determination appropriate? | | NI |  |
| **Risk of bias introduced by the outcome or its determination** | **RISK:**  *(low/ high/ unclear)* | **unclear** |  |
| *Rationale of bias rating:*  The time interval between predictors’ assessment and outcome was not defined. | | | |
| **B. Applicability** | | | |
| *At what time point was the outcome determined:*  *If a composite outcome was used, describe the relative frequency/distribution of each contributing outcome:* | | | |
| **Concern that the outcome, its definition, timing or determination do not match the review question** | **CONCERN:**  *(low/ high/ unclear)* | **high** |  |
| *Rationale of applicability rating:*  The data of death patients outside of hospitals were not evaluated in the model which reduces its generality. | | | |
| **DOMAIN 4: Analysis** | | | |
| **Risk of Bias** | | | |
| *Describe numbers of participants, number of candidate predictors, outcome events and events per candidate predictor:*  DEV:   The training cohort and the validation cohort included 4, 826 (91.79*%* cured and 8.21*%* deceased) and 3, 617( 93.89*%* cured and 6.11*%* died) patients respectively. | | | |
| *Describe how the model was developed (for example in regards to modelling technique (e.g. survival or logistic modelling), predictor selection, and risk group definition):*  XGBoost | | | |
| *Describe whether and how the model was validated, either internally (e.g. bootstrapping, cross validation, random split sample) or externally (e.g. temporal validation, geographical validation, different setting, different type of participants):*  Cross validation was used. | | | |
| *Describe the performance measures of the model, e.g. (re)calibration, discrimination, (re)classification, net benefit, and whether they were adjusted for optimism:*  The ROC analysis was performed. | | | |
| *Describe any participants who were excluded from the analysis:*  *NI* | | | |
| *Describe missing data on predictors and outcomes as well as methods used for missing data:*  *NI* | | | |
|  | | Dev | Val |
| 1. Were there a reasonable number of participants with the outcome? | | PY |  |
| 1. Were continuous and categorical predictors handled appropriately? | | PY |  |
| 1. Were all enrolled participants included in the analysis? | | PY |  |
| 1. Were participants with missing data handled appropriately? | | NI |  |
| 1. Was selection of predictors based on univariable analysis avoided? | | PY |  |
| 1. Were complexities in the data (e.g. censoring, competing risks, sampling of controls) accounted for appropriately? | | PY |  |
| 1. Were relevant model performance measures evaluated appropriately? | | PN |  |
| 1. Were model overfitting and optimism in model performance accounted for? | | PY |  |
| 1. Do predictors and their assigned weights in the final model correspond to the results from multivariable analysis? | | PY |  |
| **Risk of bias introduced by the analysis** | **RISK:**  *(low/ high/ unclear)* | **high** |  |
| *Rationale of bias rating:*  The model was not evaluated with external validation. The analysis is based on input data from a State (Espírito Santo) of Brazil which may impact the generalizability of the model. | | | |

**Step 4: Overall assessment**

| | **Reaching an overall judgement about risk of bias of the prediction model evaluation** | | | --- | --- | | **Low risk of bias** | If all domains were rated low risk of bias.  If a prediction model was developed without any external validation, and it was rated as low risk of bias for all domains, consider downgrading to **high risk of bias**. Such a model can only be considered as low risk of bias, if the development was based on a very large data set and included some form of internal validation. | | **High risk of bias** | If at least one domain is judged to be at **high risk of bias**. | | **Unclear risk of bias** | If an unclear risk of bias was noted in at least one domain and it was low risk for all other domains. |  | **Reaching an overall judgement about applicability of the prediction model evaluation** | | | --- | --- | | **Low concerns regarding applicability** | If low concerns regarding applicability for all domains, the prediction model evaluation is judged to have **low concerns regarding applicability**. | | **High concerns regarding applicability** | If high concerns regarding applicability for at least one domain, the prediction model evaluation is judged to have **high concerns regarding applicability**. | | **Unclear concerns regarding applicability** | If unclear concerns (but no “high concern”) regarding applicability for at least one domain, the prediction model evaluation is judged to have **unclear concerns regarding applicability** overall. | |
| --- | --- | --- | --- | --- | --- | --- | --- | --- | --- | --- | --- | --- | --- | --- | --- | --- |

| **Overall judgement about risk of bias and applicability of the prediction model evaluation** | | |
| --- | --- | --- |
| **Overall judgement of risk of bias** | **RISK:**  *(low/ high/ unclear)* | **high** |
| *Summary of sources of potential bias:*  *Different covariant assessment windows were not defined for different data types to generate variables.* The time interval between predictors’ assessment and outcome was not defined. | | |
| **Overall judgement of applicability** | **CONCERN:**  *(low/ high/ unclear)* | **high** |
| *Summary of applicability concerns:*  The data of death patients outside of hospitals were not evaluated in the model which reduces its generality. The model was not evaluated with external validation. The analysis is based on input data from a State (Espírito Santo) of Brazil which may impact the generalizability of the model. | | |

**Appendix 28**. Ottenhoff 2021 - Bias analysis using PROBAST.

**Step 1: Specify your systematic review question**

| **Criteria** | **Specify your systematic review question** |
| --- | --- |
| *Intended use of model:* | To predict mortality of hospitalized COVID-19 patients |
| ***Participants*** *including selection criteria and setting:* | Positive tested COVID-19 patients and hospitalized |
| ***Predictors*** *(used in prediction modelling), including types of predictors (e.g. history, clinical examination, biochemical markers, imaging tests), time of measurement, specific measurement issues (e.g., any requirements/ prohibitions for specialized equipment):* |  |
| *Outcome to be predicted:* | Mortality |

**Step 2: Classify the type of prediction model evaluation**

| **Classify the evaluation based on its aim** | | | |
| --- | --- | --- | --- |
| **Type of prediction study** | **PROBAST boxes to complete** | **Tick as appropriate** | **Definition for type of prediction model study** |
| Development only | Development | ✔ | Prediction model development without external validation. These studies may include internal validation methods, such as bootstrapping and cross-validation techniques. |
| Development and validation | Development and validation | ✖ | Prediction model development combined with external validation in other participants in the same article. |
| Validation only | Validation | ✖ | External validation of existing (previously developed) model in other participants. |

|  | |
| --- | --- |
| **Publication reference** | Ottenhoff MC, Ramos LA, Potters W, Janssen MLF, Hubers D, Hu S, Fridgeirsson EA, Piña-Fuentes D, Thomas R, van der Horst ICC, Herff C, Kubben P, Elbers PWG, Marquering HA, Welling M, Simsek S, de Kruif MD, Dormans T, Fleuren LM, Schinkel M, Noordzij PG, van den Bergh JP, Wyers CE, Buis DTB, Wiersinga WJ, van den Hout EHC, Reidinga AC, Rusch D, Sigaloff KCE, Douma RA, de Haan L, Gritters van den Oever NC, Rennenberg RJMW, van Wingen GA, Aries MJH, Beudel M; Dutch COVID-PREDICT research group. Predicting mortality of individual patients with COVID-19: a multicentre Dutch cohort. BMJ Open. 2021 Jul 19;11(7):e047347. doi: 10.1136/bmjopen-2020-047347. PMID: 34281922; PMCID: PMC8290951. | |
| **Models of interest** | XGBOOST |  |
| **Outcome of interest** | Mortality | |

**Step 3: Assess risk of bias and applicability**

| **DOMAIN 1:  Participants** | | | |
| --- | --- | --- | --- |
| **A. Risk of Bias** | | | |
| *Describe the sources of data and criteria for participant selection:*  DEV:  A multicentre cohort across 10 Dutch hospitals including patients who were admitted in a hospital, age ≥18 years, positive Covid-19 test before or during admission, from 27 February to 8 June 2020. The demographic, medicine, clinic,  laboratory at admission time was stored in the database (Castor EDC, Amsterdam, The Netherlands) by each hospital independently. | | | |
|  | | Dev | Val |
| 1. Were appropriate data sources used, e.g. cohort, RCT or nested case-control study data? | | Y |  |
| 1. Were all inclusions and exclusions of participants appropriate? | | PY |  |
| **Risk of bias introduced by selection of participants** | **RISK:**  *(low/ high/ unclear)* | **low** |  |
| *Rationale of bias rating:* | | | |
| No concerns | | | |
| **B. Applicability** | | | |
| *Describe included participants, setting and dates:*  It has been mentioned in Domain 1 – Section A. | | | |
| **Concern that the included participants and setting do not match the review question** | **CONCERN:**  *(low/ high/ unclear)* | **low** |  |
| *Rationale of applicability rating:* | | | |
| In our review, our source population is the general population. Therefore, this data is part of the general population. | | | |

| **DOMAIN 2:  Predictors** | | | |
| --- | --- | --- | --- |
| **A. Risk of Bias** | | | |
| *List and describe predictors included in the final model, e.g. definition and timing of assessment:*  Age, number of home medications, urea nitrogen, lactate dehydrogenase, albumin, oxygen saturation (%), oxygen saturation is measured on room air, oxygen saturation is measured on oxygen therapy, blood gas pH and history of chronic cardiac disease. | | | |
|  | | Dev | Val |
| 1. Were predictors defined and assessed in a similar way for all participants? | | PY |  |
| 1. Were predictor assessments made without knowledge of outcome data? | | PY |  |
| 1. Are all predictors available at the time the model is intended to be used? | | Y |  |
| **Risk of bias introduced by predictors or their assessment** | **RISK:**  *(low/ high/ unclear)* | **low** |  |
| *Rationale of bias rating:* | | | |
| **B. Applicability** | | | |
| Concern that the definition, assessment or timing of predictors in the model do not match the review question | **CONCERN:**  *(low/ high/ unclear)* | **high** |  |
| *Rationale of applicability rating:*  Some useful variables such as d-dimer, presence of infiltrates on the chest X-ray and duration of symptoms before hospital admission were removed due to too many missing values. Additionally, the duration of symptoms before admission was anamnestic, decreasing its reliability due to the retrospective data collection. | | | |

| **DOMAIN 3: Outcome** | | | |
| --- | --- | --- | --- |
| **A. Risk of Bias** | | | |
| *Describe the outcome, how it was defined and determined, and the time interval between predictor assessment and outcome determination:*  The outcome was defined as either death or discharge within 21 days after hospital admission. | | | |
|  | | Dev | Val |
| 1. Was the outcome determined appropriately? | | PY |  |
| 1. Was a pre-specified or standard outcome definition used? | | PY |  |
| 1. Were predictors excluded from the outcome definition? | | Y |  |
| 1. Was the outcome defined and determined in a similar way for all participants? | | PY |  |
| 1. Was the outcome determined without knowledge of predictor information? | | PY |  |
| 1. Was the time interval between predictor assessment and outcome determination appropriate? | | NI |  |
| **Risk of bias introduced by the outcome or its determination** | **RISK:**  *(low/ high/ unclear)* | **unclear** |  |
| *Rationale of bias rating:*  The time interval between predictors’ assessment and outcome was defined without references. | | | |
| **B. Applicability** | | | |
| *At what time point was the outcome determined:*  *If a composite outcome was used, describe the relative frequency/distribution of each contributing outcome:* | | | |
| **Concern that the outcome, its definition, timing or determination do not match the review question** | **CONCERN:**  *(low/ high/ unclear)* | **high** |  |
| *Rationale of applicability rating:*  The outcome was defined as all-cause mortality instead of COVID-19 related mortality, and this might result in an overestimation of the predictive power of specific comorbidities. Furthermore, The time interval, 21 days, between predictors’ assessment and outcome was defined without references. Changing that might cause different results leading to an underestimation of mortality. | | | |
| **DOMAIN 4: Analysis** | | | |
| **Risk of Bias** | | | |
| *Describe numbers of participants, number of candidate predictors, outcome events and events per candidate predictor:*  DEV:   The analytical data included 80 features for 2273 patients, of whom 516 had died or discharged to palliative care within 21 days after admission. | | | |
| *Describe how the model was developed (for example in regards to modelling technique (e.g. survival or logistic modelling), predictor selection, and risk group definition):*  Non-linear tree-based gradient boosting | | | |
| *Describe whether and how the model was validated, either internally (e.g. bootstrapping, cross validation, random split sample) or externally (e.g. temporal validation, geographical validation, different setting, different type of participants):*  Cross validation was used. | | | |
| *Describe the performance measures of the model, e.g. (re)calibration, discrimination, (re)classification, net benefit, and whether they were adjusted for optimism:*  The ROC analysis was performed. | | | |
| *Describe any participants who were excluded from the analysis:*  *Patients that were still hospitalised but shorter than 21 days, transferred to other hospitals (including transfers to participating hospitals), readmitted or have an unknown outcome were excluded from further analysis.* | | | |
| *Describe missing data on predictors and outcomes as well as methods used for missing data:*  *The remaining missing values were imputed using Bayesian ridge regression, which is inspired by the Multivariate Imputation by Chained Equations*  *Features that had more than 50% missing values and subsequently patient records that had more than 80% missing values were removed.* | | | |
|  | | Dev | Val |
| 1. Were there a reasonable number of participants with the outcome? | | PY |  |
| 1. Were continuous and categorical predictors handled appropriately? | | PY |  |
| 1. Were all enrolled participants included in the analysis? | | PY |  |
| 1. Were participants with missing data handled appropriately? | | NI |  |
| 1. Was selection of predictors based on univariable analysis avoided? | | PY |  |
| 1. Were complexities in the data (e.g. censoring, competing risks, sampling of controls) accounted for appropriately? | | PY |  |
| 1. Were relevant model performance measures evaluated appropriately? | | PN |  |
| 1. Were model overfitting and optimism in model performance accounted for? | | PY |  |
| 1. Do predictors and their assigned weights in the final model correspond to the results from multivariable analysis? | | PY |  |
| **Risk of bias introduced by the analysis** | **RISK:**  *(low/ high/ unclear)* | **high** |  |
| *Rationale of bias rating:*  The dataset was extracted during the Covid-19 first wave. It might differ from other time periods due to the availability of therapies like steroids or vaccination. The analysis is based only on input data from Dutch hospitalised patients without external validation. These may impact the generalizability of the model. | | | |

**Step 4: Overall assessment**

| | **Reaching an overall judgement about risk of bias of the prediction model evaluation** | | | --- | --- | | **Low risk of bias** | If all domains were rated low risk of bias.  If a prediction model was developed without any external validation, and it was rated as low risk of bias for all domains, consider downgrading to **high risk of bias**. Such a model can only be considered as low risk of bias, if the development was based on a very large data set and included some form of internal validation. | | **High risk of bias** | If at least one domain is judged to be at **high risk of bias**. | | **Unclear risk of bias** | If an unclear risk of bias was noted in at least one domain and it was low risk for all other domains. |  | **Reaching an overall judgement about applicability of the prediction model evaluation** | | | --- | --- | | **Low concerns regarding applicability** | If low concerns regarding applicability for all domains, the prediction model evaluation is judged to have **low concerns regarding applicability**. | | **High concerns regarding applicability** | If high concerns regarding applicability for at least one domain, the prediction model evaluation is judged to have **high concerns regarding applicability**. | | **Unclear concerns regarding applicability** | If unclear concerns (but no “high concern”) regarding applicability for at least one domain, the prediction model evaluation is judged to have **unclear concerns regarding applicability** overall. | |
| --- | --- | --- | --- | --- | --- | --- | --- | --- | --- | --- | --- | --- | --- | --- | --- | --- |

| **Overall judgement about risk of bias and applicability of the prediction model evaluation** | | |
| --- | --- | --- |
| **Overall judgement of risk of bias** | **RISK:**  *(low/ high/ unclear)* | **unclear** |
| *Summary of sources of potential bias:*  Some useful variables such as d-dimer, presence of infiltrates on the chest X-ray and duration of symptoms before hospital admission were removed due to too many missing values. Additionally, the duration of symptoms before admission was anamnestic, decreasing its reliability due to the retrospective data collection.  The time interval between predictors’ assessment and outcome was defined without references. | | |
| **Overall judgement of applicability** | **CONCERN:**  *(low/ high/ unclear)* | **high** |
| *Summary of applicability concerns:*  Some useful variables such as d-dimer, presence of infiltrates on the chest X-ray and duration of symptoms before hospital admission were removed due to too many missing values. Additionally, the duration of symptoms before admission was anamnestic, decreasing its reliability due to the retrospective data collection.  The outcome was defined as all-cause mortality instead of COVID-19 related mortality, and this might result in an overestimation of the predictive power of specific comorbidities. Furthermore, The time interval, 21 days, between predictors’ assessment and outcome was defined without references. Changing that might cause different results leading to an underestimation of mortality.  The dataset was extracted during the Covid-19 first wave. It might differ from other time periods due to the availability of therapies like steroids or vaccination. The analysis is based only on input data from Dutch hospitalised patients without external validation. These may impact the generalizability of the model. | | |

**Appendix 29**. Mahdavi 2021 - Bias analysis using PROBAST.

**Step 1: Specify your systematic review question**

| **Criteria** | **Specify your systematic review question** |
| --- | --- |
| *Intended use of model:* | To predict mortality of hospitalized COVID-19 patients |
| ***Participants*** *including selection criteria and setting:* | Positive tested COVID-19 patients and hospitalized |
| ***Predictors*** *(used in prediction modelling), including types of predictors (e.g. history, clinical examination, biochemical markers, imaging tests), time of measurement, specific measurement issues (e.g., any requirements/ prohibitions for specialized equipment):* |  |
| *Outcome to be predicted:* | Mortality |

**Step 2: Classify the type of prediction model evaluation**

| **Classify the evaluation based on its aim** | | | |
| --- | --- | --- | --- |
| **Type of prediction study** | **PROBAST boxes to complete** | **Tick as appropriate** | **Definition for type of prediction model study** |
| Development only | Development | ✔ | Prediction model development without external validation. These studies may include internal validation methods, such as bootstrapping and cross-validation techniques. |
| Development and validation | Development and validation | ✖ | Prediction model development combined with external validation in other participants in the same article. |
| Validation only | Validation | ✖ | External validation of existing (previously developed) model in other participants. |

|  | |
| --- | --- |
| **Publication reference** | Mahdavi M, Choubdar H, Zabeh E, Rieder M, Safavi-Naeini S, Jobbagy Z, Ghorbani A, Abedini A, Kiani A, Khanlarzadeh V, Lashgari R, Kamrani E. A machine learning based exploration of COVID-19 mortality risk. PLoS One. 2021 Jul 2;16(7):e0252384. doi: 10.1371/journal.pone.0252384. PMID: 34214101; PMCID: PMC8253432. | |
| **Models of interest** | SVM |  |
| **Outcome of interest** | Mortality | |

**Step 3: Assess risk of bias and applicability**

| **DOMAIN 1:  Participants** | | | |
| --- | --- | --- | --- |
| **A. Risk of Bias** | | | |
| *Describe the sources of data and criteria for participant selection:*  DEV:  The dataset included information of invasive laboratory and noninvasive clinical and demographic data from 492 hospitalized patients (186 patients expired (37.8%)) at Masih Daneshvari Hospital, Tehran, Iran between February 20th, 2020, and May 4th, 2020. | | | |
|  | | Dev | Val |
| 1. Were appropriate data sources used, e.g. cohort, RCT or nested case-control study data? | | Y |  |
| 1. Were all inclusions and exclusions of participants appropriate? | | PY |  |
| **Risk of bias introduced by selection of participants** | **RISK:**  *(low/ high/ unclear)* | **low** |  |
| *Rationale of bias rating: No concerns* | | | |
|  | | | |
| **B. Applicability** | | | |
| *Describe included participants, setting and dates:*  It has been mentioned in Domain 1 – Section A. | | | |
| **Concern that the included participants and setting do not match the review question** | **CONCERN:**  *(low/ high/ unclear)* | **low** |  |
| *Rationale of applicability rating:* | | | |
| In our review, our source population is the general population. Therefore, this data is part of the general population. | | | |

| **DOMAIN 2:  Predictors** | | | |
| --- | --- | --- | --- |
| **A. Risk of Bias** | | | |
| *List and describe predictors included in the final model, e.g. definition and timing of assessment:*  Data from the first 24 hours of patients’ admission was used in this study. The initial data, comprised of 57 features, was categorized into two groups of features; demographic and patient history features were labeled as non-invasive group, and laboratory results were labeled as invasive group. Demographic and history features were extracted from admission history, medical progress notes, and nursing notes. Laboratory features were extracted from the results of the first blood tests, which were ordered by physicians during the initial 24 hours of admission. | | | |
|  | | Dev | Val |
| 1. Were predictors defined and assessed in a similar way for all participants? | | PY |  |
| 1. Were predictor assessments made without knowledge of outcome data? | | PN |  |
| 1. Are all predictors available at the time the model is intended to be used? | | Y |  |
| **Risk of bias introduced by predictors or their assessment** | **RISK:**  *(low/ high/ unclear)* | **high** |  |
| *Rationale of bias rating:*  Researchers were not blind to outcomes | | | |
| **B. Applicability** | | | |
| Concern that the definition, assessment or timing of predictors in the model do not match the review question | **CONCERN:**  *(low/ high/ unclear)* | **high** |  |
| *Rationale of applicability rating:*  The data gathering interval of this study encompassed the first pandemic wave, and medical records were documented in haste as high patient loads and limited medical staff forced the medical system to prioritize patient treatment. Therefore, many patients had incomplete medical profiles and were sieved before the data inspection phase.  Some important features were removed from the analysis due to high missing values owing to limited laboratory resources and incomplete medical records caused by the pandemic. | | | |

| **DOMAIN 3: Outcome** | | | |
| --- | --- | --- | --- |
| **A. Risk of Bias** | | | |
| *Describe the outcome, how it was defined and determined, and the time interval between predictor assessment and outcome determination:*  The outcome was defined as the discharged and expired class.  The discharged group consisted of COVID-19 patients that were discharged after the completion of their treatment and two consecutive negative PCR results. The expired group was patients who died at any point during their treatment course. | | | |
|  | | Dev | Val |
| 1. Was the outcome determined appropriately? | | PY |  |
| 1. Was a pre-specified or standard outcome definition used? | | PY |  |
| 1. Were predictors excluded from the outcome definition? | | Y |  |
| 1. Was the outcome defined and determined in a similar way for all participants? | | PY |  |
| 1. Was the outcome determined without knowledge of predictor information? | | PY |  |
| 1. Was the time interval between predictor assessment and outcome determination appropriate? | | NI |  |
| **Risk of bias introduced by the outcome or its determination** | **RISK:**  *(low/ high/ unclear)* | **unclear** |  |
| *Rationale of bias rating:*  The time interval between predictors’ assessment and outcome was not defined. | | | |
| **B. Applicability** | | | |
| *At what time point was the outcome determined:*  *If a composite outcome was used, describe the relative frequency/distribution of each contributing outcome:* | | | |
| **Concern that the outcome, its definition, timing or determination do not match the review question** | **CONCERN:**  *(low/ high/ unclear)* | **high** |  |
| *Rationale of applicability rating:*  There was no information regarding discharged patients after they left the hospital, they might have died shortly. | | | |
| **DOMAIN 4: Analysis** | | | |
| **Risk of Bias** | | | |
| *Describe numbers of participants, number of candidate predictors, outcome events and events per candidate predictor:*  DEV:   186 patients with an “expired” outcome (37.8%) and 306 patients with “discharged” outcome (62.2%) were included in the study | | | |
| *Describe how the model was developed (for example in regards to modelling technique (e.g. survival or logistic modelling), predictor selection, and risk group definition):*  SVM | | | |
| *Describe whether and how the model was validated, either internally (e.g. bootstrapping, cross validation, random split sample) or externally (e.g. temporal validation, geographical validation, different setting, different type of participants):*  Cross validation was used. | | | |
| *Describe the performance measures of the model, e.g. (re)calibration, discrimination, (re)classification, net benefit, and whether they were adjusted for optimism:*  The ROC analysis was performed. | | | |
| *Describe any participants who were excluded from the analysis:*  *12 patients who left the hospital against medical advice with consent, 27 patients with uncertain or rolled out COVID-19 diagnosis, 29 patients who were referred from other hospitals, 4 patients with age under 18, 43 patients with more than 20% missing data, 18 patients who received radically different treatment protocols (i.e., were enrolled in clinical trials), 1 patient who had a cardiac arrest shortly after arrival to the emergency ward, and 2 pregnant patients were excluded.* | | | |
| *Describe missing data on predictors and outcomes as well as methods used for missing data:*  *Features with more than 10% missing values were entirely omitted. Imputation via the knearest neighbor (KNN) algorithm with k = 5 and uniform weights was used for features with less than 10% missing values; KNN algorithm imputes every missing value using the mean value from ‘k’ closest data points found in the training set.* | | | |
|  | | Dev | Val |
| 1. Were there a reasonable number of participants with the outcome? | | PY |  |
| 1. Were continuous and categorical predictors handled appropriately? | | PY |  |
| 1. Were all enrolled participants included in the analysis? | | PY |  |
| 1. Were participants with missing data handled appropriately? | | NI |  |
| 1. Was selection of predictors based on univariable analysis avoided? | | PY |  |
| 1. Were complexities in the data (e.g. censoring, competing risks, sampling of controls) accounted for appropriately? | | PY |  |
| 1. Were relevant model performance measures evaluated appropriately? | | PN |  |
| 1. Were model overfitting and optimism in model performance accounted for? | | PY |  |
| 1. Do predictors and their assigned weights in the final model correspond to the results from multivariable analysis? | | PY |  |
| **Risk of bias introduced by the analysis** | **RISK:**  *(low/ high/ unclear)* | **high** |  |
| *Rationale of bias rating:*  There was no information regarding discharged patients after they left the hospital, they might have died shortly.  The dataset was extracted during the Covid-19 first wave. It might differ from other time periods due to the availability of therapies like steroids or vaccination. The analysis is based only on input data from private hospital patients without external validation. Since the hospital was a primary care center for COVID-19, the analysis included more severe and expired patients.  These may impact the generalizability of the model. | | | |

**Step 4: Overall assessment**

| | **Reaching an overall judgement about risk of bias of the prediction model evaluation** | | | --- | --- | | **Low risk of bias** | If all domains were rated low risk of bias.  If a prediction model was developed without any external validation, and it was rated as low risk of bias for all domains, consider downgrading to **high risk of bias**. Such a model can only be considered as low risk of bias, if the development was based on a very large data set and included some form of internal validation. | | **High risk of bias** | If at least one domain is judged to be at **high risk of bias**. | | **Unclear risk of bias** | If an unclear risk of bias was noted in at least one domain and it was low risk for all other domains. |  | **Reaching an overall judgement about applicability of the prediction model evaluation** | | | --- | --- | | **Low concerns regarding applicability** | If low concerns regarding applicability for all domains, the prediction model evaluation is judged to have **low concerns regarding applicability**. | | **High concerns regarding applicability** | If high concerns regarding applicability for at least one domain, the prediction model evaluation is judged to have **high concerns regarding applicability**. | | **Unclear concerns regarding applicability** | If unclear concerns (but no “high concern”) regarding applicability for at least one domain, the prediction model evaluation is judged to have **unclear concerns regarding applicability** overall. | |
| --- | --- | --- | --- | --- | --- | --- | --- | --- | --- | --- | --- | --- | --- | --- | --- | --- |

| **Overall judgement about risk of bias and applicability of the prediction model evaluation** | | |
| --- | --- | --- |
| **Overall judgement of risk of bias** | **RISK:**  *(low/ high/ unclear)* | **high** |
| *Summary of sources of potential bias:*  Researchers were not blind to outcomes | | |
| **Overall judgement of applicability** | **CONCERN:**  *(low/ high/ unclear)* | **high** |
| *Summary of applicability concerns:*  The data gathering interval of this study encompassed the first pandemic wave, and medical records were documented in haste as high patient loads and limited medical staff forced the medical system to prioritize patient treatment. Therefore, many patients had incomplete medical profiles and were sieved before the data inspection phase.  Some important features were removed from the analysis due to high missing values owing to limited laboratory resources and incomplete medical records caused by the pandemic.  The dataset was extracted during the Covid-19 first wave. It might differ from other time periods due to the availability of therapies like steroids or vaccination. The analysis is based only on input data from private hospital patients without external validation. Since the hospital was a primary care center for COVID-19, the analysis included more severe and expired patients.  These may impact the generalizability of the model. | | |

**Appendix 30**. Jamshidi 2022 - Bias analysis using PROBAST.

**Step 1: Specify your systematic review question**

| **Criteria** | **Specify your systematic review question** |
| --- | --- |
| *Intended use of model:* | To predict mortality of hospitalized COVID-19 patients |
| ***Participants*** *including selection criteria and setting:* | Positive tested COVID-19 patients and hospitalized |
| ***Predictors*** *(used in prediction modelling), including types of predictors (e.g. history, clinical examination, biochemical markers, imaging tests), time of measurement, specific measurement issues (e.g., any requirements/ prohibitions for specialized equipment):* |  |
| *Outcome to be predicted:* | Mortality |

**Step 2: Classify the type of prediction model evaluation**

| **Classify the evaluation based on its aim** | | | |
| --- | --- | --- | --- |
| **Type of prediction study** | **PROBAST boxes to complete** | **Tick as appropriate** | **Definition for type of prediction model study** |
| Development only | Development | ✔ | Prediction model development without external validation. These studies may include internal validation methods, such as bootstrapping and cross-validation techniques. |
| Development and validation | Development and validation | ✖ | Prediction model development combined with external validation in other participants in the same article. |
| Validation only | Validation | ✖ | External validation of existing (previously developed) model in other participants. |

|  | |
| --- | --- |
| **Publication reference** | Jamshidi E, Asgary A, Tavakoli N, Zali A, Dastan F, Daaee A, Badakhshan M, Esmaily H, Jamaldini SH, Safari S, Bastanhagh E, Maher A, Babajani A, Mehrazi M, Sendani Kashi MA, Jamshidi M, Sendani MH, Rahi SJ, Mansouri N. Symptom Prediction and Mortality Risk Calculation for COVID-19 Using Machine Learning. Front Artif Intell. 2021 Jun 22;4:673527. doi: 10.3389/frai.2021.673527. PMID: 34250465; PMCID: PMC8262614. | |
| **Models of interest** | Logistic Regression, Random Forest, Artificial Neural Network (ANN), K-Nearest Neighbors (KNN), Linear Discriminant Analysis (LDA), and Naive Bayes. |  |
| **Outcome of interest** | Mortality | |

**Step 3: Assess risk of bias and applicability**

| **DOMAIN 1:  Participants** | | | |
| --- | --- | --- | --- |
| **A. Risk of Bias** | | | |
| *Describe the sources of data and criteria for participant selection:*  DEV:  The database was obtained from Hospital Information System (HIS) including 74 hospitals across Tehran, Iran.  The eligibility criteria were defined as confirmed or suspected SARS-CoV-2 infections of people aged 18–100 years registered in the referred HIS. | | | |
|  | | Dev | Val |
| 1. Were appropriate data sources used, e.g. cohort, RCT or nested case-control study data? | | Y |  |
| 1. Were all inclusions and exclusions of participants appropriate? | | NI |  |
| **Risk of bias introduced by selection of participants** | **RISK:**  *(low/ high/ unclear)* | **high** |  |
| *Rationale of bias rating:* | | | |
| *There is no information in dataset for patients who left the hospital, patients with uncertain or rolled out COVID-19 diagnosis, patients who were referred from other hospitals, patients with missing data, patients who received radically different treatment protocols , patients who had died shortly after arrival, and pregnant patients* | | | |
| **B. Applicability** | | | |
| *Describe included participants, setting and dates:*  It has been mentioned in Domain 1 – Section A. | | | |
| **Concern that the included participants and setting do not match the review question** | **CONCERN:**  *(low/ high/ unclear)* | **high** |  |
| *Rationale of applicability rating: See above.* | | | |
|  | | | |

| **DOMAIN 2:  Predictors** | | | |
| --- | --- | --- | --- |
| **A. Risk of Bias** | | | |
| *List and describe predictors included in the final model, e.g. definition and timing of assessment:*  The study included 23,749 covid-19 patients (2,440 deaths (10.27%)) hospitalized between February 1, 2020, and September 30, 2020. The predictors used for the models were age, sex, and past chronic diseases history of the patients. | | | |
|  | | Dev | Val |
| 1. Were predictors defined and assessed in a similar way for all participants? | | PY |  |
| 1. Were predictor assessments made without knowledge of outcome data? | | PY |  |
| 1. Are all predictors available at the time the model is intended to be used? | | Y |  |
| **Risk of bias introduced by predictors or their assessment** | **RISK:**  *(low/ high/ unclear)* | **low** |  |
| *Rationale of bias rating:* | | | |
| **B. Applicability** | | | |
| Concern that the definition, assessment or timing of predictors in the model do not match the review question | **CONCERN:**  *(low/ high/ unclear)* | **high** |  |
| *Rationale of applicability rating:*  The data gathering interval of this study encompassed the first pandemic wave, and medical records were documented in haste as high patient loads and limited medical staff forced the medical system to prioritize patient treatment. Therefore, some patients might have incomplete medical profiles and were sieved before the data inspection phase. | | | |

| **DOMAIN 3: Outcome** | | | |
| --- | --- | --- | --- |
| **A. Risk of Bias** | | | |
| *Describe the outcome, how it was defined and determined, and the time interval between predictor assessment and outcome determination:*  Two outcomes defined in this study. The first outcome is the patients’ symptoms at the time of admission, and clustered in 12 categories such as cough, loss of smell or taste, respiratory distress, vertigo, muscular pain or fatigue, sore throat, fever or chill, paresis or paralysis, gastrointestinal problems, headache, chest pain, and consciousness disorders. The second outcome is death or survival during the study period. | | | |
|  | | Dev | Val |
| 1. Was the outcome determined appropriately? | | PY |  |
| 1. Was a pre-specified or standard outcome definition used? | | PY |  |
| 1. Were predictors excluded from the outcome definition? | | Y |  |
| 1. Was the outcome defined and determined in a similar way for all participants? | | PY |  |
| 1. Was the outcome determined without knowledge of predictor information? | | PY |  |
| 1. Was the time interval between predictor assessment and outcome determination appropriate? | | NI |  |
| **Risk of bias introduced by the outcome or its determination** | **RISK:**  *(low/ high/ unclear)* | **unclear** |  |
| *Rationale of bias rating:*  The dataset was collected by the HIS, therefore it included COVID-19 patients with major symptoms and higher chance of death. This could explain the high mortality rate in the study. | | | |
| **B. Applicability** | | | |
| *At what time point was the outcome determined:*  *If a composite outcome was used, describe the relative frequency/distribution of each contributing outcome:* | | | |
| **Concern that the outcome, its definition, timing or determination do not match the review question** | **CONCERN:**  *(low/ high/ unclear)* | **high** |  |
| *Rationale of applicability rating:*  There was no information regarding discharged patients after they left the hospital, they might have died shortly. | | | |
| **DOMAIN 4: Analysis** | | | |
| **Risk of Bias** | | | |
| *Describe numbers of participants, number of candidate predictors, outcome events and events per candidate predictor:*  DEV:   The study included 23,749 covid-19 patients (2,440 deaths (10.27%))  with 15 predictors | | | |
| *Describe how the model was developed (for example in regards to modelling technique (e.g. survival or logistic modelling), predictor selection, and risk group definition):*  Logistic Regression, Random Forest, Artificial Neural Network (ANN), K-Nearest Neighbors (KNN), Linear Discriminant Analysis (LDA), and Naive Bayes. | | | |
| *Describe whether and how the model was validated, either internally (e.g. bootstrapping, cross validation, random split sample) or externally (e.g. temporal validation, geographical validation, different setting, different type of participants):*  Cross validation was used. | | | |
| *Describe the performance measures of the model, e.g. (re)calibration, discrimination, (re)classification, net benefit, and whether they were adjusted for optimism:*  The ROC analysis was performed. | | | |
| *Describe any participants who were excluded from the analysis:*  *NI* | | | |
| *Describe missing data on predictors and outcomes as well as methods used for missing data:*  *NI* | | | |
|  | | Dev | Val |
| 1. Were there a reasonable number of participants with the outcome? | | PY |  |
| 1. Were continuous and categorical predictors handled appropriately? | | PY |  |
| 1. Were all enrolled participants included in the analysis? | | PY |  |
| 1. Were participants with missing data handled appropriately? | | NI |  |
| 1. Was selection of predictors based on univariable analysis avoided? | | PY |  |
| 1. Were complexities in the data (e.g. censoring, competing risks, sampling of controls) accounted for appropriately? | | PY |  |
| 1. Were relevant model performance measures evaluated appropriately? | | PN |  |
| 1. Were model overfitting and optimism in model performance accounted for? | | PY |  |
| 1. Do predictors and their assigned weights in the final model correspond to the results from multivariable analysis? | | PY |  |
| **Risk of bias introduced by the analysis** | **RISK:**  *(low/ high/ unclear)* | **high** |  |
| *Rationale of bias rating:*  There was no information regarding discharged patients after they left the hospital, they might have died shortly.  The dataset was extracted during the Covid-19 first wave. It might differ from other time periods due to the availability of therapies like steroids or vaccination. The analysis is based only on input data from hospitals in Tehran, Iran without external validation.  These may impact the generalizability of the model. | | | |

**Step 4: Overall assessment**

| | **Reaching an overall judgement about risk of bias of the prediction model evaluation** | | | --- | --- | | **Low risk of bias** | If all domains were rated low risk of bias.  If a prediction model was developed without any external validation, and it was rated as low risk of bias for all domains, consider downgrading to **high risk of bias**. Such a model can only be considered as low risk of bias, if the development was based on a very large data set and included some form of internal validation. | | **High risk of bias** | If at least one domain is judged to be at **high risk of bias**. | | **Unclear risk of bias** | If an unclear risk of bias was noted in at least one domain and it was low risk for all other domains. |  | **Reaching an overall judgement about applicability of the prediction model evaluation** | | | --- | --- | | **Low concerns regarding applicability** | If low concerns regarding applicability for all domains, the prediction model evaluation is judged to have **low concerns regarding applicability**. | | **High concerns regarding applicability** | If high concerns regarding applicability for at least one domain, the prediction model evaluation is judged to have **high concerns regarding applicability**. | | **Unclear concerns regarding applicability** | If unclear concerns (but no “high concern”) regarding applicability for at least one domain, the prediction model evaluation is judged to have **unclear concerns regarding applicability** overall. | |
| --- | --- | --- | --- | --- | --- | --- | --- | --- | --- | --- | --- | --- | --- | --- | --- | --- |

| **Overall judgement about risk of bias and applicability of the prediction model evaluation** | | |
| --- | --- | --- |
| **Overall judgement of risk of bias** | **RISK:**  *(low/ high/ unclear)* | **high** |
[truncated: 102,421 more chars]
